# MHCVision: estimation of global and local false discovery rate for MHC class I peptide binding prediction

**DOI:** 10.1093/bioinformatics/btab479

**Published:** 2021-07-01

**Authors:** Phorutai Pearngam, Sira Sriswasdi, Trairak Pisitkun, Andrew R Jones

**Affiliations:** Program in Bioinformatics and Computational Biology, Graduate School, Chulalongkorn University, Bangkok 10330, Thailand; Institute of Systems, Molecular and Integrative Biology, University of Liverpool, Liverpool L69 7ZB, UK; Research Affairs, Faculty of Medicine, Chulalongkorn University, Bangkok 10330, Thailand; Computational Molecular Biology Group, Faculty of Medicine, Chulalongkorn University, Bangkok 10330, Thailand; Center of Excellence in Systems Biology, Faculty of Medicine, Chulalongkorn University, Bangkok 10330, Thailand; Institute of Systems, Molecular and Integrative Biology, University of Liverpool, Liverpool L69 7ZB, UK

## Abstract

**Motivation:**

MHC-peptide binding prediction has been widely used for understanding the immune response of individuals or populations, each carrying different MHC molecules as well as for the development of immunotherapeutics. The results from MHC-peptide binding prediction tools are mostly reported as a predicted binding affinity (IC_50_) and the percentile rank score, and global thresholds e.g. IC_50_ value < 500 nM or percentile rank < 2% are generally recommended for distinguishing binding peptides from non-binding peptides. However, it is difficult to evaluate statistically the probability of an individual peptide binding prediction to be true or false solely considering predicted scores. Therefore, statistics describing the overall global false discovery rate (FDR) and local FDR, also called posterior error probability (PEP) are required to give statistical context to the natively produced scores.

**Result:**

We have developed an algorithm and code implementation, called MHCVision, for estimation of FDR and PEP values for the predicted results of MHC-peptide binding prediction from the NetMHCpan tool. MHCVision performs parameter estimation using a modified expectation maximization framework for a two-component beta mixture model, representing the distribution of true and false scores of the predicted dataset. We can then estimate the PEP of an individual peptide’s predicted score, and conversely the probability that it is true. We demonstrate that the use of global FDR and PEP estimation can provide a better trade-off between sensitivity and precision over using currently recommended thresholds from tools.

**Availability and implementation:**

https://github.com/PGB-LIV/MHCVision.

**Supplementary information:**

Supplementary data are available at *Bioinformatics* online.

## 1 Introduction

The host immune system can respond to the appearance of pathogenic fragments or mutated peptides derived from cancer cells by the process of antigen presentation. As part of the antigen processing pathway, short peptide fragments are generated from either cytosolic proteins or extracellular proteins that enter cells via the vesicular system. Digested peptides are subsequently presented by major histocompatibility (MHC) proteins, called human leucocyte antigens (HLA) for human. Peptides from cytosolic proteins can be presented by MHC class I molecules (HLA-A, -B and -C) and recognized by cytotoxic CD8+ T cells. Those peptides derived from the endocytic processing pathway are bound to MHC class II (HLA-DR, -DQ and -DP) and can activate CD4+ T cells (Unanue, 2006). A strong interaction between peptides and MHC molecules is a fundamental step to initiate the activation of T cells and subsequently initiate an adaptive immune system to eliminate or attack the source of foreign peptides (Wieczorek *et al.*, 2017). The diversity of MHC molecules is high due to extensive polymorphism at most loci. The latest update of IMGT/HLA database in August 2019 contains 24 093 alleles including 16 943 alleles of HLA-A, -B and -C and 6650 alleles of HLA class II (Robinson *et al.*, 2020). Many of the alleles are exceptionally rare, carried only by a few individuals, but 1122 alleles of HLA-A, -B, -C, -DRB, -DQA, -DQB, -DPA and -DPB loci are common and well-documented, 415 alleles of these alleles were identified as ‘common’ (having known frequencies) and 707 as ‘well-documented’ base on HLA genotyping observations and available HLA haplotype data (Mack *et al.*, 2013). Each HLA allele has a binding preference to specific peptides, driven mostly by the physicochemical properties of their primary sequence. Experimental work, called ‘immunopeptidomics’, for example using mass spectrometry (MS) to identify the set of MHC-bound peptides eluted from cells, has been carried out for many alleles, but can only ever identify a subset of the true total peptide binding potential for a given allele (Yewdell and Bennink, 1999). The accuracy of identification of binding peptides of a specific HLA allele using computational methods is therefore highly useful for understanding individual or population-specific immune response, and facilitating the development of clinical immunotherapy such as vaccines for infectious diseases or for cancer via neoepitope prediction (Zhang *et al.*, 2012).

Computational methods for class I MHC-peptide binding prediction showed high accuracy and have been reported to have a better performance than their MHC II counterparts (Nielsen *et al.*, 2008; Wang *et al.*, 2008). Moreover, when compared with MHC I binding prediction, MHC II predictors have a high risk of falsely excluding strong binders (Zhao and Sher, 2018). The current benchmark of HLA class I binding prediction showed the best performance of 90% sensitivity and 98% specificity (Nielsen and Andreatta, 2016). In this study, we therefore focus on peptide binding prediction against HLA class I because there are more available experimental datasets, and accurately predicted results are crucial for the robustness of the developed model (Table 1). In general, the prediction software include a machine learning-based algorithm, trained with experimental data for peptides known to be bound or not bound by specific HLA alleles. Among recent publicly available tools, there are two tools, NetMHCpan and MHCflurry (O'Donnell *et al.*, 2018; Reynisson *et al.*, 2020), which perform consistently well in benchmarking studies (Paul *et al.*, 2020). NetMHCpan4.1 is the latest version of NetMHCpan family, it extends the training using neural networks that can learn connections between amino acid positions in the HLA protein sequence and target peptides, and thus can make predictions for HLA alleles in the absence of specific data for one given allele. Furthermore, this current version covers 2915 MHC molecules for HLA-A, -B and -C, but only 79 alleles are supported in MHCflurry. Our initial focus thus studied the prediction results by using NetMHCpan4.1, although we also comment on the extensibility of our approach to MHCflurry.

**Table 1.** MHC class I-peptide binding prediction tools

Tools	Reported IC_50_ (nM)	Reported % Rank	Supported HLA I alleles
NetMHC 4.0 (Andreatta and Nielsen, 2016)	✓	✓	80
NetMHCpan 4.1 (Reynisson *et al.*, 2020)	✓	✓	2915
NetMHCcons 1.1	✓	✓	94–120
(Karosiene *et al.*, 2012)			
MHCflurry 2.0 (O'Donnell *et al.*, 2018)	✓	✓	79
MHCnuggets 2.3 (Shao *et al.*, 2020)	✓	✗	148
MHCSeqNet	Binding probability		65
(Phloyphisut *et al.*, 2019)	(0–1)		
EDGE (Sarkizova *et al.*, 2020)	✗	✗	53

The output from a prediction reports the predicted binding affinity (IC_50_) in nM unit. IC_50_ < 500 nM is the commonly recommended threshold for binding affinity to classify binding and non-binding peptides. From version 4.0 onward, NetMHCpan also produces a percentile rank (‘% Rank’) for each predicted IC_50_, the % rank scores were determined by comparing a given IC_50_ value for one peptide to the score distribution of a large selection of random peptides. The percent rank < 2% is a suggested threshold for distinguishing binding and non-binding peptides rather than IC_50_ < 500 nM because it reduces the bias of binding preference across different MHC molecules (Jurtz *et al.*, 2017). Given that most researchers are more interested in statistics that relate to the proportion of true detections at a given threshold, we believe that peptide binding predictions require the estimation of false discovery rate (FDR), which is one of the motivations for this work. Moreover, the posterior error probability (PEP), also known as local FDR, is critical for evaluating the probability that each individual data point is false (the converse of PEP is the true probability) (Käll *et al.*, 2008). To estimate FDR and PEP accurately, the distributions of the true and false data points must be known or reliably estimated. In this work, we have developed a new software package called MHCVision to estimate these distributions and calculate PEP and FDR from peptide binding prediction data. We have trained and optimized MHCVision using several datasets where we have engineered a mixture of *a priori* known ‘true’ and ‘false’ data, and then we demonstrate that this works in practice on a wider range of artificial mixtures (with a known answer) and produces highly plausible estimates on real datasets where the underlying ground truth is unknown.

## 2 Materials and methods

**Fig. 1.**
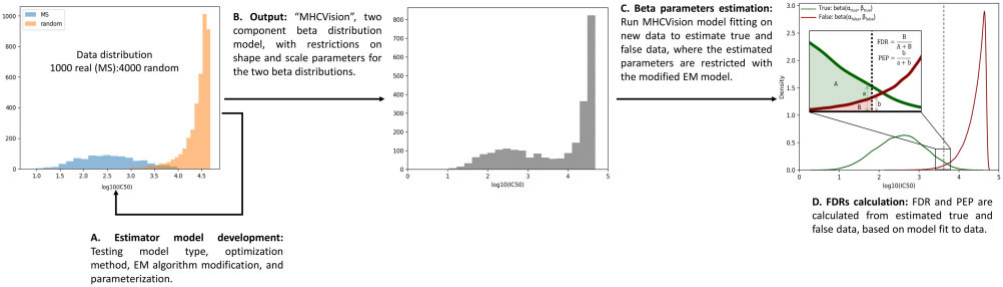
The development of beta parameter estimation model and MHCVision workflow. (**A**) The beta parameter estimation model is developed by using the EM algorithm, and testing different data distributions for matching against a mixture of true binding and random peptides. (**B**) The output from the model development is ‘MHCVision’, which is a parameter estimation model for two component of beta distributions, where estimated beta parameters are constrained based on observations of plausible ranges for binding affinities. (**C**) The estimated beta parameters corresponding to true and false data are estimated by MHCVision, those parameters are constrained within plausible ranges using the modified EM algorithm. (**D**) The values of FDR and PEP are calculated from estimated parameters from the densities of fitted beta distribution

Figure 1 provides an overview of the process followed to develop MHCVision and briefly summarized here. First, we obtained data (predicted IC_50_ scores) from multi-allelic cells and from mono-allelic cells, overlaid with scores from random peptides to evaluate the best fitting data distributions for true and false data (Fig. 1A, Sections 2.1 and 2.2). These analyses demonstrated that two beta distributions, with some parameter constraints, well model true and false data. Then, the beta parameter estimator model was developed using the modified expectation maximization (EM) algorithm, as described in Section 2.3 (Schröder and Rahmann, 2017). The robustness of the model was evaluated by running the model on a variety of datasets where true and false data are known and comparing the similarity of simulated data generated from the model parameters against the real data (Fig. 1B). The MHCVision model fits beta mixture distributions to a given dataset, to estimate beta parameters and relative sizes of true and false data, and thus enables the calculation of FDR and PEP for each peptide’s predicted binding affinity (Fig. 1C, Section 2.4). The method for evaluating model fit on a wider range of testing data is covered in Section 2.5.

### 2.1 MHC binding peptide collection

Datasets of MHC-bound peptides derived from MS analysis were downloaded from the Immuno Epitope Database and Analysis Resource (IEDB, https://www.iedb.org/) (Robinson *et al.*, 2020). Human peptides identified from MS and bound to HLA-A, -B and -C were collected. Other eluted peptides from MHC class I ‘mono-allelic cells’, i.e. presented peptides from cells carrying a single HLA allele, were collected from several publications of immunopeptidomics studies (Abelin *et al.*, 2017; Sarkizova *et al.*, 2020; Schittenhelm *et al.*, 2014; Solleder *et al.*, 2020). Peptides for each HLA allele from those sources were combined, and redundant peptides were removed. Only peptides with lengths of 8, 9, 10 and 11 mer were retained. However, the majority of peptide length in the collected data was found as 9mer peptides for all HLA alleles. HLA alleles that had ≥1000 9mer peptides were collected for onward analysis, totalling 85 HLA alleles covering HLA-A, -B and -C (Supplementary Table S1, sheet1). Additionally, the ‘multi-allelic datasets’, i.e. a set of peptides presented by cells carrying several alleles, were obtained from the dataset contains naturally presented HLA class I ligands derived from chronic myeloid leukemia (CML) patients (Bilich *et al.*, 2019).

To create datasets for training and testing MHCVision, we required datasets with a known answer, containing biologically plausible mixtures of true and false binders. The true binders were sourced from the 85 mono-allelic datasets containing mass spectrometry (MS) datasets where peptides presented by genuine MHC alleles were presented. False data points were modelled by random peptides, created from the human proteome in the UniProt database (www.uniprot.org) by a sliding window approach to generate peptides of 8, 9, 10 and 11 amino acids (Apweiler *et al.*, 2004). To generate a biologically plausible mixture of scores of MS and random peptides, the dataset of MS peptides derived from mono-allelic samples was combined with random peptides (in a ratio of 1000:4000). It should be noted that since some random peptides may be true binders, we exclude random peptides with IC_50_ < 1000 nM (∼2.5% of all generated random peptides on average) from consideration (Supplementary Fig. S1). The resulting peptide set was run through NetMHCpan4.1 stand-alone package via the command line, for each specific HLA allele. These mixture datasets were used for the learning phase of developing MHCVision, and then for testing that accurate values of FDR and PEP were estimated, along with a wider range of testing sets on which the model had not been previously trained (Section 2.4).

### 2.2 Evaluation of fitted statistical distributions

To find statistical models that can properly model the observed bimodal distributions, the data for 85 alleles (mixtures of peptides identified from MS data and random peptides) in a 1000:4000 ratio were inspected and tested for fit against Gaussian and beta distributions. Simulated datasets of 85 HLA alleles were generated by beta and normal functions with specific values of parameters using numpy packages in python, which are numpy.random.beta (α, β, size) and numpy.random.normal (μ, σ, size). The parameters for generating simulated data were calculated from each dataset. As the beta distribution lies the interval [0, 1], we accordingly scaled the binding affinity scores (log_10_(IC_50_)) for peptides in each dataset to the same interval by dividing the predicted scores by the maximum value. Then, the similarity between the real and simulated data was evaluated by the *R*^2^ value calculated from the linear regression analysis.

### 2.3 The modified EM algorithm with the iterated method of moments for the mixture model

The parameter estimation algorithm for a mixture of two distributions was built using a python script. The detailed description that follows is for the final implementation of the algorithm in MHCVision for a mixture of two beta distributions. The algorithm was proceeded iteratively as in the basis of the EM algorithm. The step of parameter estimation was computed by Pearson’s method of moment instead of the maximization of expected likelihood, thus, the maximization step (M-step) was replaced by a method of moments estimation step (MM-step) (Schröder and Rahmann, 2017). For each iteration, parameters including two mixture proportions $\left(\pi_{1},\;\pi_{2}\right)$, two means ${(\mu }_{1},\;\mu_{2})$ and two variance values $\left(\sigma_{1}^{2},\;\sigma_{2}^{2}\right)$ were estimated for two components.

#### 2.3.1 Initialization

As the distribution of predicted scores was bimodal, thus, two was defined as number of components. The data was sorted by predicted IC_50_ and divided in half, thus, the initial π of each component was initially set as 0.5. The initial $\mu_{j},\;\sigma_{j}^{2}$ were calculated from the data of each component *j*, and the initial values of $\alpha_{j}$ and $\beta_{j}$ were then computed according to Equations 1 and 2.

(1) $$\alpha_{j}=\left(\frac{1-\mu_{j}}{\sigma_{j}^{2}}-\frac{1}{\mu_{j}}\right)\mu_{j}^{2}$$

 

(2) $$\beta_{j}=\alpha_{j}(\frac{1}{\mu_{j}}-1)$$

#### 2.3.2 Expectation step (E-step)

The expected responsibility weight (W_i, j_) of each component *j* and data point x_i_ was estimated from the probability density function of the current estimates for beta distributions $(\alpha_{j}^{t}$, $\beta_{j}^{t})\;$and the mixture proportion$\;\pi_{j}^{t}$ (Equation 3).

(3) $$W_{i,j}^{t}=\frac{\pi_{j}^{t}\left(f\left(x_{i};\;{\;\alpha }_{j}^{t},\beta_{j}^{t}\right)\right)}{\sum_{j=1}^{k}\pi_{j}^{t}f\left(x_{i};\;\alpha_{j}^{t},\beta_{j}^{t}\right)},$$

 

$$\text{where}\;f(x;\;\alpha ,\beta )=\frac{\Gamma \left(\alpha +\beta \right)}{\Gamma \left(\alpha \right)\Gamma \left(\beta \right)}\cdot x^{\alpha -1}\cdot {\left(1-x\right)}^{\beta -1}$$

#### 2.3.3 Method of moments estimation (MM-step)

For each component *j*, the $\pi_{j}$ is updated based on the new values of $W_{i,j}^{t}$ according to Equation 4. Then, the component’s mean and variance and the beta distribution parameters are updated using the method of moments (Equations 5–8).

(4) $${\pi^{t+1}}_{j}=\;\frac{1}{n}\sum_{i=1}^{n}W_{i,j}^{t}$$

 

(5) $$\mu_{j}^{t+1}=\frac{\sum_{i=1}^{n}W_{i,j}^{t}\cdot x_{i}\;}{\sum_{i=1}^{n}W_{i,j}^{t}}$$

 

(6) $$\begin{array}{c} {\left(\sigma_{j}^{2}\right)}^{t+1} \end{array}=\frac{\sum_{i=1}^{n}W_{i,j}^{t}\cdot {\left(x_{i}-\mu_{j}^{t+1}\right)}^{2}}{\sum_{i=1}^{n}W_{i,j}^{t}}$$

 

(7) $$\alpha_{j}^{t+1}=\left(\frac{1-\mu_{j}^{t+1}}{{\left(\sigma_{j}^{2}\right)}^{t+1}}-\frac{1}{\mu_{j}^{t+1}}\right){\left(\mu_{j}^{2}\right)}^{t+1}$$

 

(8) $$\beta_{j}^{t+1}=\alpha_{j}^{t+1}\left(\frac{1}{\mu_{j}^{t+1}}-1)\right)$$

In this step, we further constrained the estimated beta parameters for the beta 2 component by the ranges of values calculated from the datasets of various sizes (1000, 5000, 10000) of predicted binding affinity scores from random peptides with a length of 8, 9, 10 and 11 mer against 85 HLA alleles. The purpose of this restriction was to ensure that the beta 2 component of the mixture model is certain to capture the false data. Since ultimately the model for MHCVision fits to the distribution of data observed, we believe this step does not bias the estimation of the ‘false’ distribution but will lead to more accurate estimate of the shape of the genuinely false distribution when new data is tested. Moreover, to ensure the beta 1 component is not fitted to the wrong distribution when presented with *all false data*, the estimated parameters of the first component are restricted if the estimated $\pi_{1}$= 0 and size of the negative set ≠ 0 (predicted IC_50_ > 10 000 nM) i.e. indicating that there is only one distribution found, and there are data points in the plausible range for false data. In this case, the ranges of α and β for the first beta component were initially calculated from data points with predicted IC_50_  ≤ 10 000 nM using Equations 1 and 2, and the range of values are only allowed to deviate 25% from the initial estimates. In practice, these two constraints mean that when the algorithm detects evidence a very large imbalance, in either direction (i.e. all true or all false), the beta 1 or beta 2 is correctly fitted to the appropriate distribution.

#### 2.3.4 Termination

The estimations (E-step and MM-step) were repeated until the maximal relative changes in the estimated parameter values, *k^t^*, between step *t* and *t *+* *1 is less than 0.00001 (Equation 9).

(9) $$k^{t}=\max\left(\left\{\frac{\left|\alpha_{j}^{t+1}-\alpha_{j}^{t}\right|}{\max\left(\left|\alpha_{j}^{t+1}\right|,\;\left|\alpha_{j}^{t}\right|\right)},\;\frac{\left|\beta_{j}^{t+1}-\beta_{j}^{t}\right|}{\max\left(\left|\beta_{j}^{t+1}|,|\beta_{j}^{t}\right|\right)},\frac{\left|\pi_{j}^{t+1}-\pi_{j}^{t}\right|}{\max\left(\left|\pi_{j}^{t+1}|,|\pi_{j}^{t}\right|\right)}\right\}\right)$$

### 2.4 Calculation of FDR and PEP for predicted scores

The estimated beta parameters were utilized to calculate values of FDR and PEP of an individual predicted score in the dataset using Equations 10 and 11, respectively. The number of false and true positive were estimated by the cumulative distribution function of the beta distribution while density at true and false were estimated by the probability density function of the beta distribution.

$$\mathrm{Given}\;\mathrm{data}\;X=\left(x_{1},\ldots .,x_{n}\right)$$

 

(10) $$FDR_{x_{i}}=\left.\frac{F_{\alpha_{\mathrm{false}},\beta_{\mathrm{false}}(x_{i})}}{F_{\alpha_{\mathrm{true}},\beta_{\mathrm{true}}(x_{i})}+F_{\alpha_{\mathrm{false}},\beta_{\mathrm{false}}(x_{i})}}\;\right.$$

 

(11) $$PEP_{x_{i}}=\frac{f_{\alpha_{\mathrm{false}},\beta_{\mathrm{false}}(x_{i})}}{f_{\alpha_{\mathrm{true}},\beta_{\mathrm{true}}(x_{i})}+f_{\alpha_{\mathrm{false}},\beta_{\mathrm{false}}(x_{i})}}$$

### 2.5 Evaluation of fitted mixture model estimates

We evaluated the performance of the modified EM algorithm for the beta mixture model using the original ‘training’ data (for 85 alleles, mixtures of MS data and random peptides) in a 1000:4000 ratio, as well as additional sets (1000:8000 ratios), all true (1000 MS peptides) and all false (4000 MS peptides). For these datasets, we next generated simulated data, based on the beta distribution parameters learnt from the model fitting via the modified EM process described above. The purpose of the simulated data was only to test for the quality of the fit between simulated and real data. Two components including estimated true and false data were generated with estimated parameters given from the EM model for each HLA allele. The best estimated beta parameters for two components produced by the modified EM method above were used to generate the simulated distributions. The simulated true data was generated by a set of estimated true parameters ($\alpha_{\mathrm{true}}$, $\beta_{\mathrm{true}}{\;\pi }_{\mathrm{true}}$), and the simulated false data was created by a set of estimated false parameters ($\alpha_{\mathrm{false}}$, $\beta_{\mathrm{false}}$,$\pi_{\mathrm{false}}$), then, simulated true and false were concatenated.

$$\mathrm{Simulated}\;\mathrm{true}\;\mathrm{data}\;=\;\mathrm{numpy}.\mathrm{random}.\mathrm{beta}(\alpha_{\mathrm{true}},\beta_{\mathrm{true}},(\pi_{\mathrm{true}}*\mathrm{size}))$$

$$\mathrm{Simulated}\;\mathrm{false}\;\mathrm{data}\;=\;\mathrm{numpy}.\mathrm{random}.\mathrm{beta}(\alpha_{\mathrm{false}},\beta_{\mathrm{false}},(\pi_{\mathrm{false}}*\mathrm{size}))$$

The similarity between real and simulated data generated by estimated parameters was measured by the linear regression analysis yielding *R*^2^ statistics. Moreover, Kolmogorov–Smirnov (KS) test was used to detect the difference between the real and simulated datasets, the significant threshold was set at *P*-value < 0.05.

## 3 Results

### 3.1 The data distribution of binding and non-binding peptide scores and model fitting

**Fig. 2.**
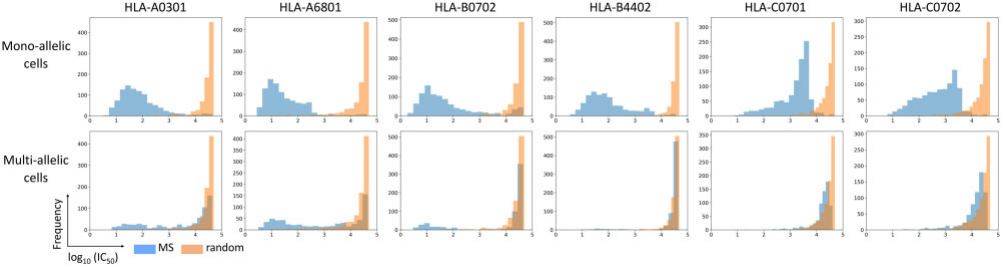
The distribution of predicted binding affinity of the MS peptides from mono-allelic cells (top) and multi-allelic cells (bottom) and those MS datasets mixed with random peptides, as histograms for six representative HLA alleles

The data distribution of the predicted binding affinity scores (log_10_ (IC_50_)) from mono-allelic cells and multi-allelic cells was explored. A dataset of MS peptides from multi-allelic cells expressing six alleles of HLA class I including A*03:01, A*68:01, B*07:02, B*44:02, C*07:01 and C*07:02 was analyzed. To compare with data from mono-allelic cells, MS peptides from the mono-allelic datasets for the six alleles were selected. The MS peptides from multi- and mono- allelic cells were mixed with the same set of random peptides, and MHC-peptide binding affinity for their specific HLA alleles were predicted using NetMHCpan4.1. The histogram plots were created from the predicted scores to display the data distribution of MS peptides from mono-allelic cells, multi-allelic cells and random peptides (Fig. 2). The distribution shape of the scores for MS peptides from mono-allelic cells was almost exclusively a single right skewed peak with lower log_10_ IC_50_ values (<3 or 3.5) for A and B loci, with a more left skewed peak for C alleles around log_10_ IC_50_ of 2–4. In all cases, the overlay of random peptides demonstrated a peak with log_10_ IC_50_ > 4. Since peptides identified by MS data from mono-allelic cells are highly likely to be genuine binding peptides for a specific HLA allele, we interpreted that the peak on the left with low IC_50_ values (low IC_50_ is high binding affinity) is the distribution of binding peptides (true data), whilst the right peak (high IC_50_ is low binding affinity) is the distribution of the non-binding peptides (false data). The distribution shape of MS peptide binding predicted for A*03:01, A*68:01 and B*07:01 from multi-allelic cells displayed a bimodal distribution, one located on the left and the other on the right side. This is expected, since only some of the presented peptides in multi-allelic cell lines are presented by one allele. However, the left peak of B*44:02, C*07:01 and C*07:02 can hardly be observed, most predicted scores located on the side of low binding affinity. This may be an experimental artefact or a true biological result, in that the set of peptides presented are dominated by only three alleles. The distribution shape of the low binding affinity peptides from multi-allelic cells well matches the distribution shape of random peptides, indicating that random peptides also well model the set of non-binding peptides.

The distribution of predicted scores from MS peptides from mono-allelic cells mixed with random peptides for a more complete set of 85 HLA alleles is shown in Supplementary Figure S2. In almost all cases, the MS peptide distribution is clearly separated from the random peptide distribution. While the distribution shape of random peptides appears highly similar shown as a right skewed distribution, some of the MS peptide distributions vary across different groups of alleles. Most A and B locus alleles have a slight left skewed or symmetrical distribution. The C locus alleles tend to have a symmetrical or right skewed distribution (i.e. only a few high binding affinity peptides). The shape of the data distribution is important since we wish to model distribution shapes to calculate local and global statistics from data distributions.

### 3.2 Statistical model fitting data distribution

**Fig. 3.**
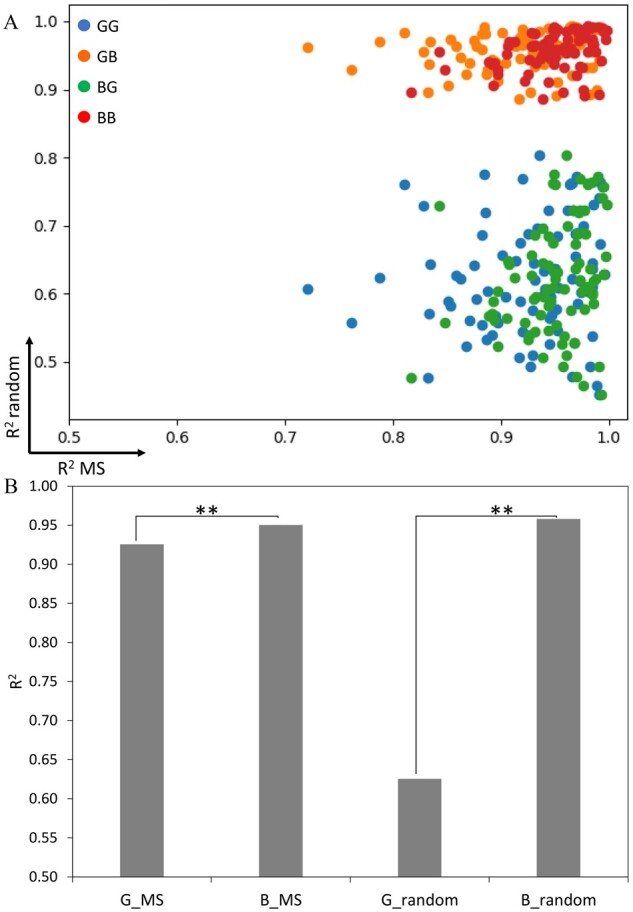
Similarity measurement of model fitting data distribution. (**A**) The scatter plots of *R*^2^ values from MS datasets (*x*-axis) and random (*y*-axis) datasets of 85 HLA alleles that were fitted by beta or Gaussian model; a mixture of Gaussian-Gaussian (GG), a mixture of Gaussian-beta (GB), a mixture of beta-Gaussian (BG), a mixture of beta-beta (BB). (**B**) The average *R*^2^ of Gaussian and beta model fitting MS and random datasets from 85 HLA alleles. Each bar represented the mean of *R*^2^ from 85 HLA alleles. (*P-value < 0.05, **P-value < 0.01); Gaussian fitting MS data (G_MS), beta fitting MS data (B_MS), Gaussian fitting random data (G_random), beta fitting random data (B_random)

To find statistical models that can properly model the observed bimodal distributions shown in Supplementary Figure S2, the evaluation of mixture models including a mixture of Gaussian-Gaussian (GG), a mixture of Gaussian-beta (GB), a mixture of beta-Gaussian (BG) and a mixture of beta-beta (BB) was performed as described in Section 2.2. The scatter plots of *R*^2^ values from 85 HLA alleles were plotted across possible mixture components of the statistical models, where the first component is the *R*^2^ of MS peptides and their simulated data, and the second component is random peptides and their simulated data. The result showed that the *R*^2^ values from GB and BB were very close to 1 for most HLA alleles (Fig. 3A). This result suggests that the true data distribution (left peak) can reasonably well be modelled by both Gaussian and beta distributions while the beta model gives the best fit for false data distribution (right peak). However, the average *R*^2^ from datasets of 85 HLA-alleles from the beta model fitting true data distribution (0.95) was significantly higher than the Gaussian model (0.93) (*P*-value = 1.77E-0.7, using paired *t*-test) (Fig. 3B). Hence, these results indicated that the beta mixture is the most suitable model to fit the predicted scores of data containing a mixture of binding and non-binding peptides, and the two-component beta model was thus used as the basis for MHCvision.

### 3.3 Estimation of beta parameters

The results in the Figure 3 indicated that the distribution of predicted MHC binding affinity from a mixture of true and false peptides is captured well by a two-component beta mixture model with the first component (beta 1) representing low IC_50_ values (*true data*) and the second component (beta 2) for high IC_50_ values (*false data*). To estimate the size of true and false data from the predicted result, the parameters of beta mixture distribution including two mixture proportions $\left(\pi_{\mathrm{true}},\;\pi_{\mathrm{false}}\right)$, $\alpha_{\mathrm{true}},\;\alpha_{\mathrm{false}}$ and $\beta_{\mathrm{true}},\;\beta_{\mathrm{false}}$ were estimated from the predicted dataset using the EM algorithm with a method of moments estimation for the beta mixtures. Since the second component of the data distribution is a set of scores of non-binding peptides, the distribution shape of any predicted scores of non-binding peptides with the same HLA allele should be similar. Therefore, the values of $\alpha_{\mathrm{false}}$ and $\beta_{\mathrm{false}}$ from different datasets of each HLA allele were explored to test for variation of beta model parameter ranges dependent on dataset size and peptide length. The calculated values of α and β from random datasets have a small variation across different data sizes for most HLA alleles (Supplementary Fig. S3), from which we infer that the calculated values of α and β can apply to any false dataset for the same specific HLA allele. The ranges of calculated values of $\alpha_{\mathrm{false}}$ and $\beta_{\mathrm{false}}$ were used to constrain the estimated $\alpha_{\mathrm{false}}$ and $\beta_{\mathrm{false}}$ in the MM-step, while the estimated $\alpha_{\mathrm{true}}$ and $\beta_{\mathrm{true}}$ values are constrained only for the case of an input data consists almost all false data (see Methods).

**Fig. 4.**
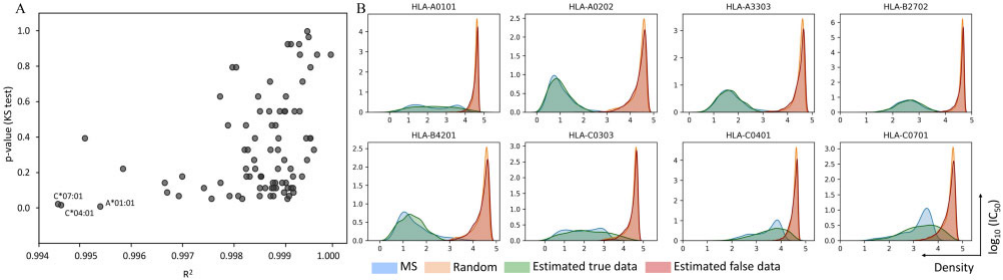
The analysis of the parameter estimation model for beta mixture testing with the predicted datasets of 9mer peptides of 85 HLA alleles. (**A**) The *R*^2^ and *P*-value from KS test between the real and simulated data. (**B**) The overlaying of density plots between the real and simulated datasets of 10 representative alleles, the overlaying distributions of 85 HLA alleles are in the Supplementary Data  S2

The datasets of predicted scores from 1000 MS and 4000 random peptides with 9mer in length for 85 HLA alleles were used to test the accuracy of the parameter estimation model. The correctness of estimation results was evaluated by similarity measurement between the real dataset (predicted IC_50_ scores) and the simulated dataset generated by the estimated parameters. The *R*^2^ between the real and simulated dataset for all 85 alleles are greater than 0.99 (Fig. 4A). However, the *R*^2^ can only describe a similarity of distribution shape but not scaling between two datasets. To ensure that simulated data can represent a given observed data, we also considered other values in the linear equation including the slope and intercept, and they are also close to 1 and 0, respectively (Supplementary Fig. S4A and B). Furthermore, the difference between two distributions of real and simulated data for 85 HLA alleles was tested by KS test. The *P*-values from KS test are higher than 0.05 for almost all alleles indicating that distributions of real and simulated data are not significantly different, although there are few alleles that have *P*-value less than 0.05, which are A*01:01, C*04:01 and C*07:01 (Fig. 4A). Data from these three alleles does not follow an expected distribution. We hypothesized that the MS data for these three alleles contains a large number of false positives, perhaps due to inadequate FDR control in the source experiment. This stage of the process is to demonstrate the overall performance of MHCVision, which, importantly, refits the beta distributions to the shape of true and false positives in a new dataset. As such, we believe these anomalies are the result of problems with the MS data we are using for evaluation, rather than with the model itself.

The overlaying of a density plot for each HLA allele between the real and simulated dataset is shown in Figure 4B. In most HLA alleles, the distribution of real and simulated data for both the left and right peaks showed a good alignment (81 of 85 alleles have *R*^2^ ≥ 0.995). The ideally fitted distributions, whose *R*^2^ > 0.999, were found in the dataset that have clear separation of MS and random peptides and a symmetrical distribution of MS data such as A*02:02, A*33:03, B*27: 02 and B35:07. Some datasets that have a flatter shape of MS peptide distribution or left skew (e.g. A*01:01, C*03:03 and B*42:01) showed imperfect matching of some bins, but the general location of the real and simulated data are the same position. However, there are four datasets that have right skew distribution of MS data such as C*04:01, and C*07:01, displayed less good alignment between real MS data and their simulated data (those alleles have *R*^2^ < 0.995). Nevertheless, the distribution of false data is well captured by their simulated data indicating that the ratio of false positive to true positive in that area of the MS data should still be correct. The overlaying density plot of all 85 HLA alleles are in the Supplementary Data  S2. The parameter estimation analysis was also performed with datasets with a larger imbalance ratio containing 1000 MS peptides and 8000 random peptides, and the result showed that the similarity between real and simulated datasets for 85 HLA alleles are close to 1 (*R*^2^ > 0.995, Supplementary Fig. S4C). The *R*^2^ from datasets with 4000 random is very similar to that from data with 8000 random indicating that MHCVision is robust for datasets with a larger imbalance between true and false data points, as may well be encountered in biological samples.

Since the common lengths of MHC I peptides are 8–11 mer, hence, the mixture of MS and random datasets with different peptide lengths were generated for more thoroughly testing the performance of the parameter estimation model. There are 16 HLA alleles, with MS peptides available for all lengths (8, 9, 10 and 11 mer), which were used to test the estimation performance of the model, 800 MS peptides (200 per length) and 3200 random peptides (800 per length). It was found that the *R*^2^ values of the real dataset and simulated dataset for 16 HLA alleles are highly close to 1 (Supplementary Fig. S5A). The value of *R*^2^ suggested that the parameter estimation model functions well for the dataset with multi-lengths of peptides, which are shown by a good alignment of density plots between the simulated dataset created by the estimate parameters and the real dataset (Supplementary Fig. S5B). Furthermore, the robustness of the estimator model was evaluated with highly imbalanced distributions (i.e. almost all true, or almost all false), where selected MS datasets and random datasets were used to test with the model separately (Supplementary Fig. S6) and the predicted IC_50_ of peptides derived from MHC I multi-allelic cells (Supplementary Fig. S7). The result of similarity measure from those datasets revealed a high similarity between the real and simulated data indicating that the model can work well with datasets that are not in our sets of data used to learn and train MHCVision and provide sensible estimated parameters for data distributions with a large imbalance between true and false data. Altogether, the results of the *R*^2^ values, KS test and the overlaying of data distributions indicate that the framework of EM algorithm with a method of moments for a beta mixture model can provide sensible estimated parameters for true and false data. The estimated true and false data of the predicted results can be further used to calculate the values of FDR and PEP for an individual predicted score.

### 3.4 The estimation of FDR and PEP for the predicted scores

**Fig. 5.**
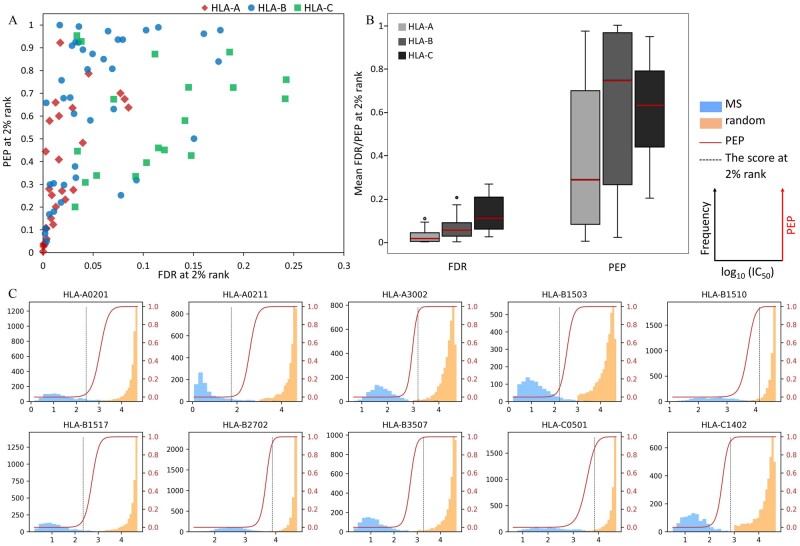
Estimation of FDR and PEP for predicted scores of 85 HLA alleles. (**A**) The scatter plot represents the values of accumulated global FDR and PEP at the 2% rank for each allele. (**B**) The distribution of FDR and PEP values at the 2% rank for HLA-A, -B and -C derived scores. (**C**) The overlaying of PEP values on the data distribution of predicted scores of 10 representative alleles, the dashed black line was marked at the score with 2% rank. Plots of 85 HLA alleles are in the Supplementary Data S3

The values of FDR and PEP of an individual predicted score were calculated from the estimated beta parameters and sizes for true and false data, following model fitting by MHCVision. From NetMHCpan’s documentation, the 2% rank is recommended to use as a hard threshold for binding peptide selection. Here, this analysis performed the estimation of statistical confidence measure of FDR and PEP for peptide binding prediction from the test datasets of a mixture of MS and random peptides. A scatter plot demonstrating FDR and PEP values at the 2% rank for 85 HLA alleles is shown in Figure 5A. The accumulated (global) FDR value at the 2% rank score of all HLA-A and most B and C loci is less than 0.1. However, ∼20% of the representative datasets (17 of 85 alleles) showed global FDR at the 2% rank are higher than 0.1 i.e. more than 10% of peptides passing the threshold are predicted to be false positives, most are found in HLA-C and a few HLA-B. To assess the confidence of each peptide’s predicted score, the PEP was computed for each peptide in the predicted dataset. The analysis demonstrated that 48 of 85 datasets, especially B and C loci, have PEP at the 2% rank over 0.5, which means the probability of each peptide being a false positive is higher than 50% even though they have < 2% rank score (Fig. 5A). At the 2% rank threshold, the FDR of HLA-C (0.13) is the highest on average followed by HLA-B (0.07), and the average of HLA-A (0.03) is the lowest. While the PEP at the 2% rank of HLA-B and HLA-C on average are greater than 0.5 (0.64 and 0.63, respectively) whilst the average PEP of HLA-A is 0.38 (Fig. 5B).

The overlaying of PEP values on the data distribution of predicted IC_50_ scores in Figure 5C showed that the log_10_ (IC_50_) < 2 or > 4 have a high certainty for being true or false binding peptides, their PEP values close to 0 and 1. For the scores in the range of 2 to 4 have less certainty to determine whether they should be true or false binding peptides, especially for less well separated datasets for some alleles. Several datasets have PEP values close to 1 for peptides with the % rank ∼ 2% e.g. A*30:02, B*15:10, B*27:02, B*35:07, C*05:01 and C*14:02, in contrast, some data have very low PEP values, even if those scores have the % rank ≥ 2% e.g. A*02:01, A*02:11, B*15:03 and B*15:17 (the overlaying of PEP scores and log_10_(IC_50_) scores of 85 HLA alleles are in the Supplementary Data S3). These results suggest that PEP values provide considerable added value over the use of the % rank for estimation of confidence in individual data points. Beyond the MS: random datasets generated for 85 alleles data, the FDRs and PEPs from data containing almost all true or all false data (Supplementary Fig. S6D) and multi-allelic data (Supplementary Fig. S7D) were calculated by MHCVision. The results demonstrate that the values of FDR and PEP correspond well with expected true and false data, giving confidence that the model will perform well when presented with genuine datasets.

### 3.5 Extensibility for MHCflurry prediction

While MHCVision has been primarily tested with NetMHCpan, past benchmarking results suggest that MHCflurry gives similar strong performance for peptide binding prediction; we thus extended our approach for predicted results coming from MHCflurry2.0. MHCflurry also reports predicted IC_50_ and % rank; however, the tool’s documentation does not suggest the cut off threshold of the % rank. Therefore, we assumed the 2% rank as a possible threshold for distinguishing binders and non-binders, as for NetMHCpan. There are 79 HLA alleles supported by MHCflurry, and there are 55 alleles of 9mer MS-random peptides in our study, which are available for those supported alleles. To estimate parameters from data distribution from MHCflurry prediction, we applied the parameter ranges that are calculated from MHCflurry predicted scores of random peptides in various data sizes (1000, 5000 and 10000) instead of parameters ranges calculated from NetMHCpan predicted scores. The *R*^2^ between the real (predicted scores) and simulated data are in range of 0.995 to 0.999 for 52 of 55 alleles (Supplementary Fig. S8A), and the overlying between the real and simulated data as shown in Supplementary Figure S9. Thus, those results indicate that the approach of EM algorithm with a method of moments also works well for predicted data coming from MHCflurry. The analysis of FDR and PEP estimation showed that if using a 2% rank threshold, over 10% global FDR occurs for 18 alleles and PEP higher than 50% for 27 of 55 alleles—indicating that as for NetMHCpan, 2% rank is not an ideal threshold for controlling FDR for many alleles. (Supplementary Fig. S8B and C).

## 4 Discussion

MHC-peptide binding affinity prediction is widely used in the immunology research and development e.g. designing immunogenic peptides for vaccine development. NetMHCpan described the predicted % rank (versus a set of random peptide scores) for each predicted IC_50_ score. However, using only the % rank might not be sufficient to quantitatively evaluate whether a predicted binding score for a peptide is a true or false positive. As we demonstrate here, local statistics i.e. the probability that a given predicted score of each peptide is a true or false positive is needed to increase confidence in binding peptide selection. In this study, we firstly studied the distribution of predicted IC_50_ scores coming from NetMHCpan4.1. The distribution of predicted scores of MS peptides from multi-allelic cells to a specific HLA displayed a bimodal distribution, containing two separated peaks, which the left and right peaks represent binding and non-binding peptides, respectively. Gaussian distributions are expected to fit well to symmetrical distributions with theoretically infinites tails. Beta distributions can fit to skewed distributions, and distributions with theoretical maxima. Given that we observe some skew to data distributions, and the distribution has a theoretical maximum at 4.7 log_10_ (IC_50_) scale (log_10_(50 000)), where 50 000 is the maximum value provided by NetMHCpan, and beta distributions well model absolute limits, it is not therefore surprising that beta distribution give superior fits for MHC I predicted scores than Gaussian distributions. Moreover, the usage of beta model fitting MHC I predicted scores is agreed by the study of Zeng’s group that they used beta distribution to model the data distribution of MHC-peptide binding affinity for MHC class I (Zeng and Gifford, 2019). To assess the practicality of the parameter estimation model, linear regression was used to model the correlation between the real and the simulated data that is generated by the best estimate parameters. The values of *R*^2^, slope and intercept were generally close to 1, 1 and 0, respectively, indicating a good similarity of the input and simulated dataset and confirming that our parameter estimation model can provide sensible parameters from an input data modelled by a beta mixture distribution. The study of parameter estimation using the EM algorithm with a method of moments for beta mixtures model has been previously reported for the application in the field of molecular biology (Schröder and Rahmann, 2017). For our work, we modified the parameter estimation model in the MM-step to restrict the estimated values of false data, thus, the application of this model is feasible for MHC supported in NetMHCpan4.1, which cover for 2915 alleles for HLA-A, -B and -C (Reynisson *et al.*, 2020).

The estimated parameters for true and false data allow the calculation of FDR and PEP for a given peptide’s predicted score in the dataset. The global FDR can describe the error rate that accumulates in the selected binding peptides from the prediction across the whole dataset, while PEP values can describe a local false probability of an individual peptide in the dataset. We demonstrated that some datasets might get over 10% FDR when using the 2% rank as a threshold, which might be too high risk to control false positives. In practice, the FDR observed is dependent upon the allele selected, as well as the actual (unknown) count of true positives in the data, relative to false positives. Moreover, there is variability in PEP values close to the 2% rank threshold—in some datasets the predicted scores at ≤ 2% rank can have PEP values very close to 1, but in other datasets the predicted scores ≥ 2% rank have a PEP less than 0.1. Furthermore, the analysis of predicted results for 55 alleles coming from MHCflurry discovered similar trends as for NetMHCpan. This finding indicates that using only the % rank for thresholding might wrongly accept false binding peptides or miss some true binding peptides in different cases, which cannot normally be differentiated straightforwardly. In our work, we applied the model of the EM algorithm with a method of moments for beta mixture distribution to the predicted scores from NetMHCpan4.1, alternatively users can opt to run with MHCflurry, but the supported alleles are limited to 79. The output will return the statistical values including FDR and true probability (1-PEP) for every predicted peptide in a dataset for a specific HLA allele. For different downstream uses of peptide binding data, rather using solely the fix threshold as the % rank to classify or prioritize binding peptides, we would recommend users of MHCVision should apply the estimation to their data from NetMHCpan or MHCflurry and decide on the most appropriate threshold, depending on their downstream use of the data. For some applications, controlling for global FDR to a low value, say 5% or 10% might be appropriate where having as many true positives as possible, with an acceptably low number of false positives is desired. Other users of the data might wish to eliminate all peptides that are unlikely to be true, and thus using PEP < 1% would ensure only highly confident peptides are taken forward. When a global threshold such as FDR < 10% is applied, most of the peptides at the bottom of the ranked list have very high PEP values, and thus themselves are unlikely to be true binders.

In summary, our study can successfully perform the estimation of statistical values including FDR and PEP for the predicted result of MHC-peptide binding prediction. The current version is suitable for the predicted result from NetMHCpan4.1 and MHCflurry. We expect that the model will work well for other MHC class I predictors that produce a bimodal distribution, with minor adaptations and testing. We have not yet assessed the extent to which the model will work for MHC class II prediction algorithms, and this is an area for future development of the tool.

## Supplementary Material

btab479_Supplementary_Data
