# Supplementary material for: MHCVision: estimation of global and local false discovery rate for MHC class I peptide binding prediction
Source: Bioinformatics. 2021 Jul 1;37(21):3830–8. doi: 10.1093/bioinformatics/btab479 (PMC8570816; doi:10.1093/bioinformatics/btab479)
Supplement: btab479_Supplementary_Data [file btab479_supplementary_data.zip › supplementary_data1_210622.pdf]

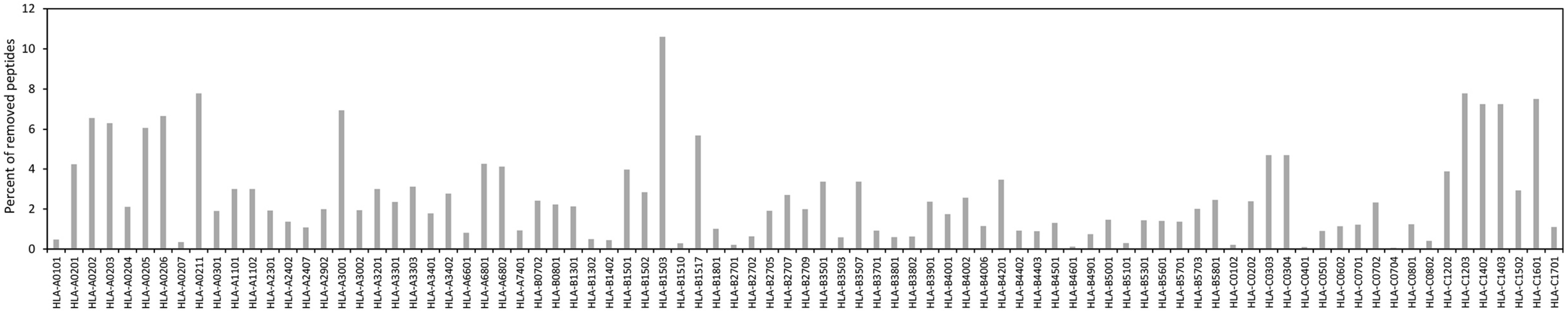

Figure S1 The percentage of removed data points (predicted IC_{50} < 1000 nM) from random peptides of 85 HLA alleles.

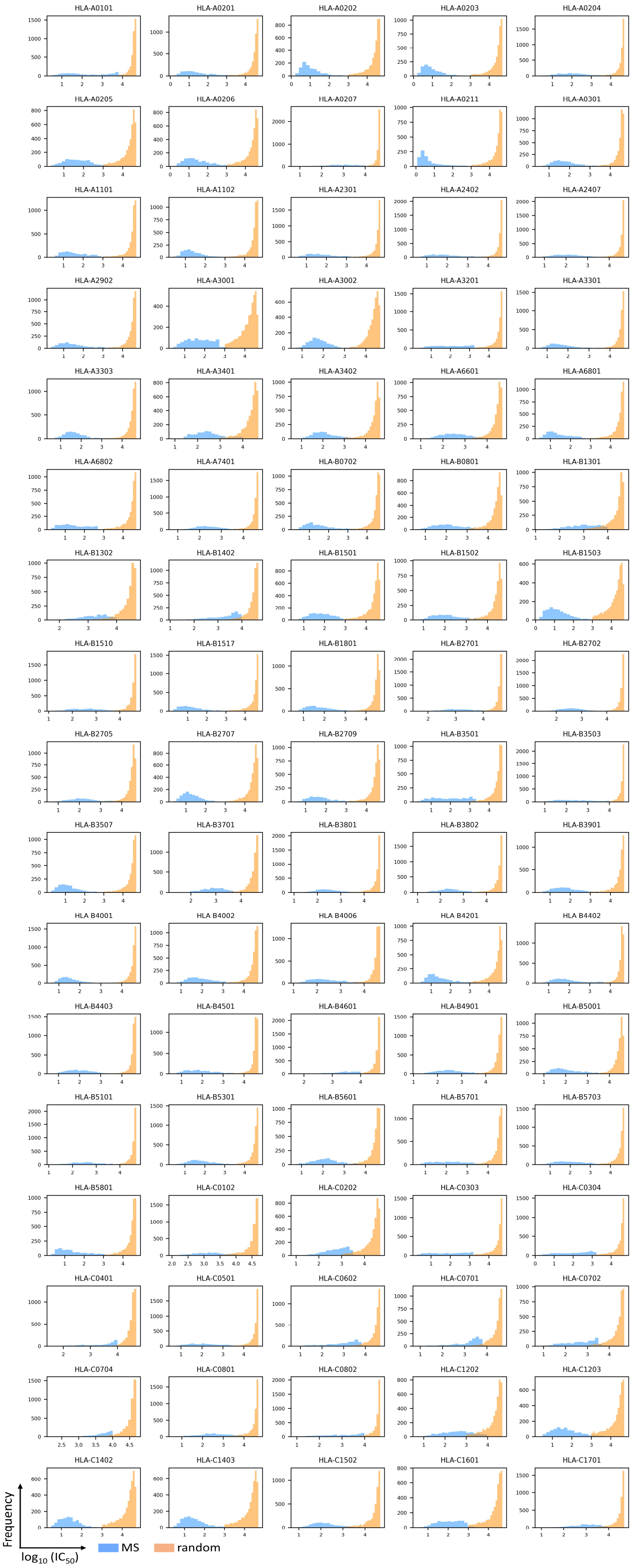

Figure S2 Histograms of the predicted scores (binding affinity in $\log_{10}$ (IC_{50}) of MS and random peptides (9mers) for 85 HLA alleles.

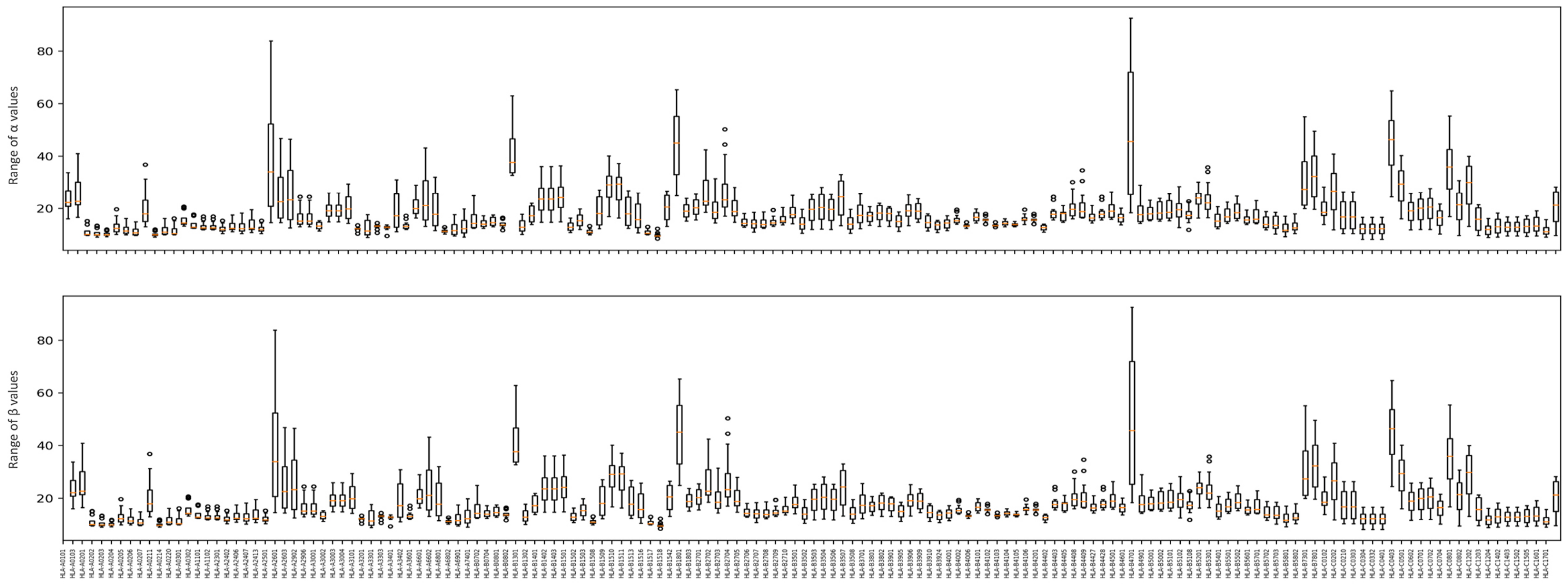

Figure S3 The box plots of calculated parameter shapes (α and β) of beta distribution from various sizes (1000, 5000, and 10,000 peptides) and lengths (8 to 11 mers) from the data sets of predicted binding scores of random peptides against 85 HLA alleles.

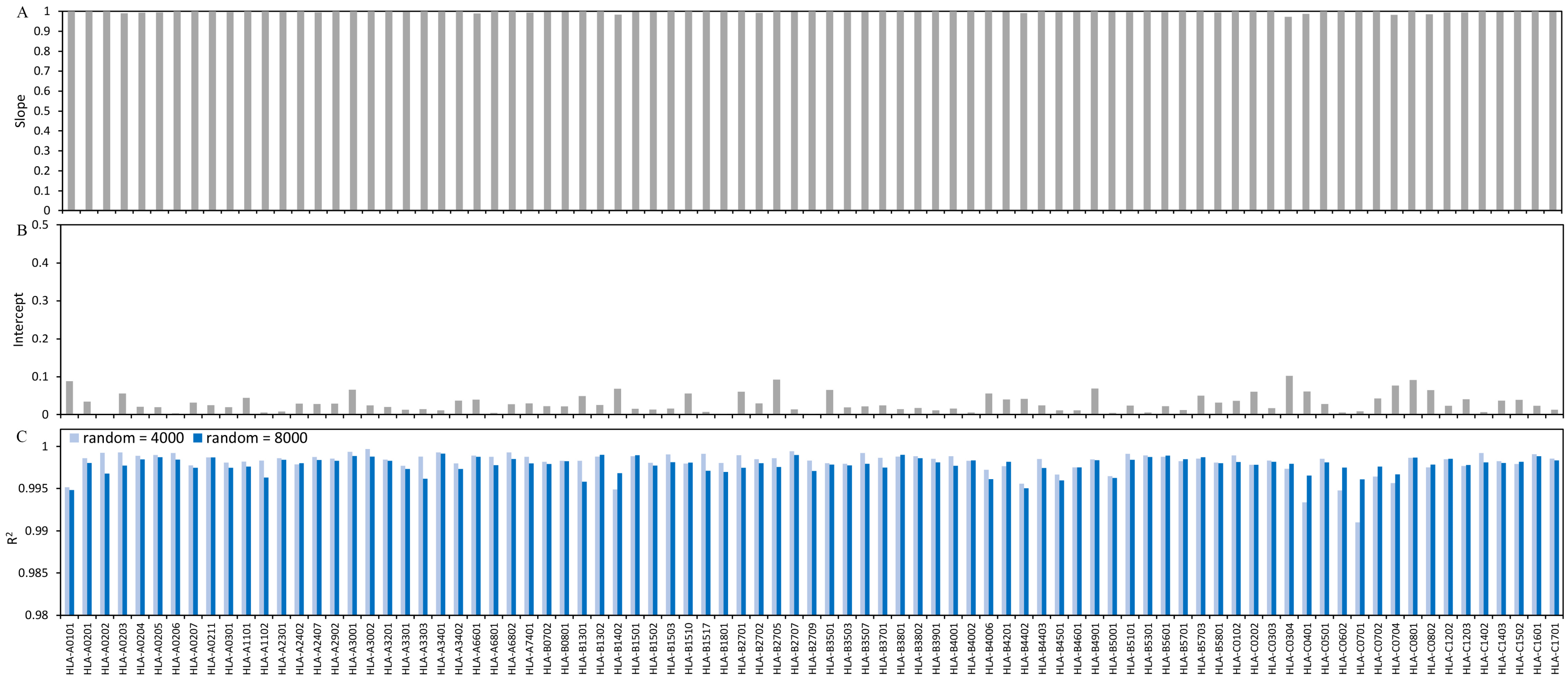

Figure S4 The slope (A) and intercept (B) values from the linear regression model fitting correlation of the real and simulated data set of 85 HLA alleles (MS:random = 1000:4000). (C) The R^2 between real and simulated data sets from data with 4000 and 8000 random peptides.

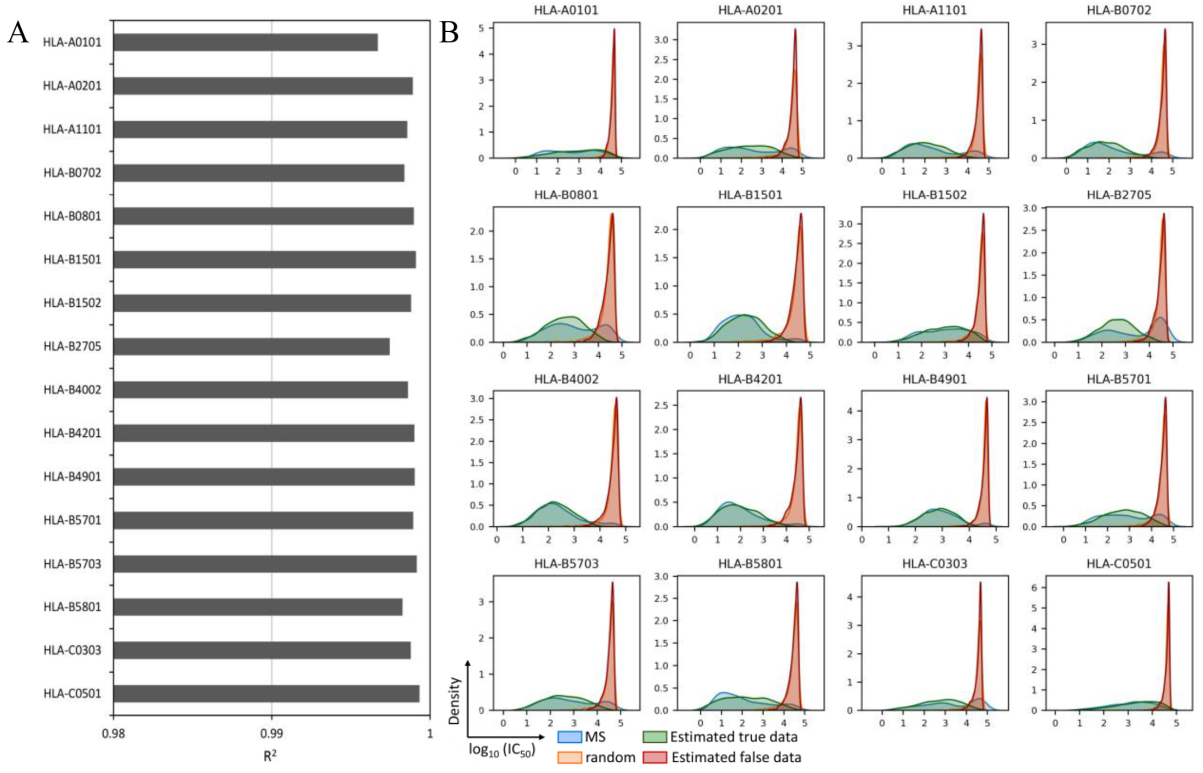

Figure S5 The estimation performance of parameter estimation model for beta mixture testing with data sets with multi-lengths peptides (8-11 mers). (A) The R^2 between the real and the simulated data set. (B) The overlaying of density plots between the real and simulated data sets.

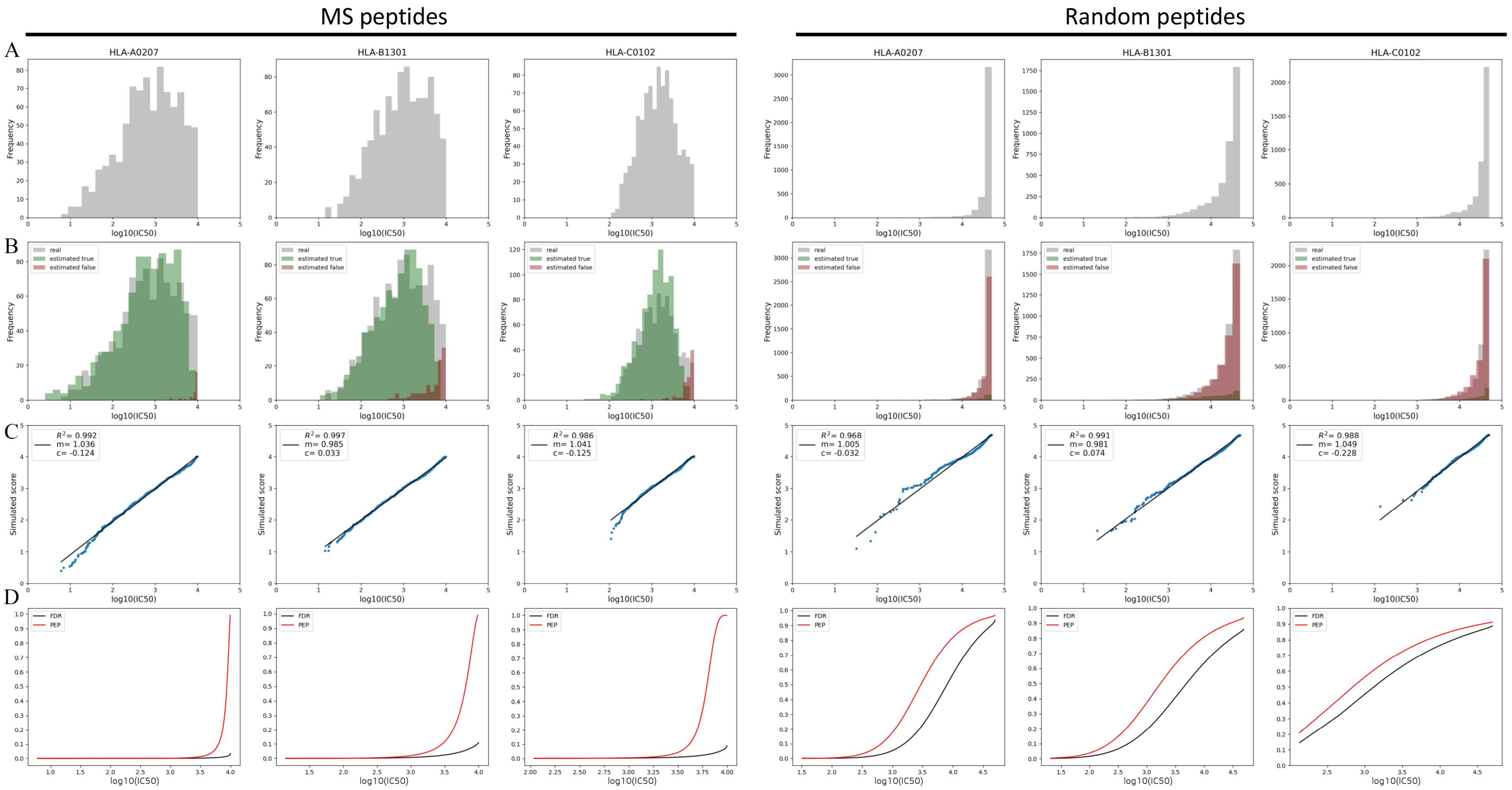

Figure S6 The estimation results from the beta mixture model on data set with a very large imbalance ratios between two components. (A) The data distribution of predicted binding affinity ($\log_{10}(IC_{50})$) of a specific HLA allele. (B) The beta models can fit to two components of data distribution to estimate parameters for true and false data. (C) The similarity measure from the linear regression model fitting correlation of the real and simulated data set, m = slope, c = y-intercept. (D) FDRs and PEPs calculated from estimated true and false data of each predicted IC_{50}.

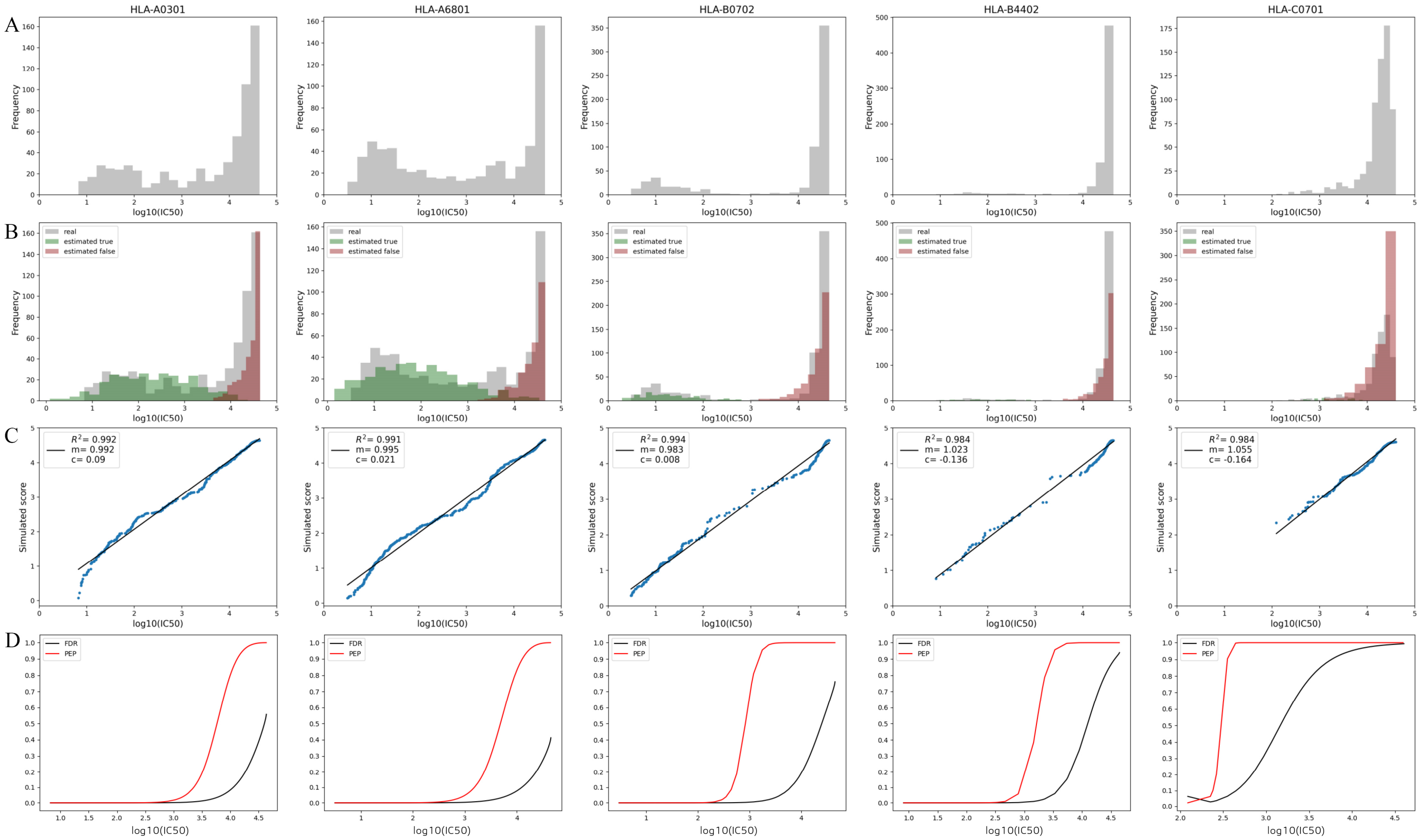

Figure S7 The estimation results from the beta mixture model on predicted IC_{50} of peptides from multi-allelic cells. (A) The data distribution of predicted binding affinity ($\log_{10}(IC_{50})$) of each HLA allele expressed by multi-allelic cells. (B) The beta models can fit to two components of the data distribution to estimate parameters for true and false data. (C) The similarity measure from the linear regression model fitting correlation of the real and simulated data set, m = slope, c = y-intercept. (D) FDRs and PEPs calculated from estimated true and false data of each predicted IC50.

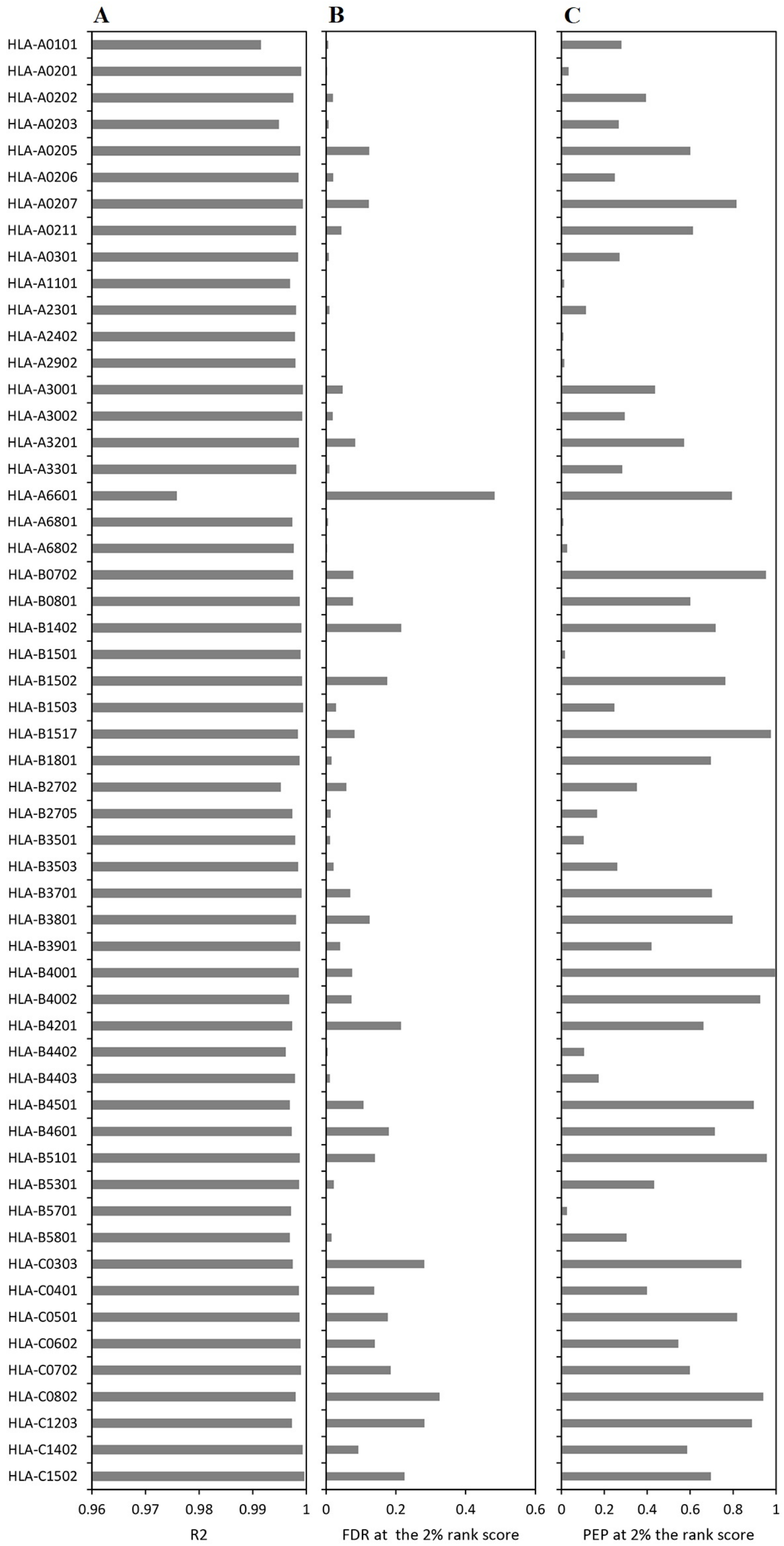

Figure S8 The analysis of the model with predicted results from MHCflurry. (A) The R^2 between the real and simulated data set. The values of accumulated global FDR (B) and PEP (C) at the 2% rank for 85 HLA alleles.

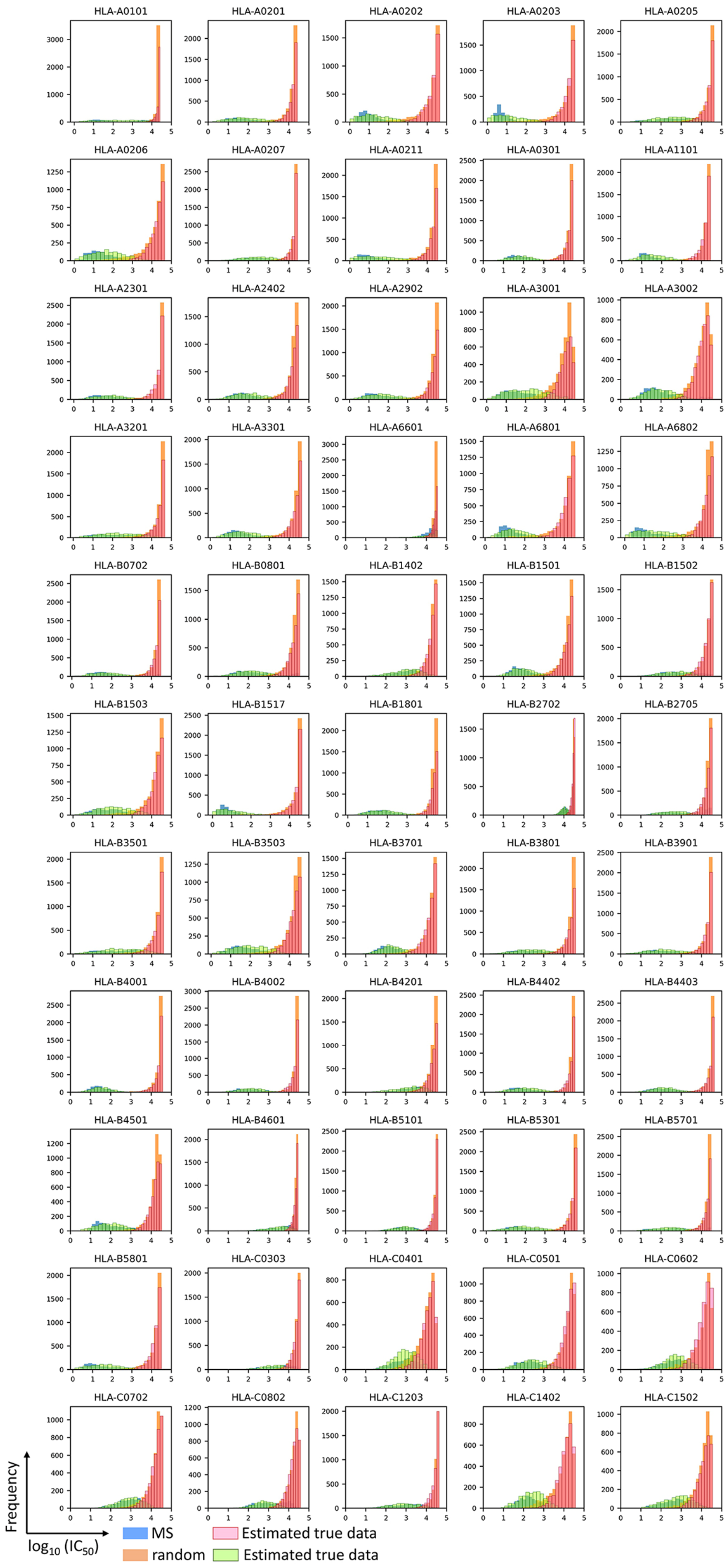

Figure S9 The overlaying of data distribution between the predicted scores from MHCflurry and their simulated data set.
