# Supplementary material for: MHCVision: estimation of global and local false discovery rate for MHC class I peptide binding prediction
Source: Bioinformatics. 2021 Jul 1;37(21):3830–8. doi: 10.1093/bioinformatics/btab479 (PMC8570816; doi:10.1093/bioinformatics/btab479)
Supplement: btab479_Supplementary_Data [file btab479_supplementary_data.zip › supplementary_data2.pdf]

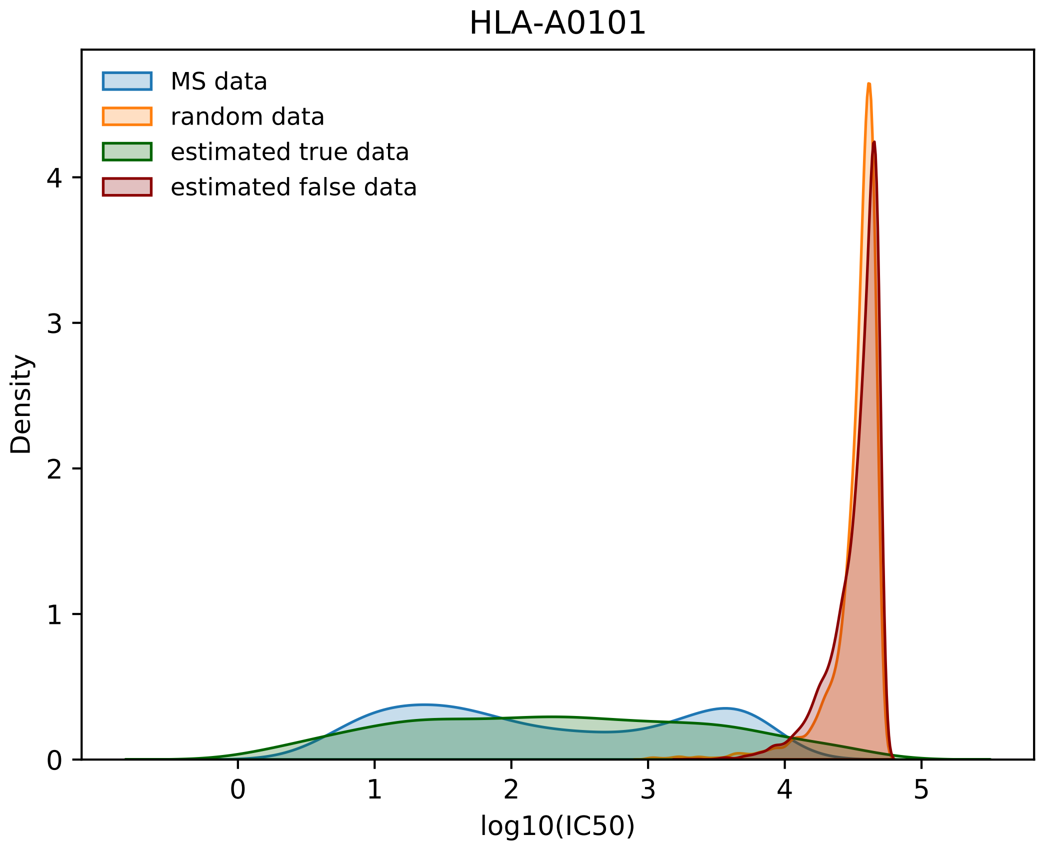
HLA-A0101
MS data
random data
estimated true data
estimated false data
Density
0
1
2
3
4
0
1
2
3
4
5
log10(IC50)

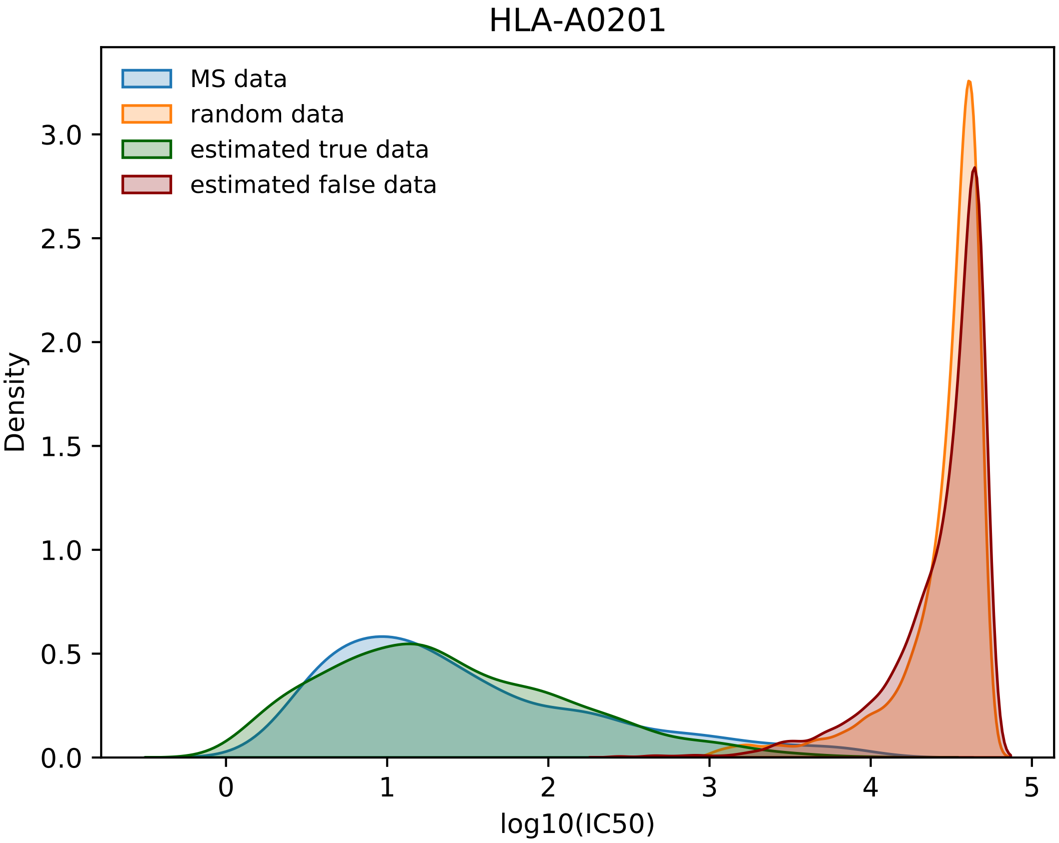
HLA-A0201
MS data
random data
estimated true data
estimated false data
Density
3.0
2.5
2.0
1.5
1.0
0.5
0.0
0
1
2
3
4
5
log10(IC50)

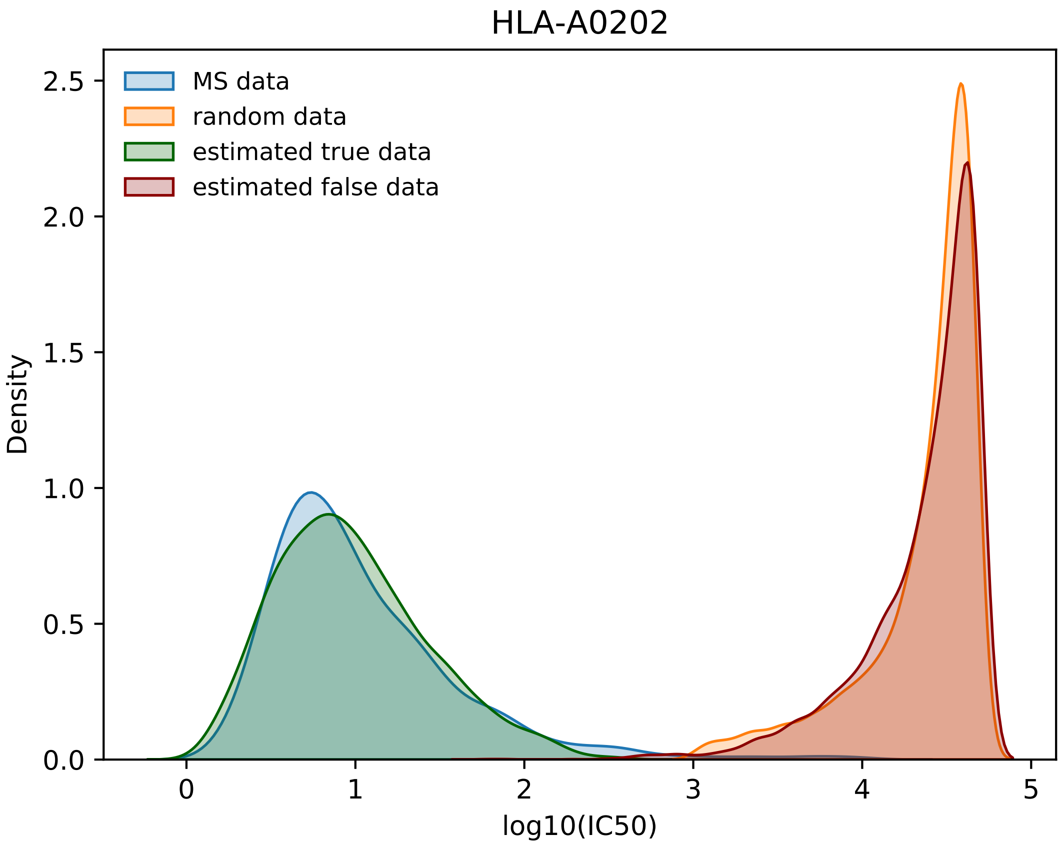

HLA-A0202
MS data
random data
estimated true data
estimated false data
Density
log10(IC50)
0.0
0.5
1.0
1.5
2.0
2.5
0
1
2
3
4
5

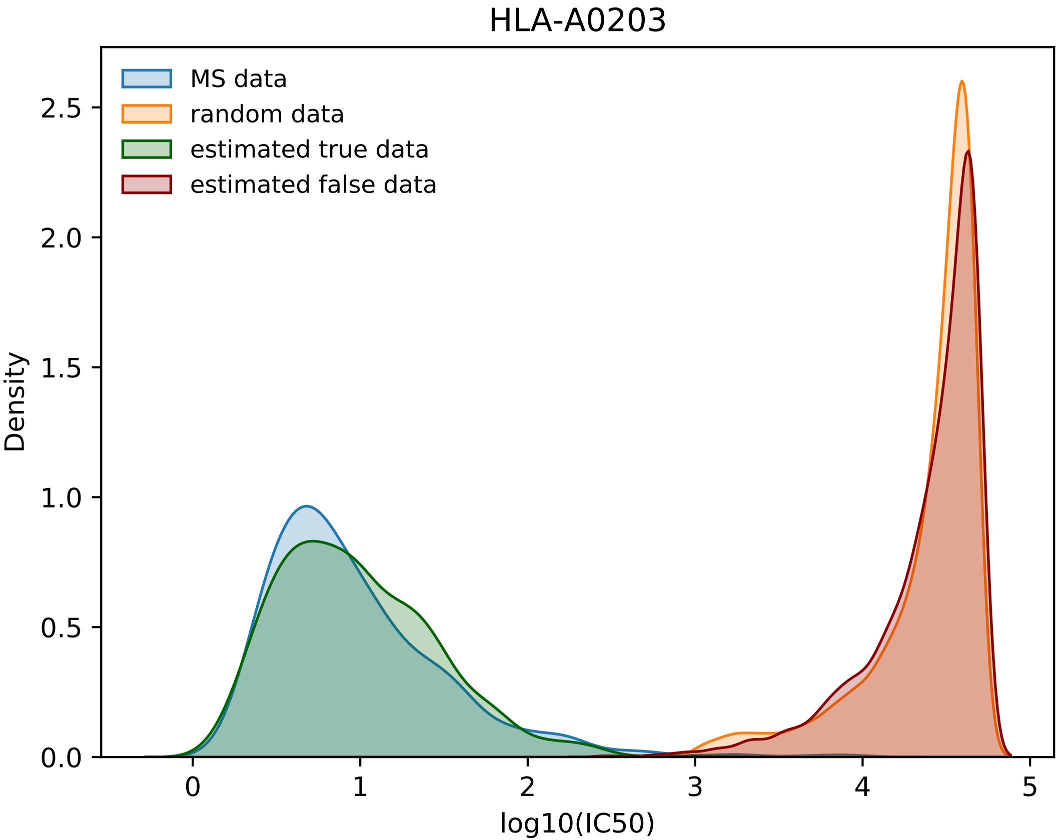

HLA-A0203
MS data
random data
estimated true data
estimated false data
Density
0.0
0.5
1.0
1.5
2.0
2.5
0
1
2
3
4
5
log10(IC50)

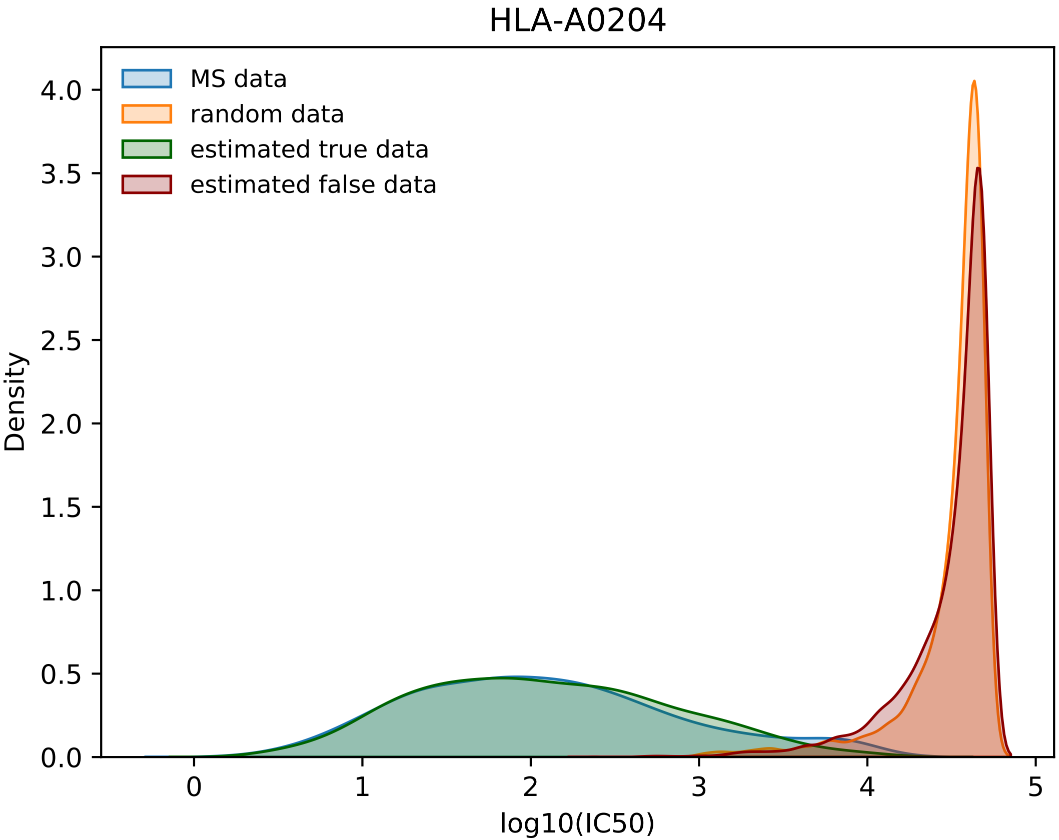

HLA-A0204
MS data
random data
estimated true data
estimated false data
Density
0.0
0.5
1.0
1.5
2.0
2.5
3.0
3.5
4.0
0
1
2
3
4
5
log10(IC50)

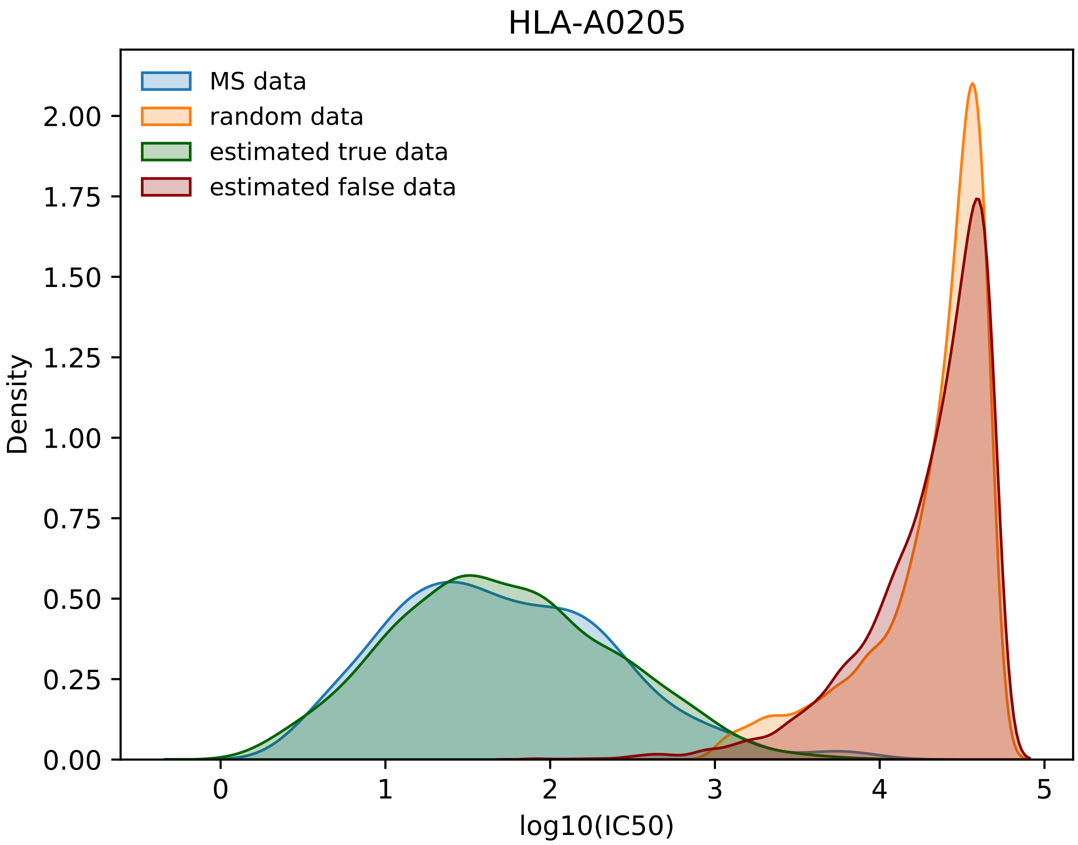

HLA-A0205
MS data
random data
estimated true data
estimated false data
Density
2.00
1.75
1.50
1.25
1.00
0.75
0.50
0.25
0.00
0
1
2
3
4
5
log10(IC50)

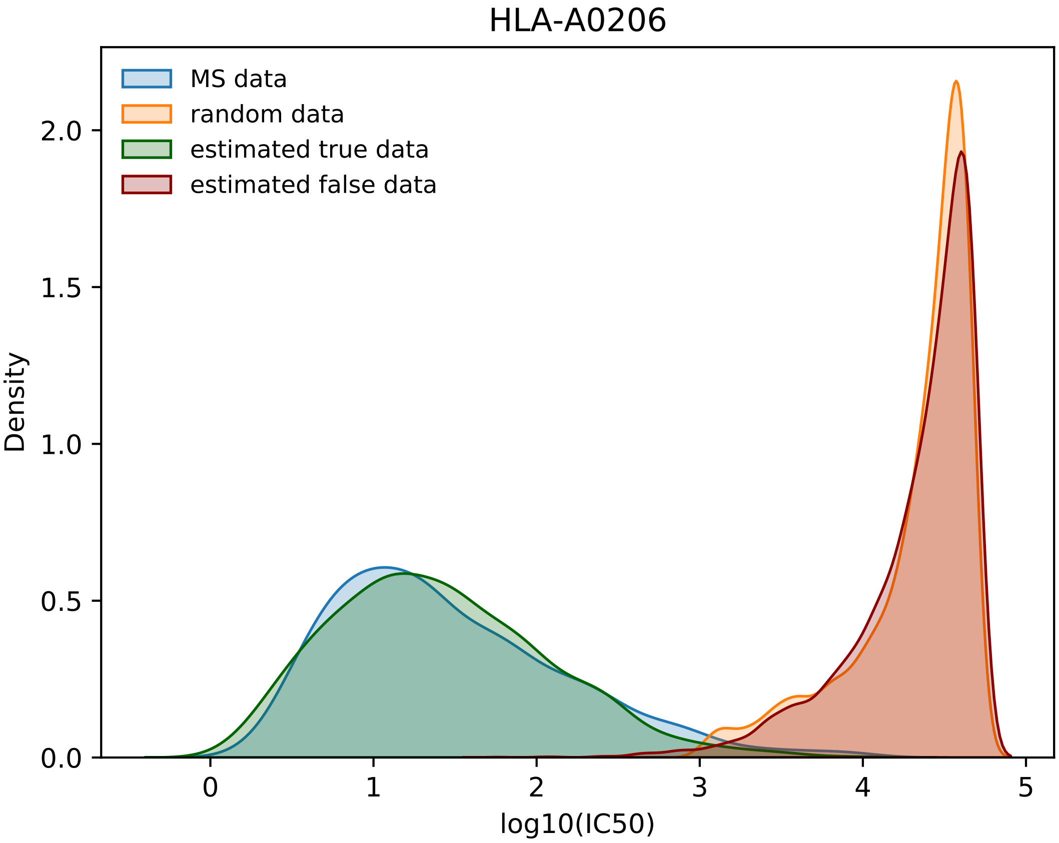

HLA-A0206
MS data
random data
estimated true data
estimated false data
Density
2.0
1.5
1.0
0.5
0.0
0
1
2
3
4
5
log10(IC50)

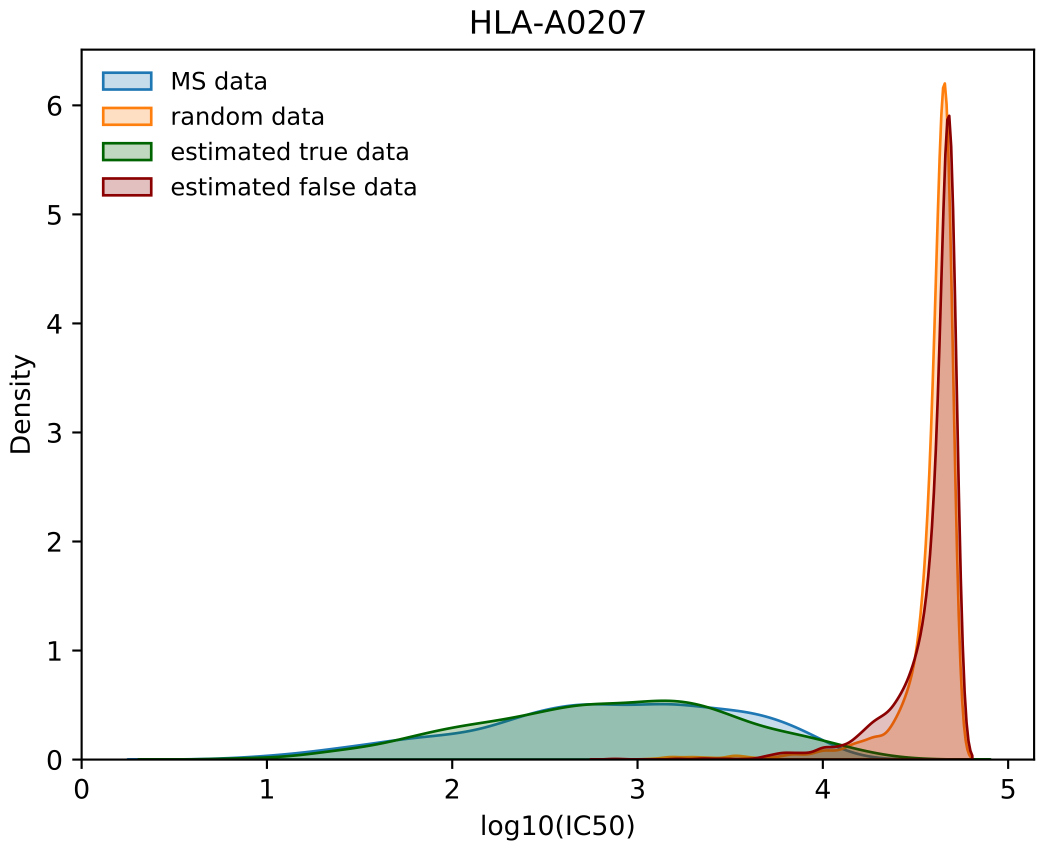

HLA-A0207
MS data
random data
estimated true data
estimated false data
Density
log10(IC50)
0
1
2
3
4
5
6

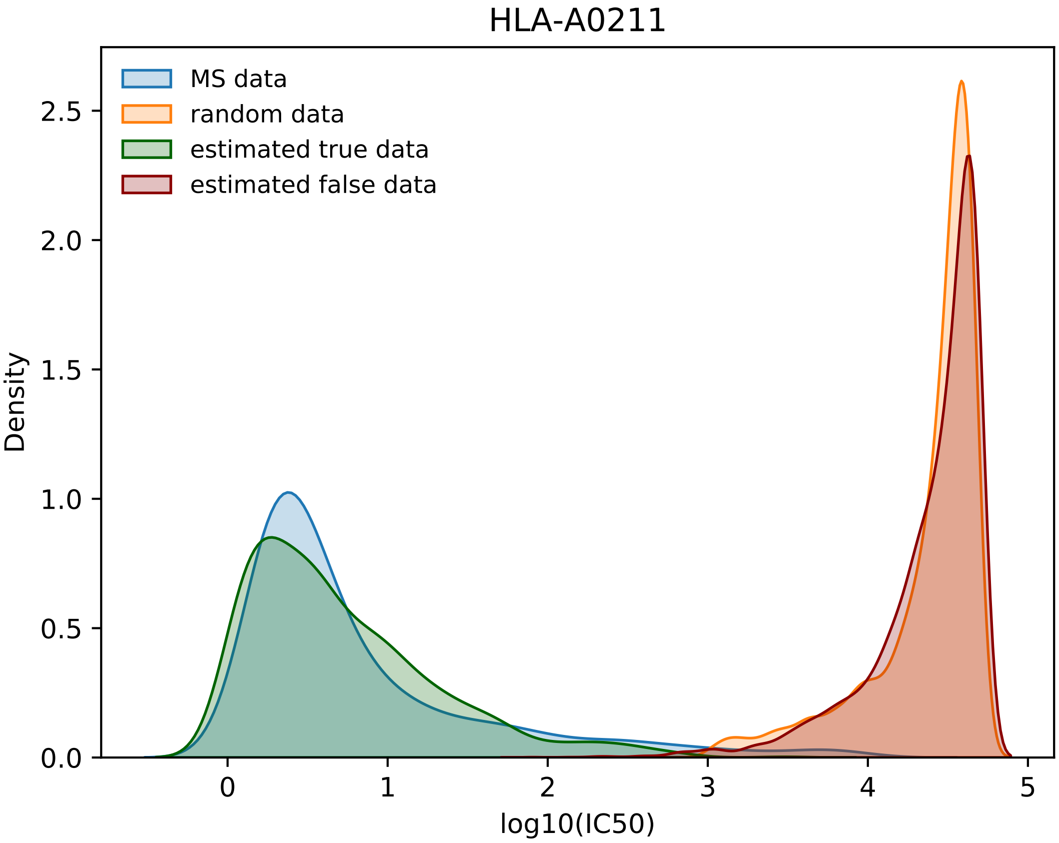

HLA-A0211
MS data
random data
estimated true data
estimated false data
Density
2.5
2.0
1.5
1.0
0.5
0.0
0
1
2
3
4
5
log10(IC50)

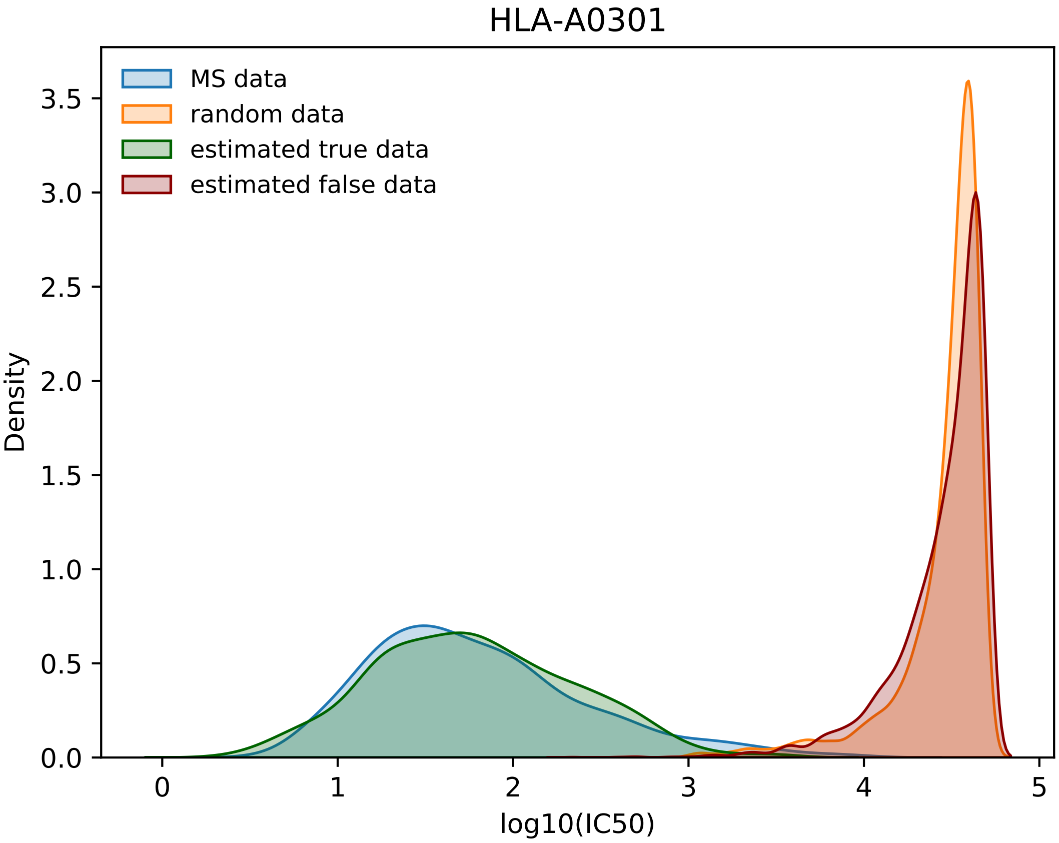

HLA-A0301
MS data
random data
estimated true data
estimated false data
Density
3.5
3.0
2.5
2.0
1.5
1.0
0.5
0.0
0
1
2
3
4
5
log10(IC50)

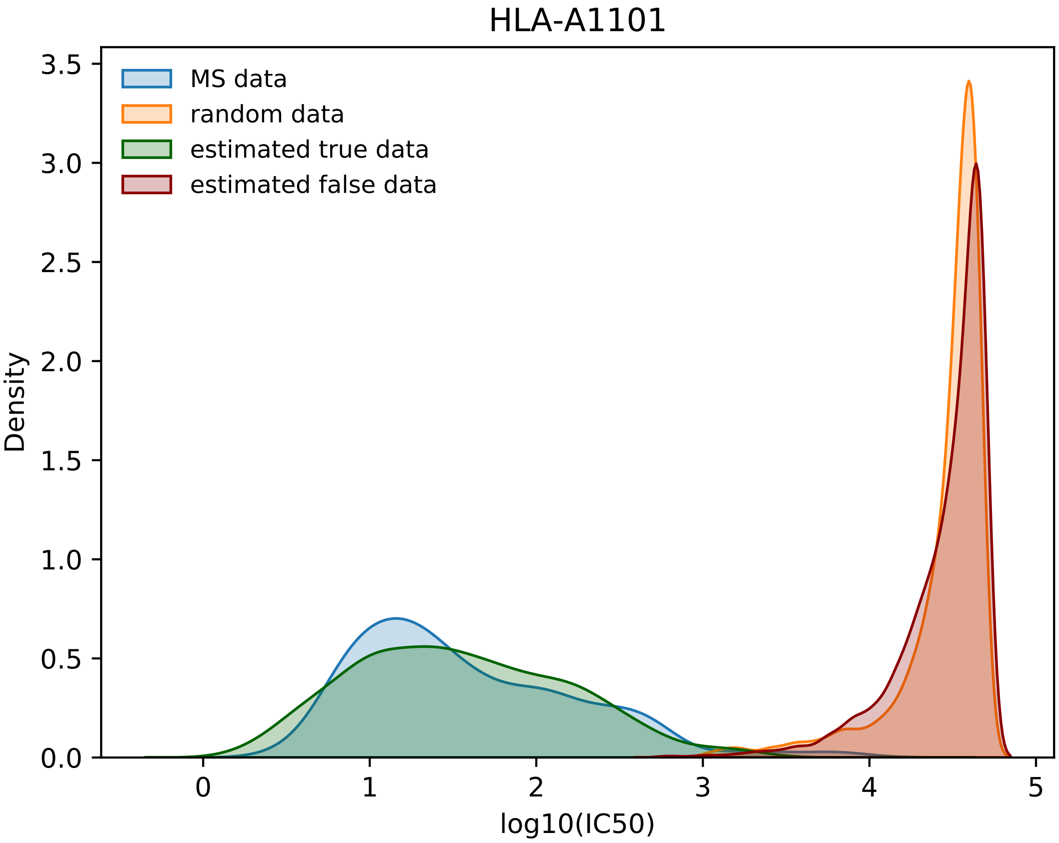

HLA-A1101
MS data
random data
estimated true data
estimated false data
Density
log10(IC50)
0.0
0.5
1.0
1.5
2.0
2.5
3.0
3.5
0
1
2
3
4
5

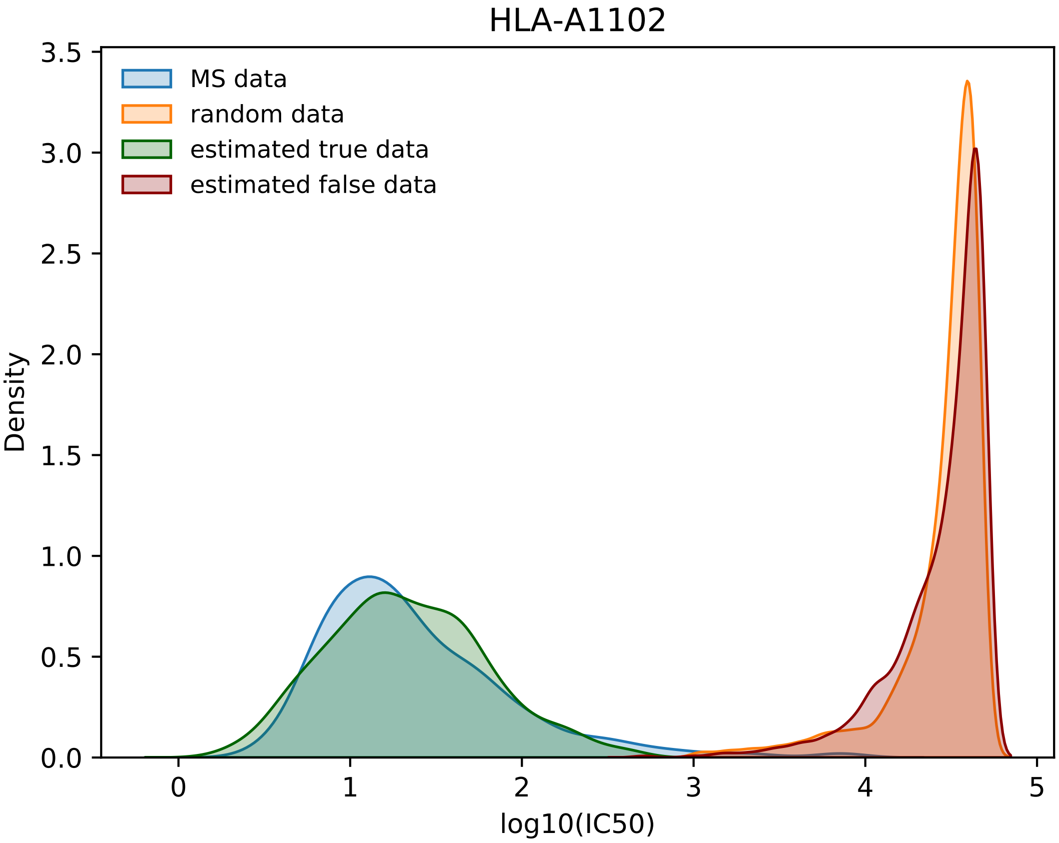
HLA-A1102
MS data
random data
estimated true data
estimated false data
Density
log10(IC50)
0.0
0.5
1.0
1.5
2.0
2.5
3.0
3.5
0
1
2
3
4
5

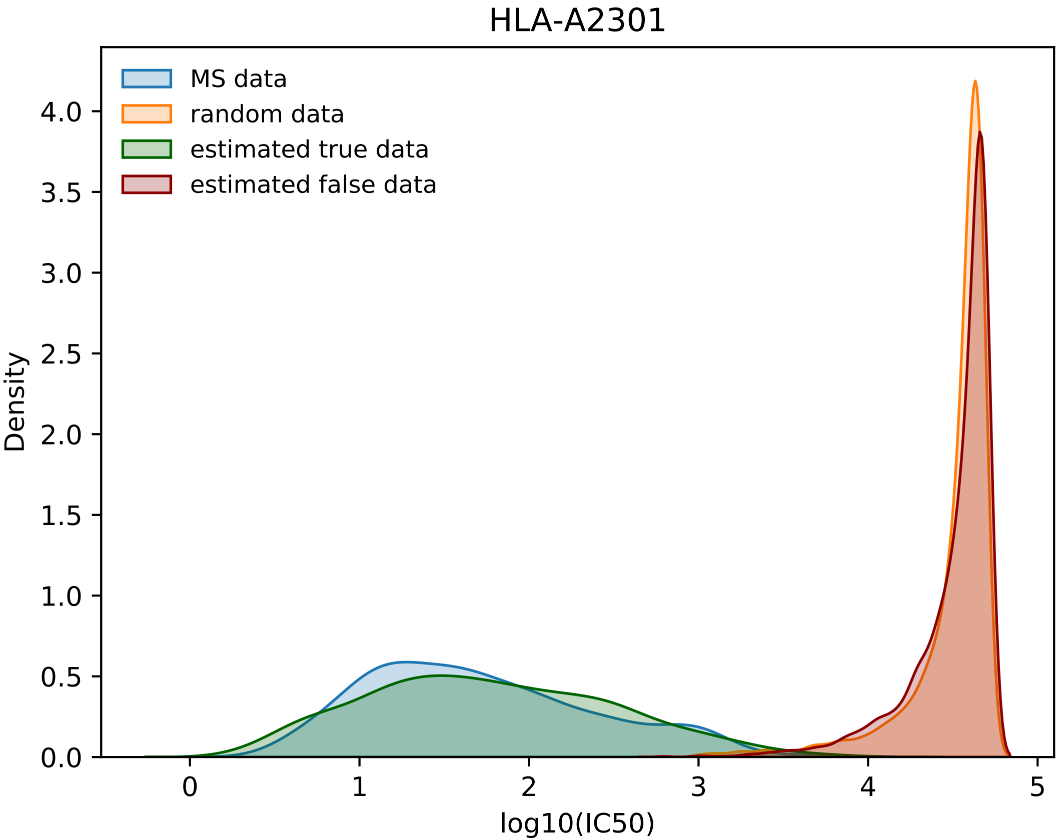
HLA-A2301
MS data
random data
estimated true data
estimated false data
Density
log10(IC50)
0.0
0.5
1.0
1.5
2.0
2.5
3.0
3.5
4.0
0
1
2
3
4
5

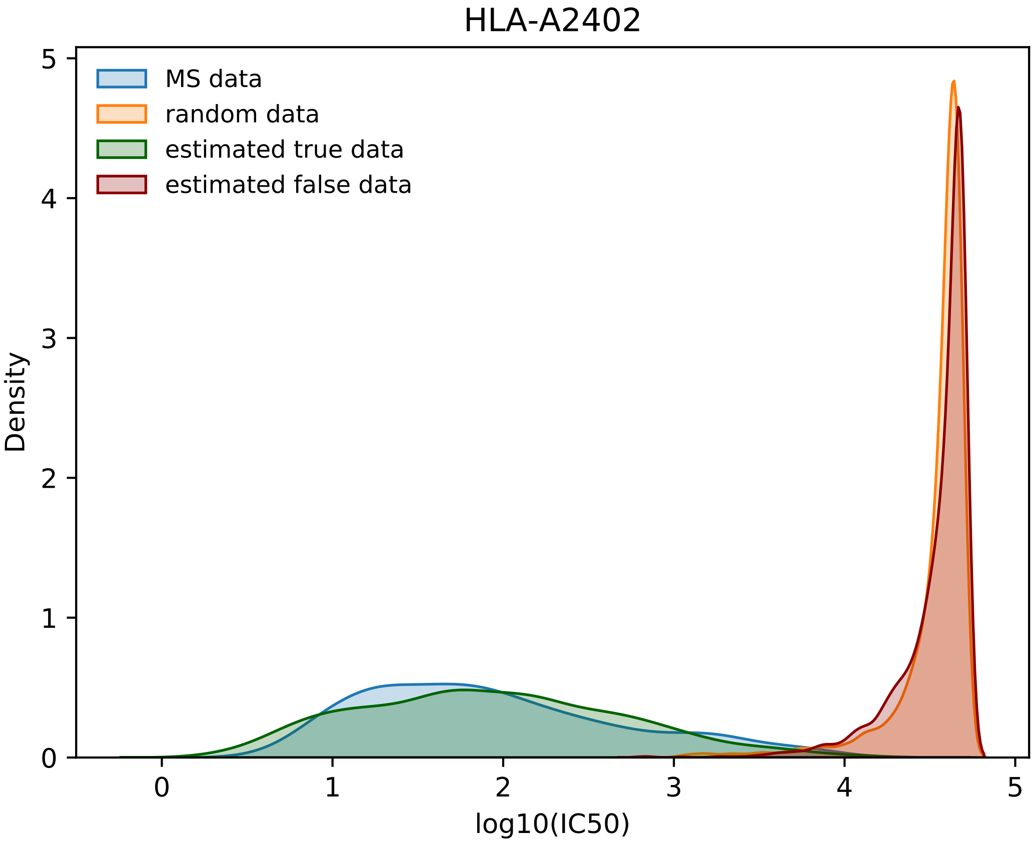

HLA-A2402
MS data
random data
estimated true data
estimated false data
Density
log10(IC50)
0
1
2
3
4
5

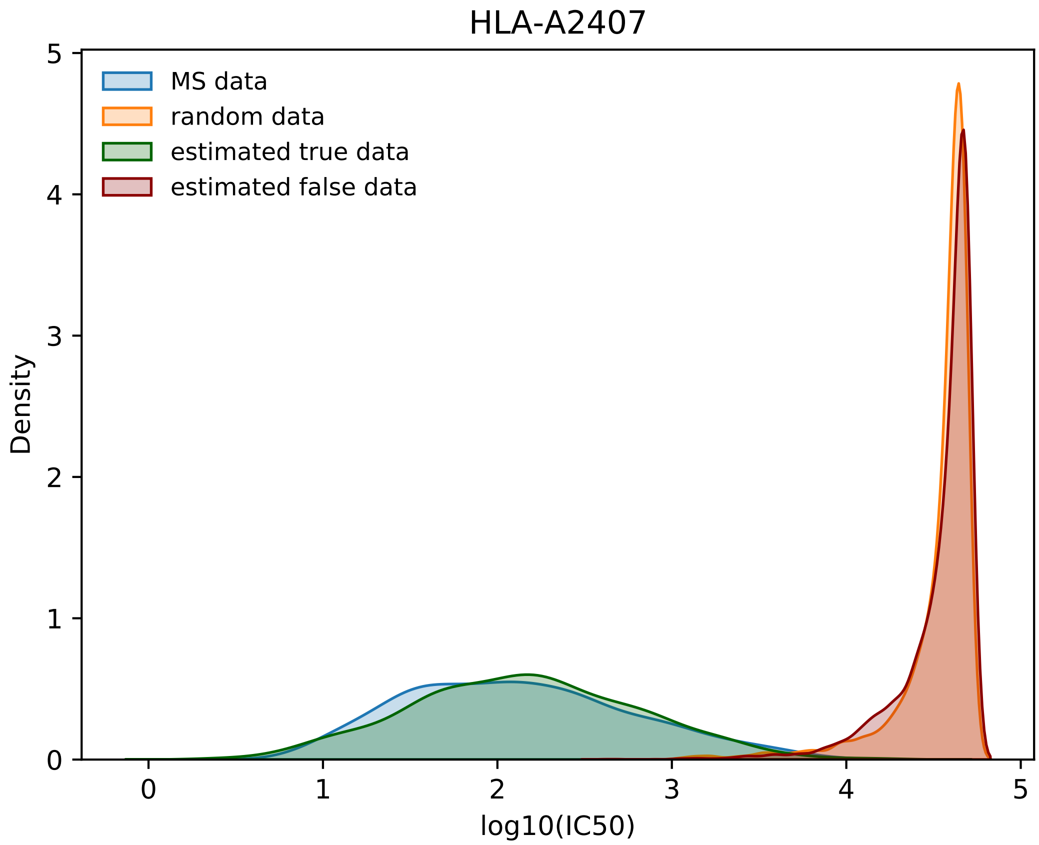
HLA-A2407
MS data
random data
estimated true data
estimated false data
Density
log10(IC50)
0
1
2
3
4
5
0
1
2
3
4
5

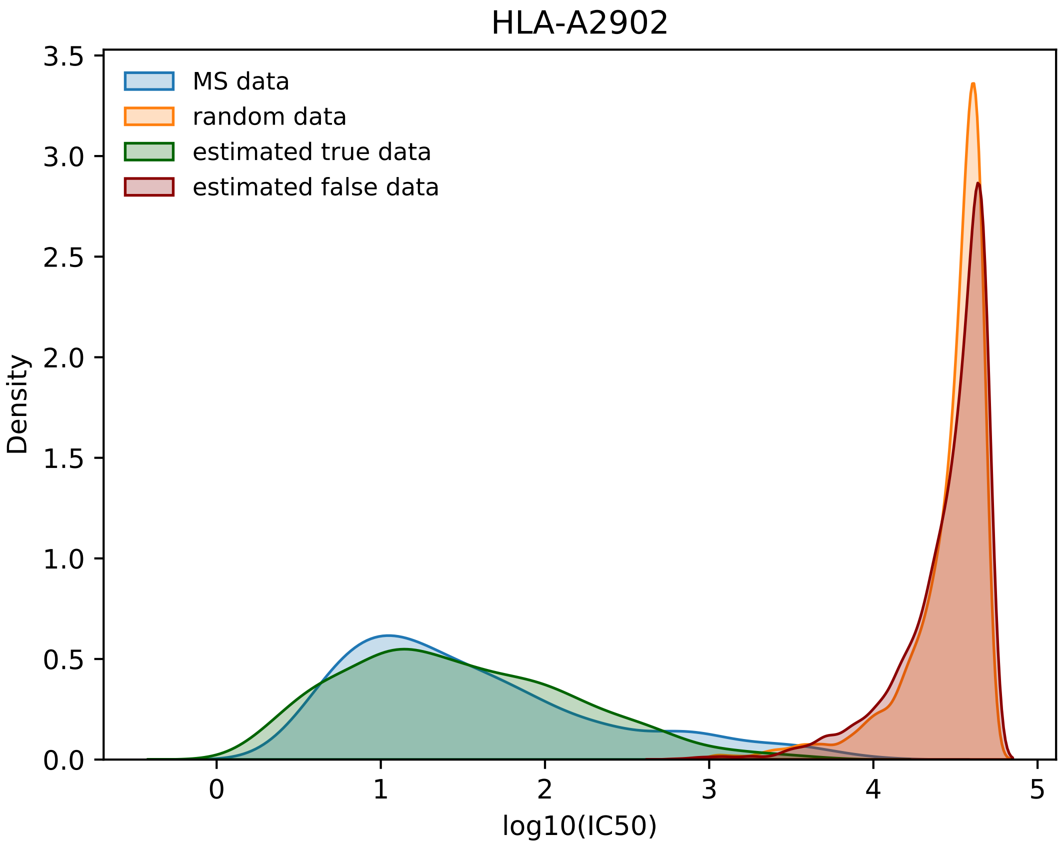
HLA-A2902
MS data
random data
estimated true data
estimated false data
Density
0.0
0.5
1.0
1.5
2.0
2.5
3.0
3.5
0
1
2
3
4
5
log10(IC50)

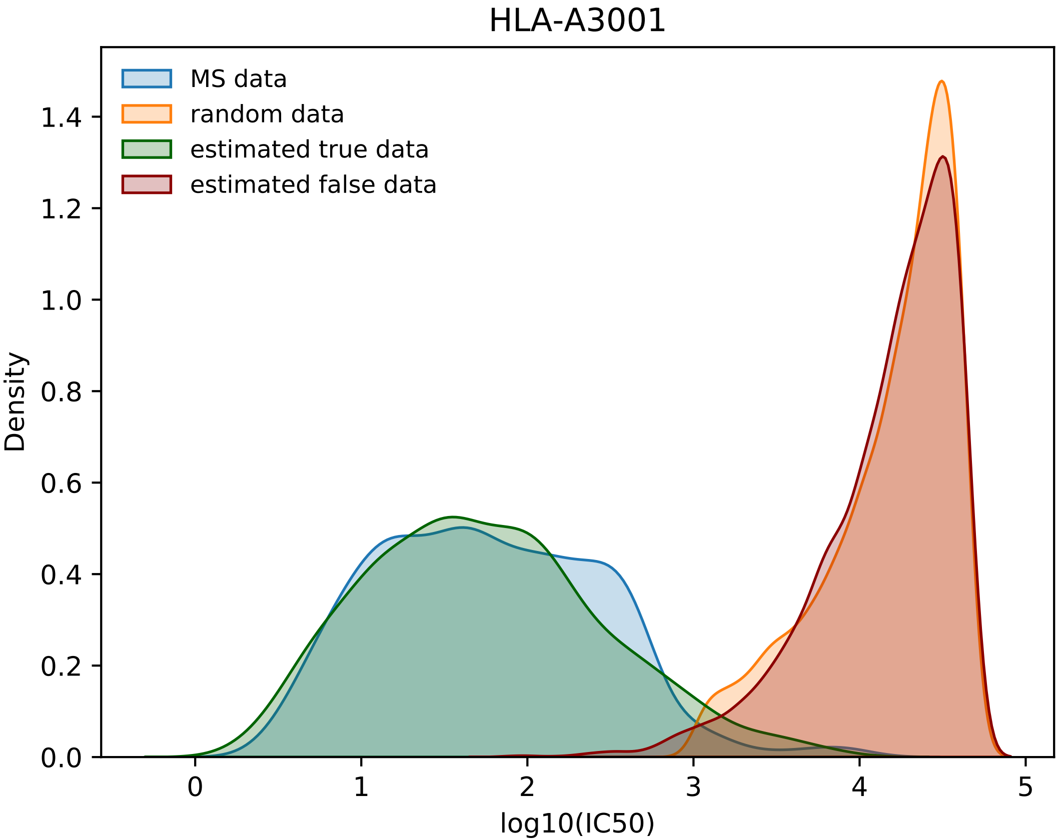
HLA-A3001
MS data
random data
estimated true data
estimated false data
Density
1.4
1.2
1.0
0.8
0.6
0.4
0.2
0.0
0
1
2
3
4
5
log10(IC50)

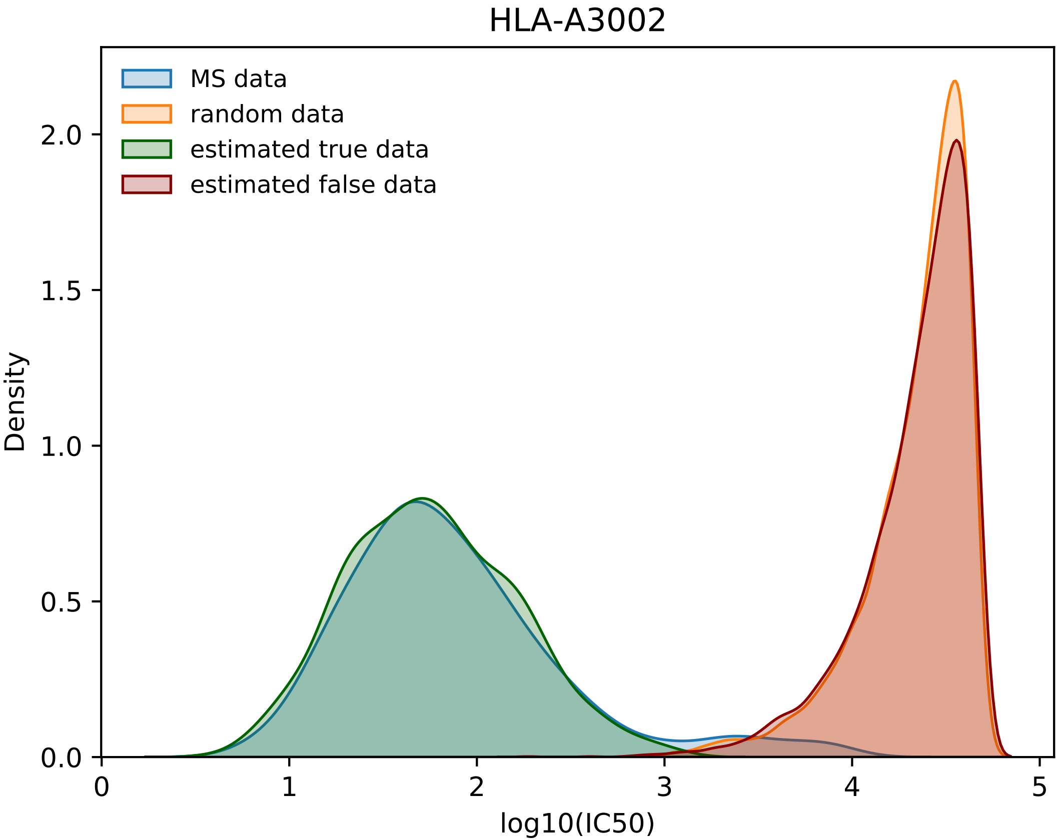
HLA-A3002
MS data
random data
estimated true data
estimated false data
Density
2.0
1.5
1.0
0.5
0.0
0
1
2
3
4
5
log10(IC50)

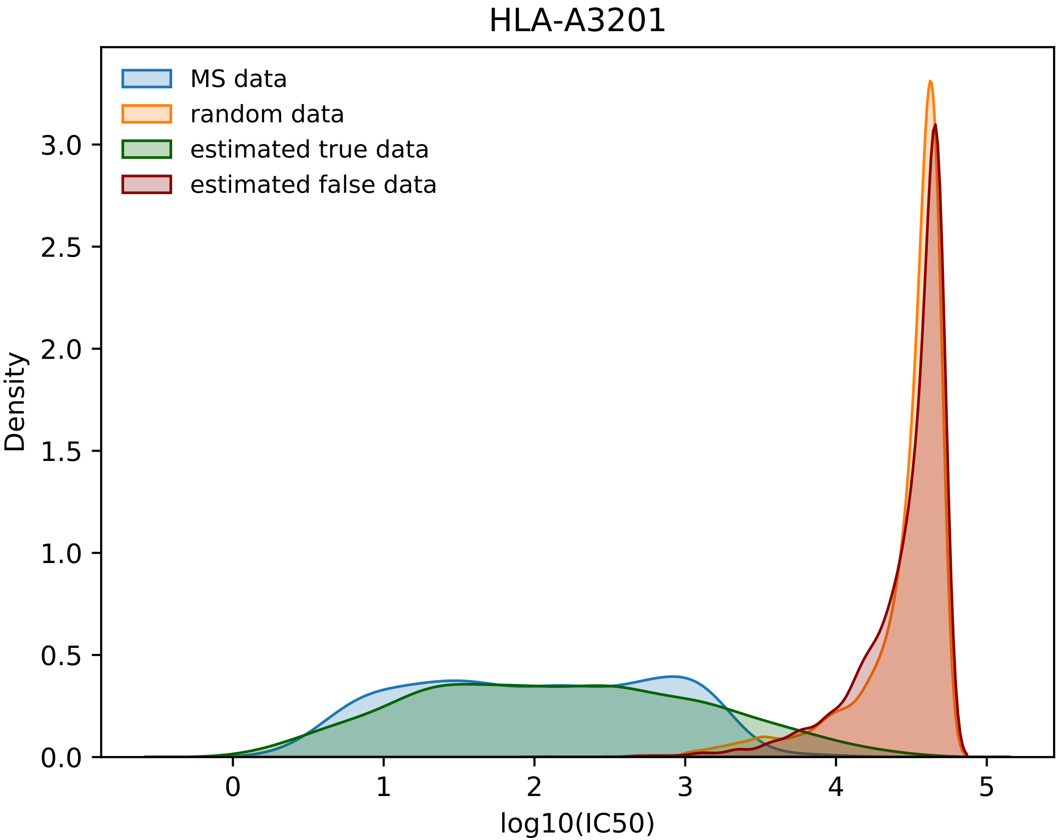

HLA-A3201
MS data
random data
estimated true data
estimated false data
Density
log10(IC50)
0.0
0.5
1.0
1.5
2.0
2.5
3.0
0
1
2
3
4
5

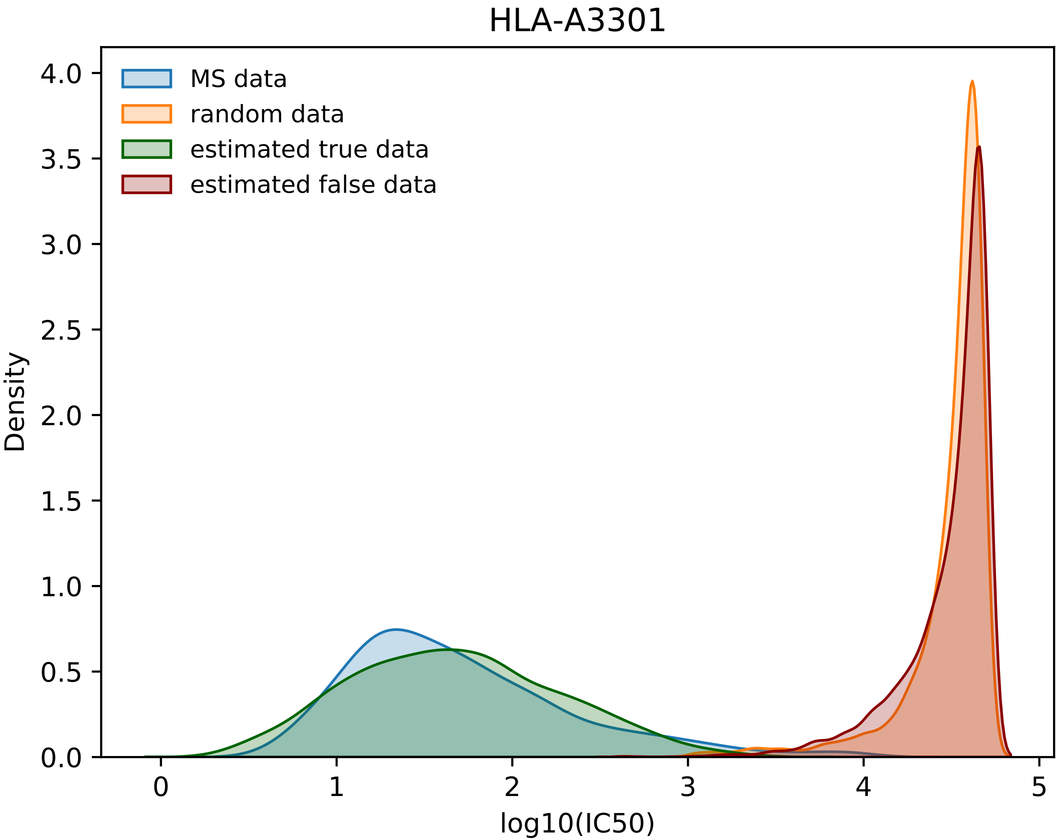
HLA-A3301
MS data
random data
estimated true data
estimated false data
Density
log10(IC50)
0.0
0.5
1.0
1.5
2.0
2.5
3.0
3.5
4.0
0
1
2
3
4
5

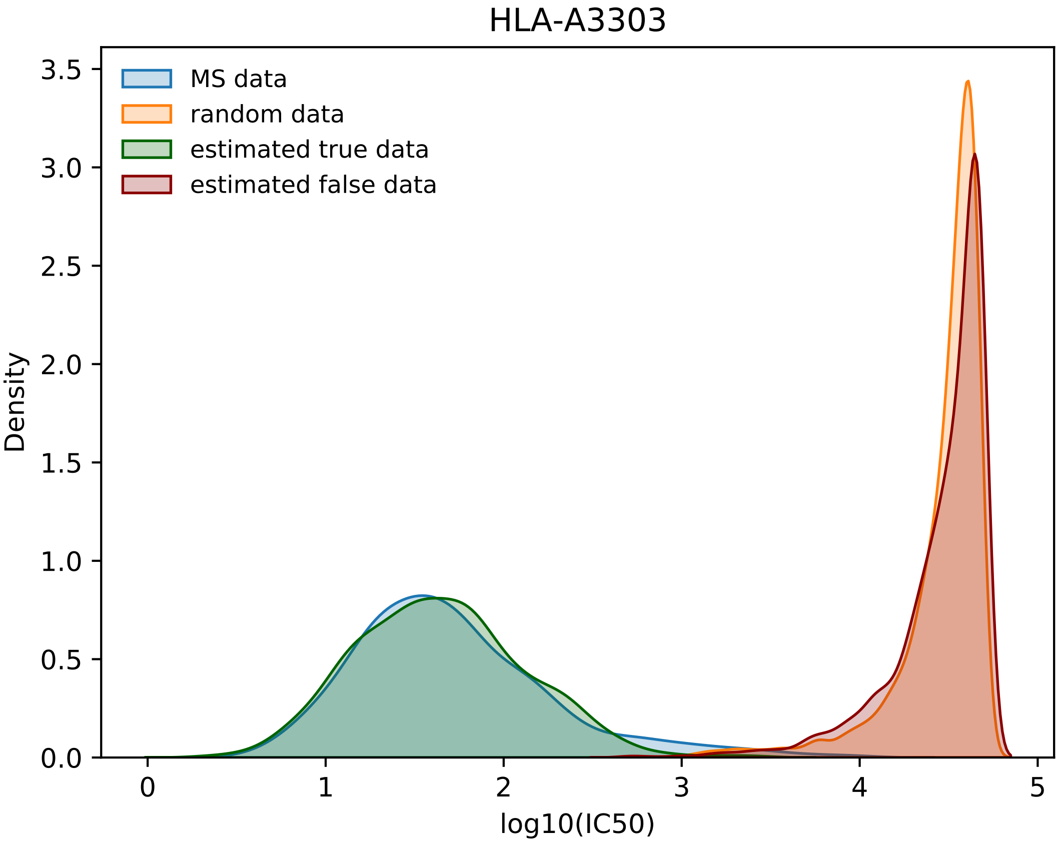
HLA-A3303
MS data
random data
estimated true data
estimated false data
Density
3.5
3.0
2.5
2.0
1.5
1.0
0.5
0.0
0
1
2
3
4
5
log10(IC50)

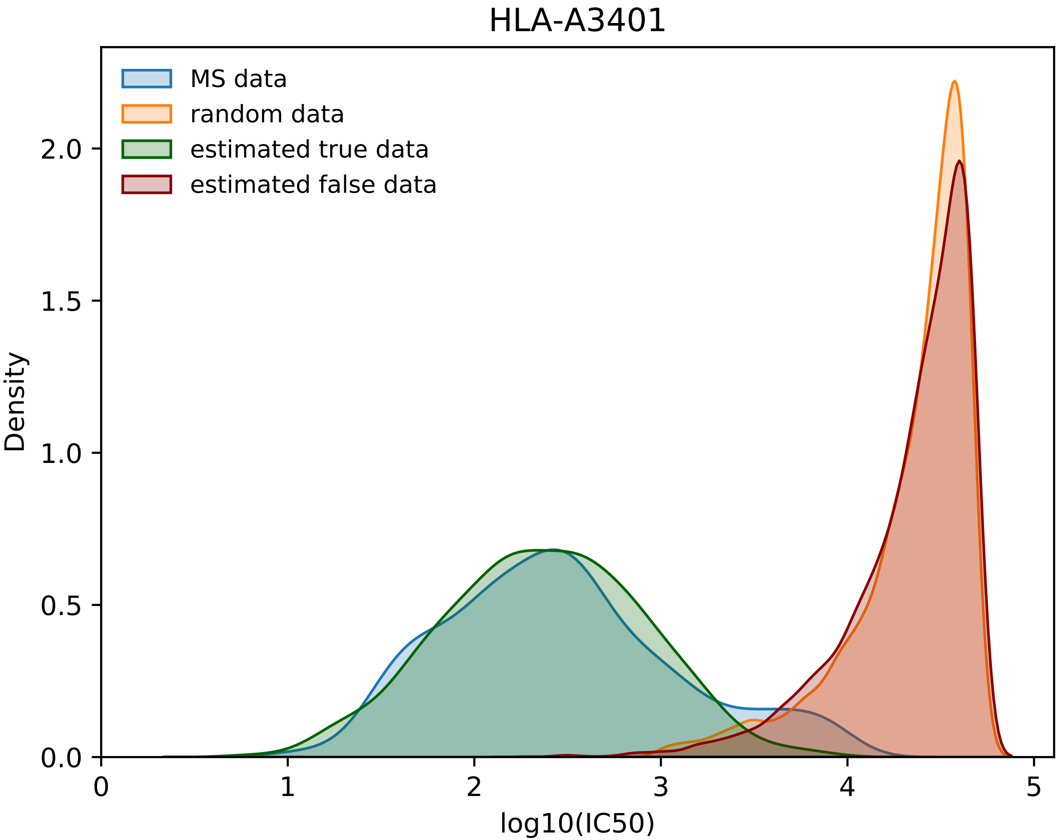
HLA-A3401
MS data
random data
estimated true data
estimated false data
Density
2.0
1.5
1.0
0.5
0.0
0
1
2
3
4
5
log10(IC50)

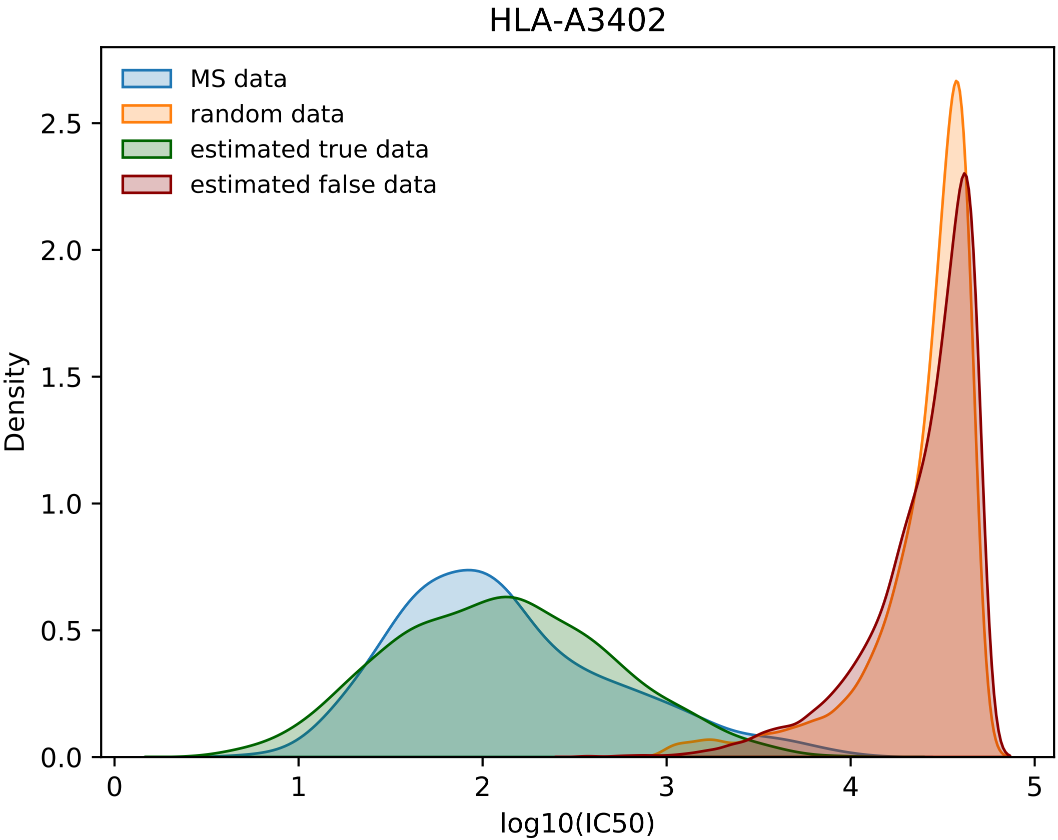
HLA-A3402
MS data
random data
estimated true data
estimated false data
Density
2.5
2.0
1.5
1.0
0.5
0.0
0
1
2
3
4
5
log10(IC50)

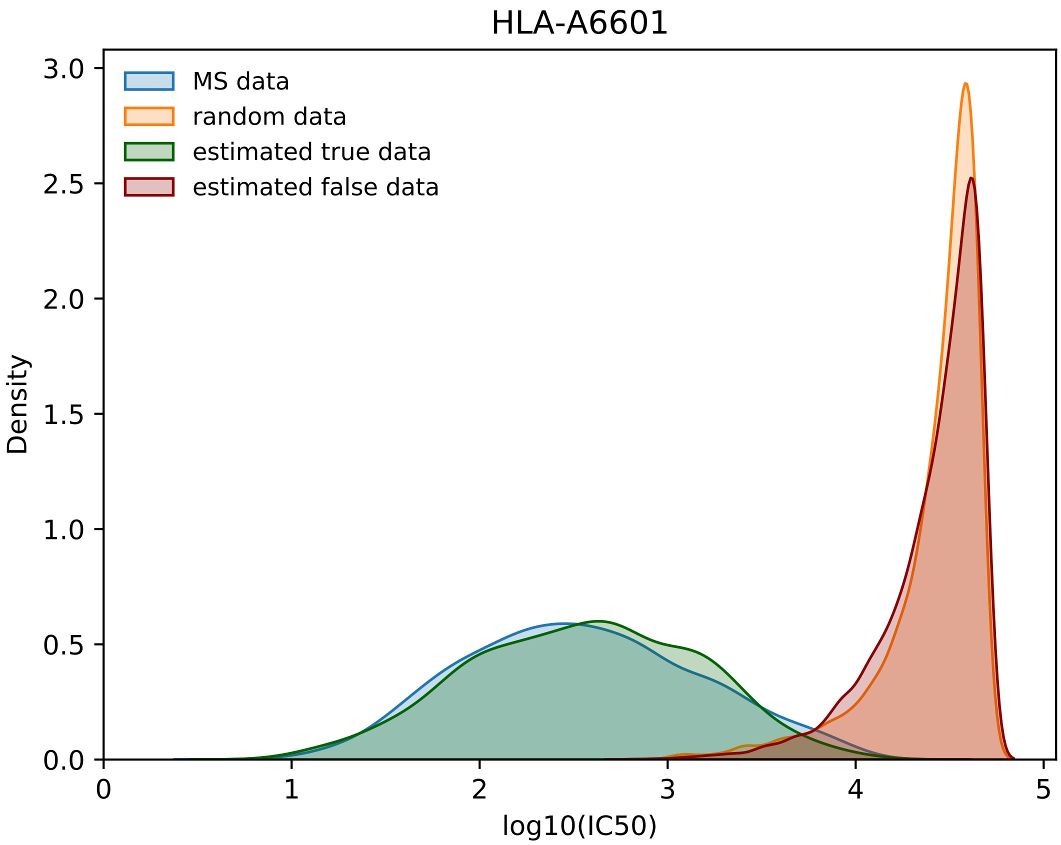

HLA-A6601
MS data
random data
estimated true data
estimated false data
Density
log10(IC50)
0.0
0.5
1.0
1.5
2.0
2.5
3.0
0
1
2
3
4
5

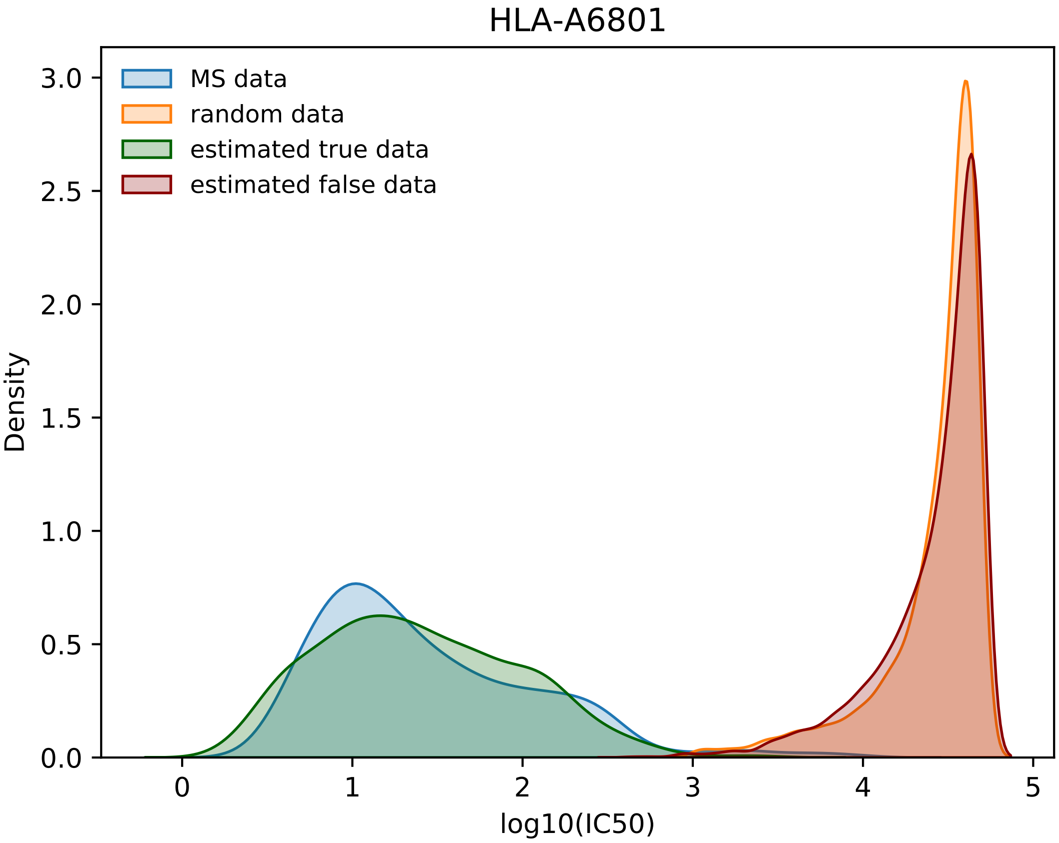
HLA-A6801
MS data
random data
estimated true data
estimated false data
Density
log10(IC50)
0.0
0.5
1.0
1.5
2.0
2.5
3.0
0
1
2
3
4
5

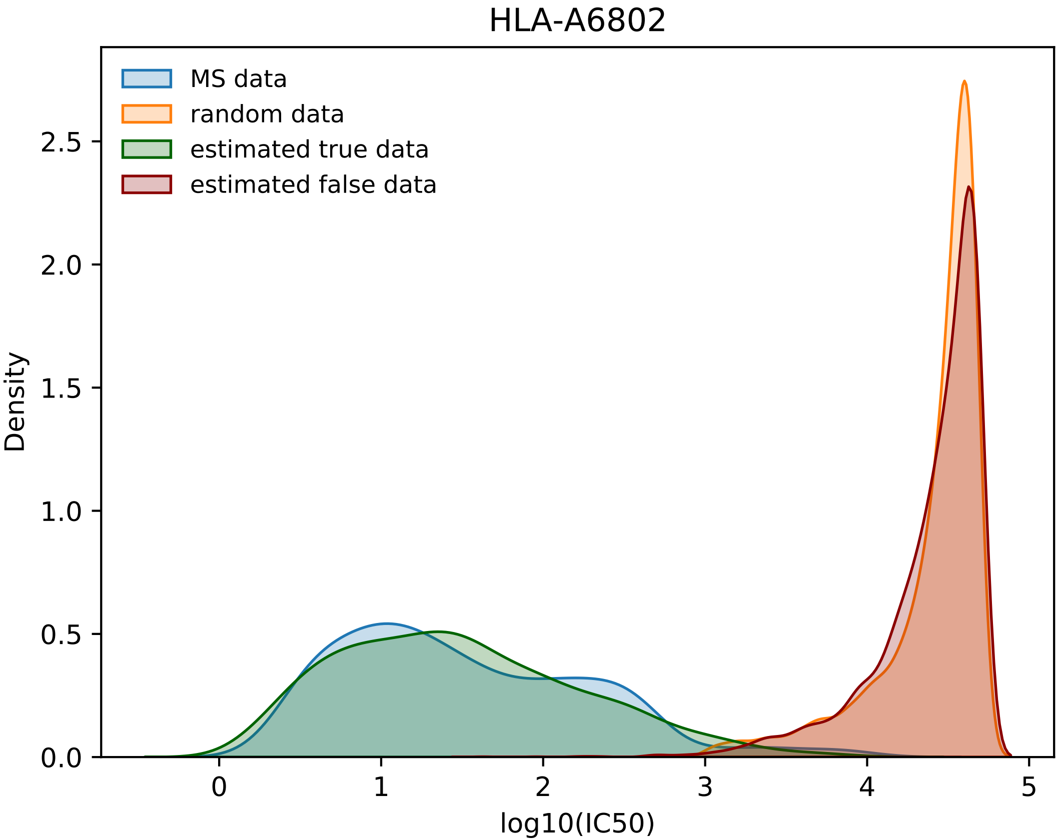
HLA-A6802
MS data
random data
estimated true data
estimated false data
Density
0.0
0.5
1.0
1.5
2.0
2.5
0
1
2
3
4
5
log10(IC50)

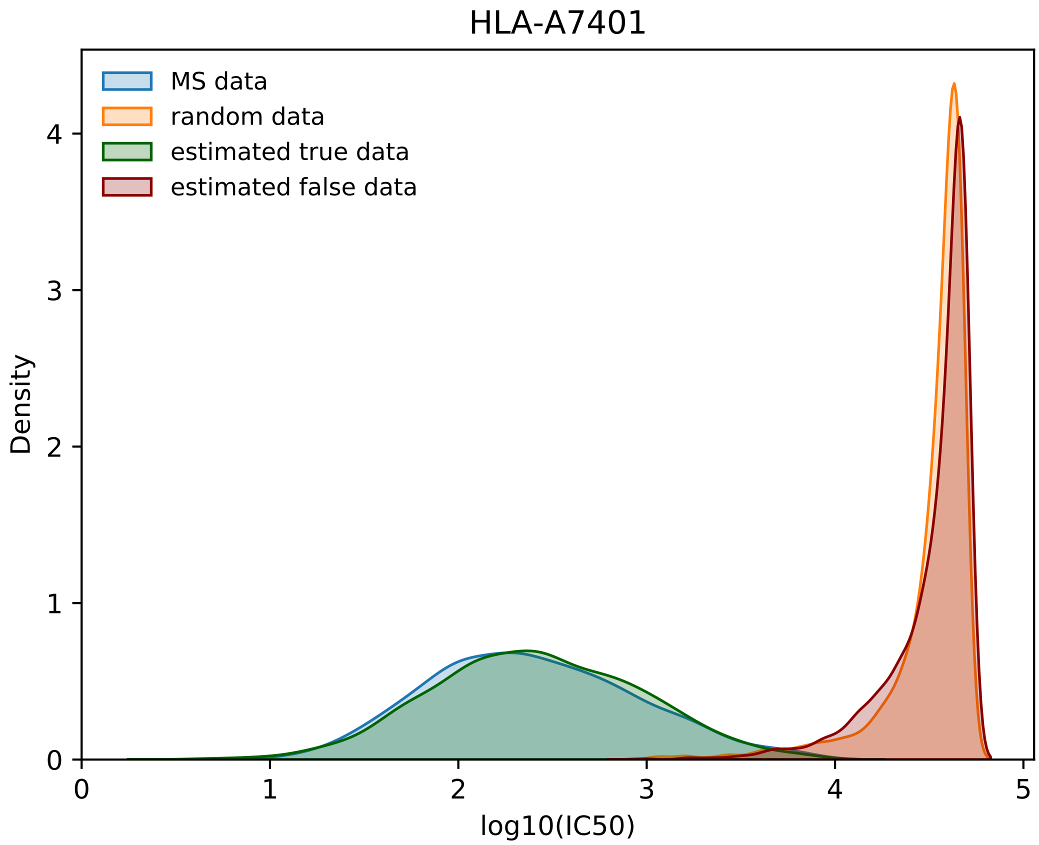

HLA-A7401
MS data
random data
estimated true data
estimated false data
Density
0
1
2
3
4
0
1
2
3
4
5
log10(IC50)

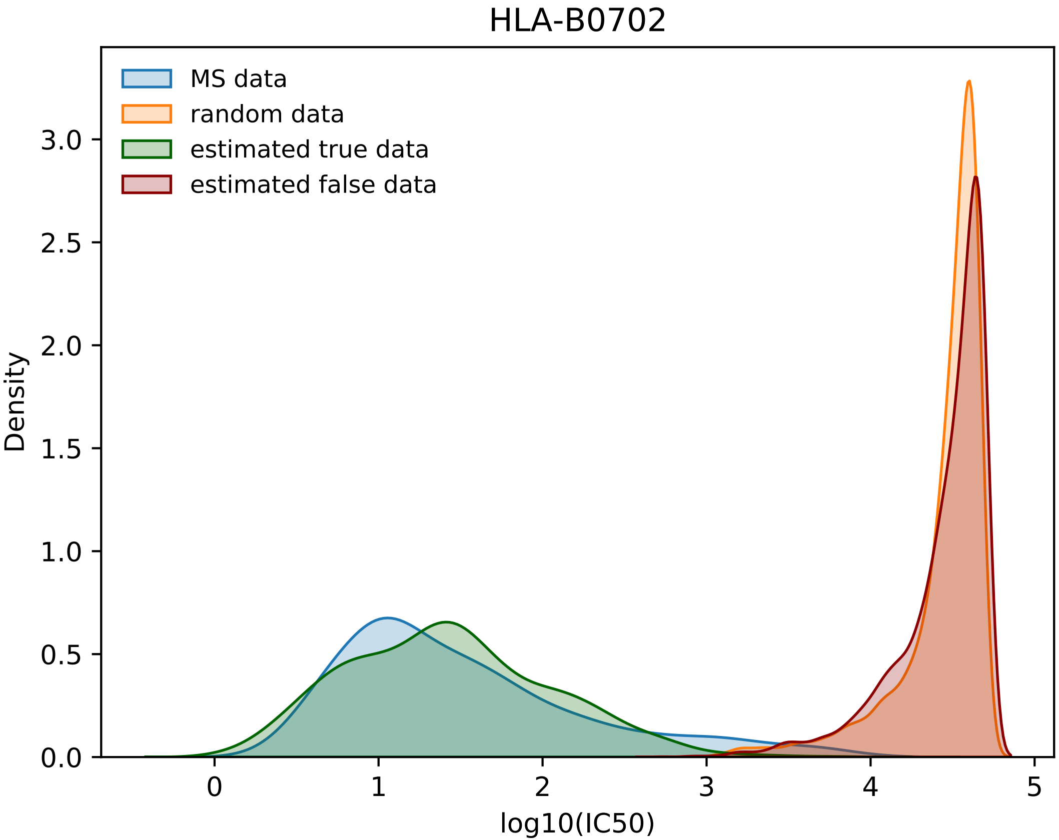
HLA-B0702
MS data
random data
estimated true data
estimated false data
Density
log10(IC50)
0.0
0.5
1.0
1.5
2.0
2.5
3.0
0
1
2
3
4
5

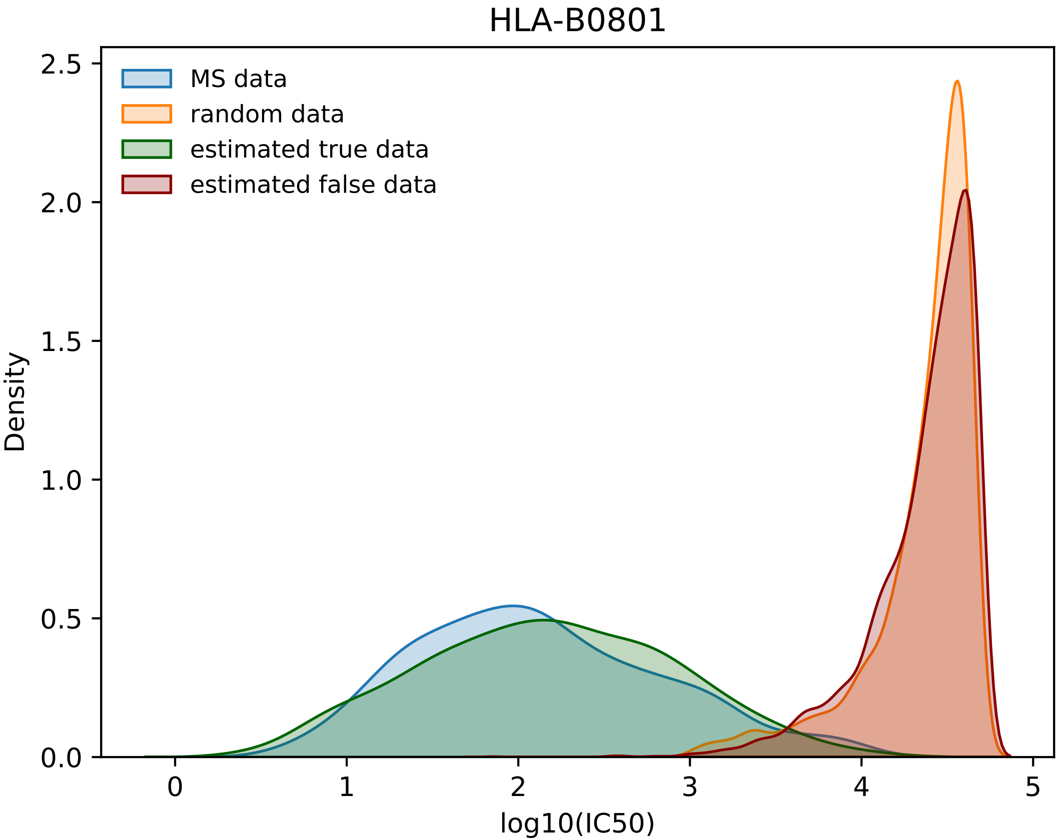
HLA-B0801
MS data
random data
estimated true data
estimated false data
Density
log10(IC50)
0.0
0.5
1.0
1.5
2.0
2.5
0
1
2
3
4
5

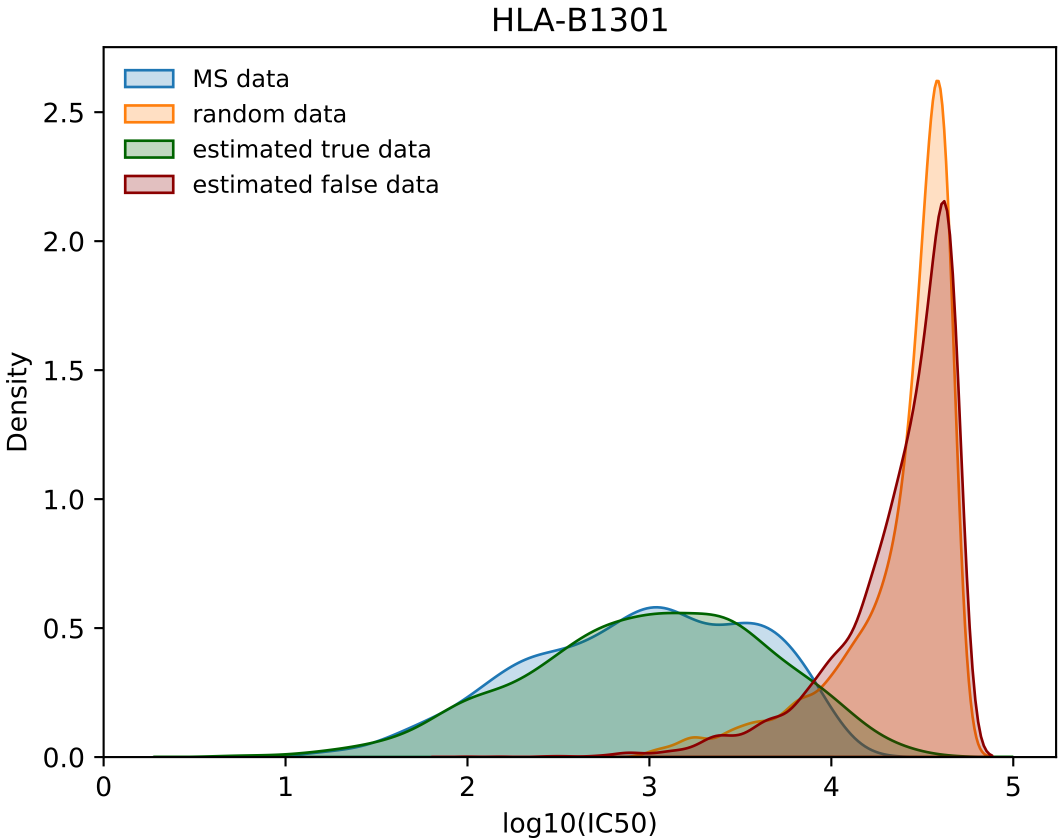
HLA-B1301
MS data
random data
estimated true data
estimated false data
Density
log10(IC50)
0.0
0.5
1.0
1.5
2.0
2.5
0
1
2
3
4
5

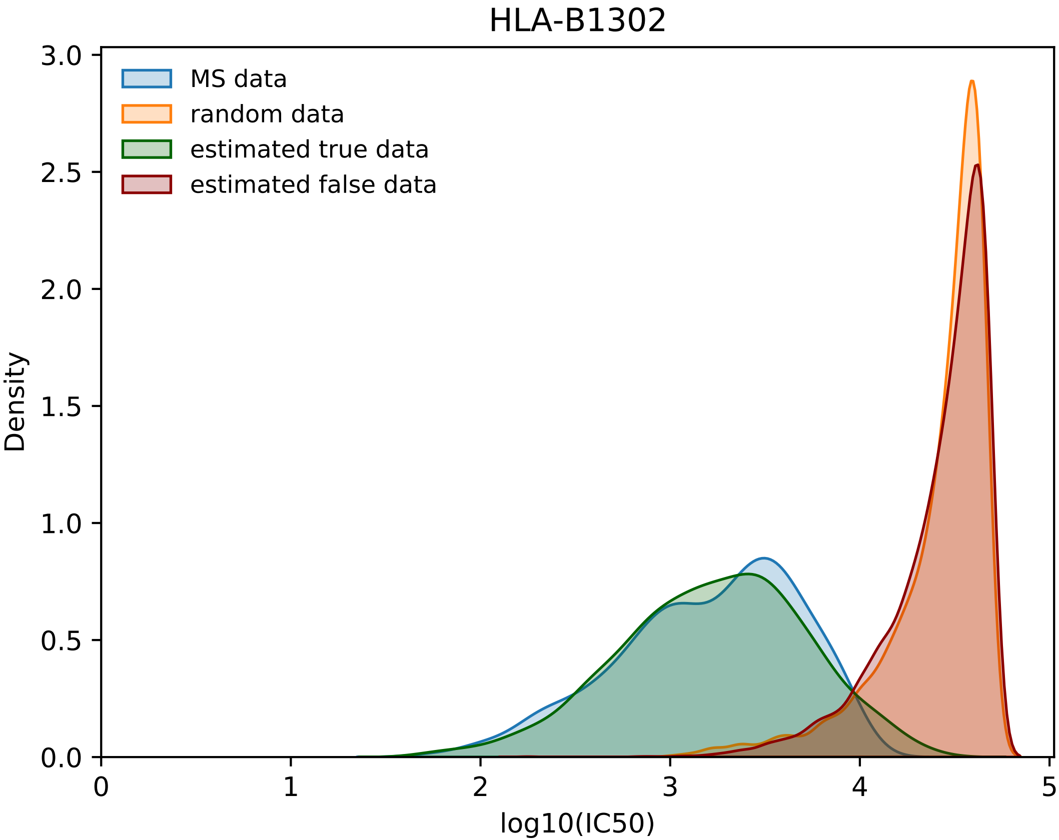
HLA-B1302
MS data
random data
estimated true data
estimated false data
Density
log10(IC50)
0.0
0.5
1.0
1.5
2.0
2.5
3.0
0
1
2
3
4
5

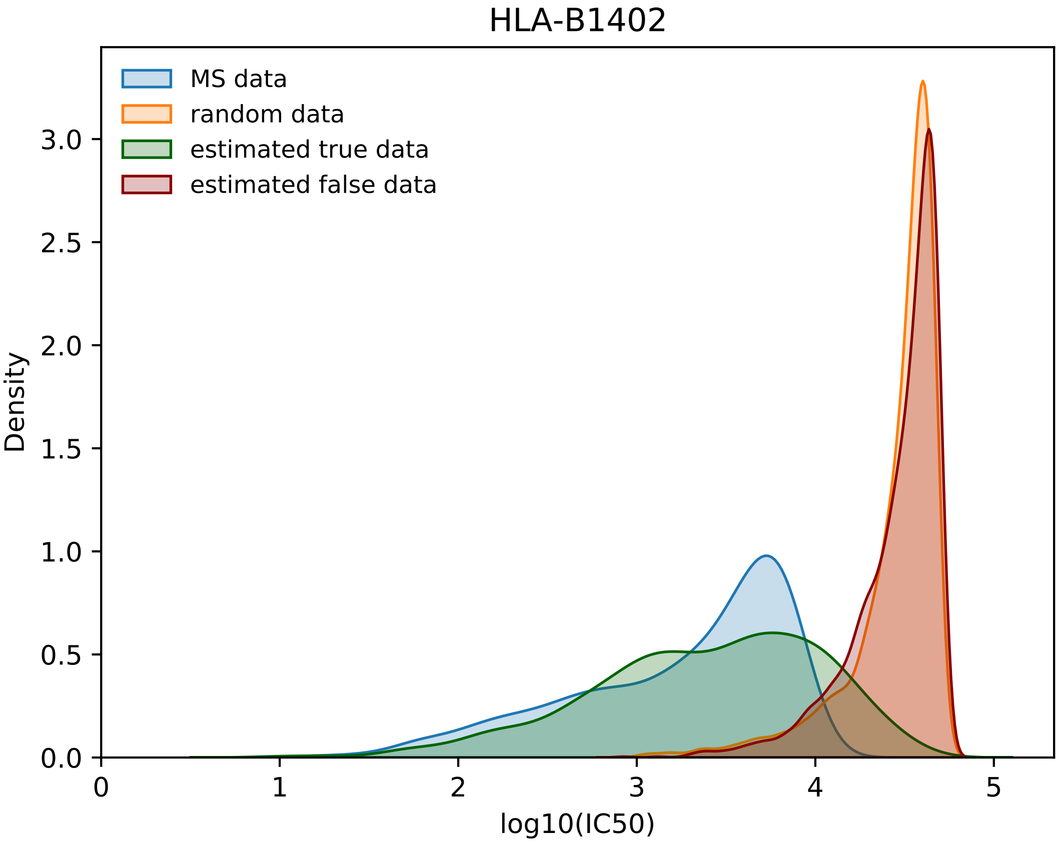
HLA-B1402
MS data
random data
estimated true data
estimated false data
Density
log10(IC50)
0.0
0.5
1.0
1.5
2.0
2.5
3.0
0
1
2
3
4
5

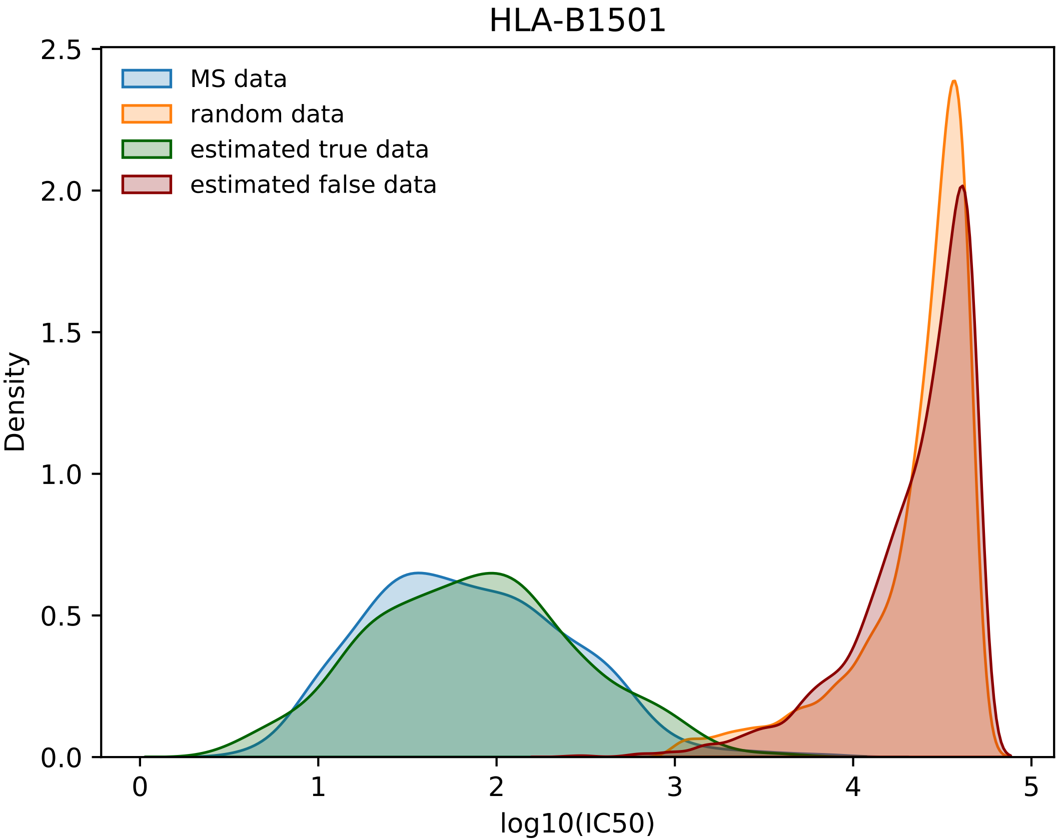
HLA-B1501
MS data
random data
estimated true data
estimated false data
Density
log10(IC50)
0.0
0.5
1.0
1.5
2.0
2.5
0
1
2
3
4
5

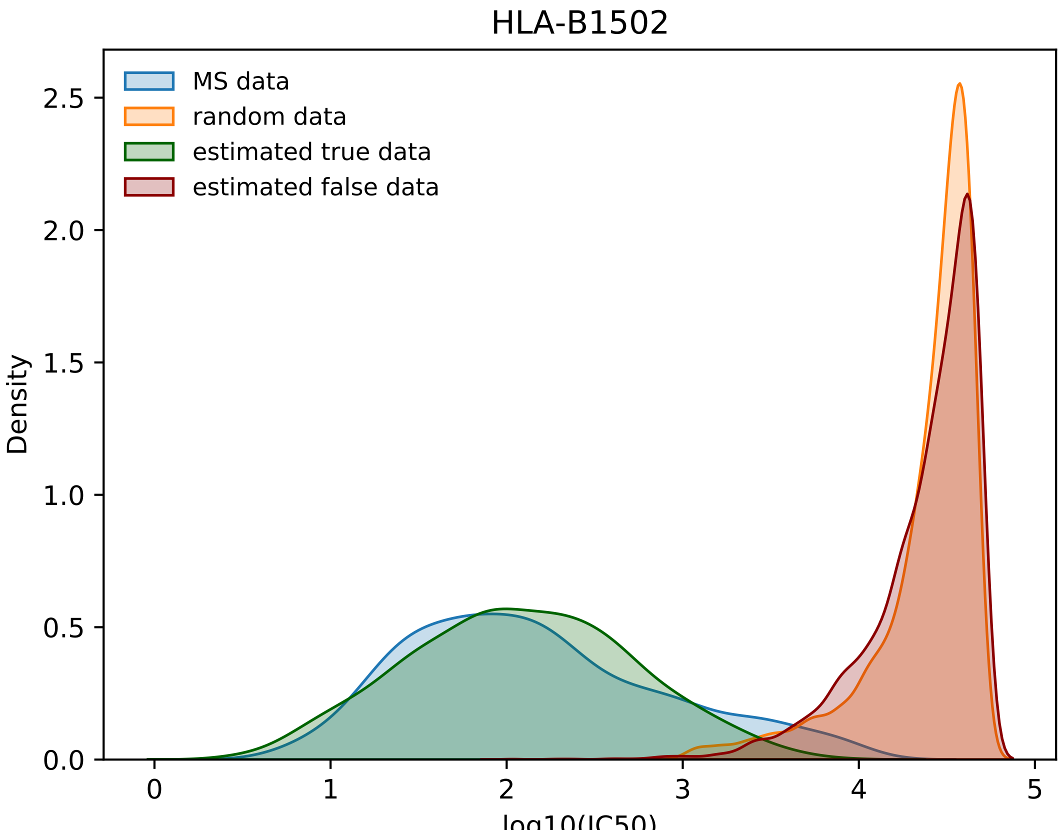
HLA-B1502
MS data
random data
estimated true data
estimated false data
Density
0.0
0.5
1.0
1.5
2.0
2.5
0
1
2
3
4
5
log10(IC50)

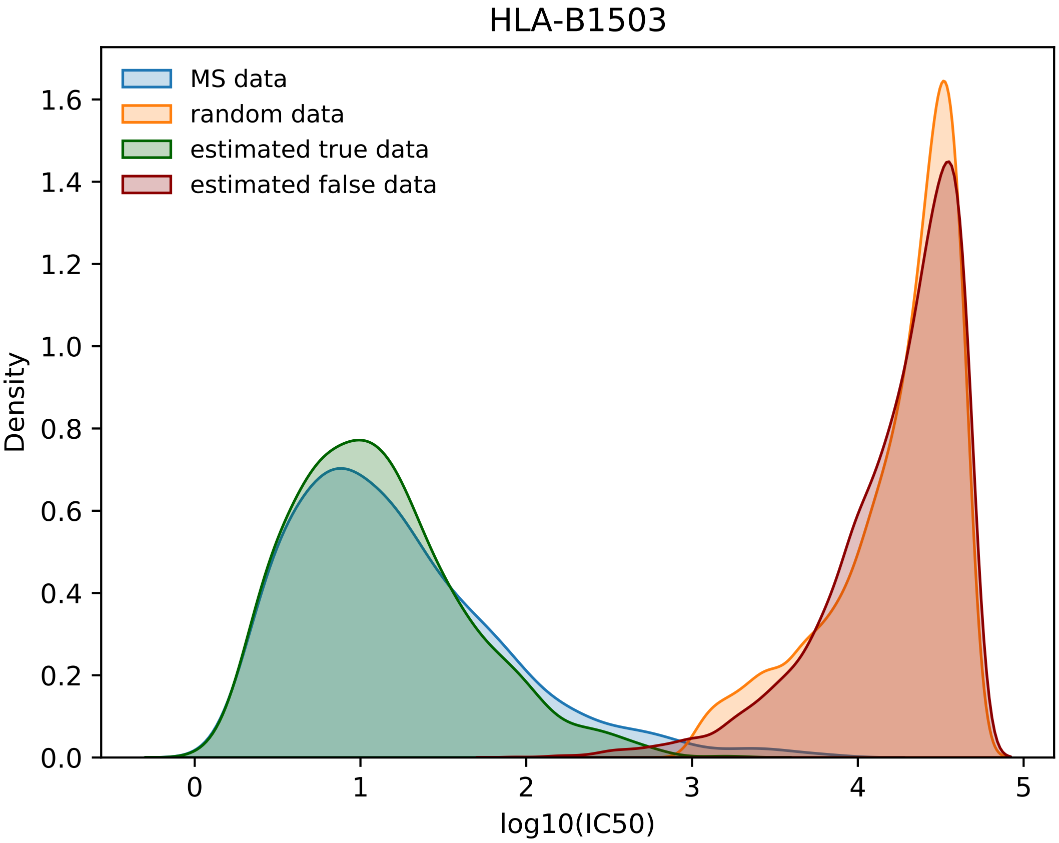

HLA-B1503
MS data
random data
estimated true data
estimated false data
Density
0.0
0.2
0.4
0.6
0.8
1.0
1.2
1.4
1.6
0
1
2
3
4
5
log10(IC50)

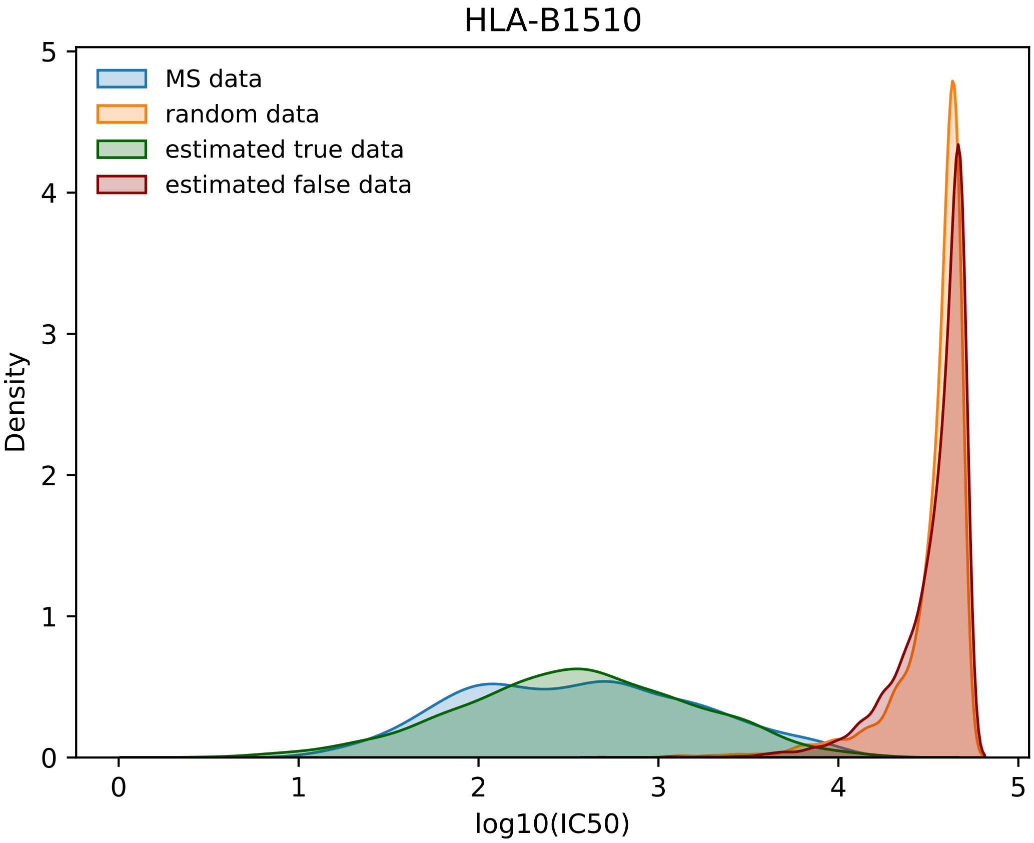

HLA-B1510
MS data
random data
estimated true data
estimated false data
Density
log10(IC50)
0
1
2
3
4
5

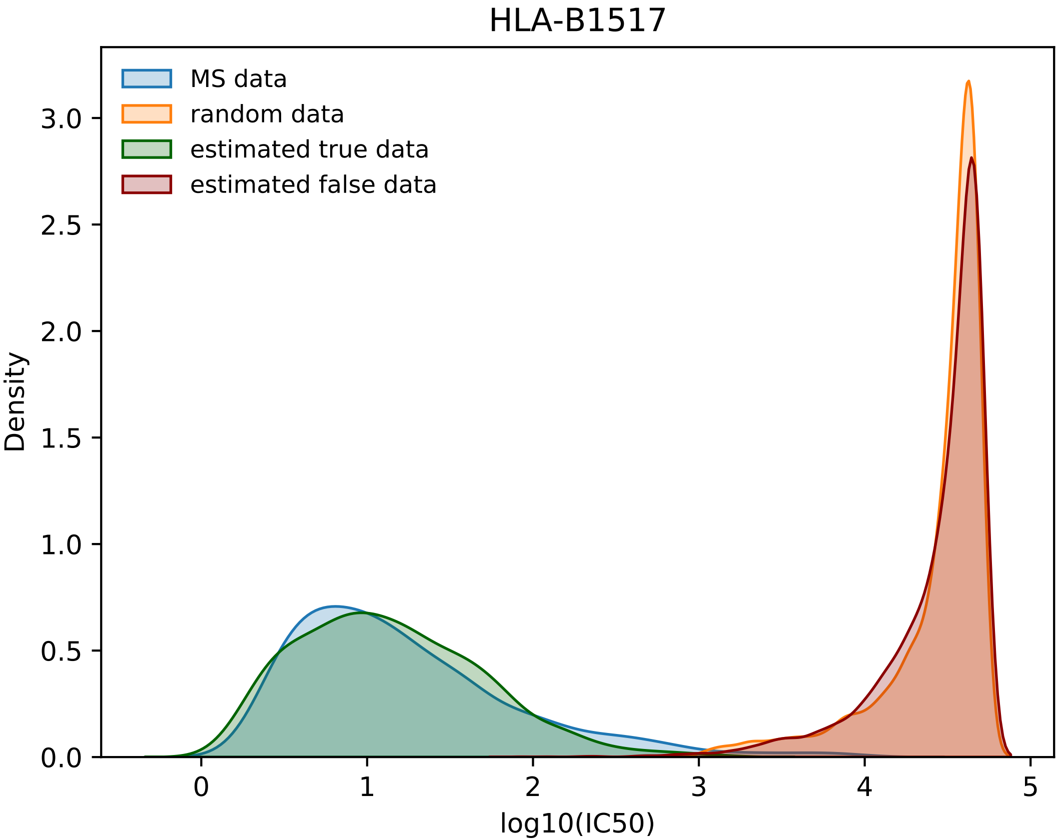
HLA-B1517
MS data
random data
estimated true data
estimated false data
Density
3.0
2.5
2.0
1.5
1.0
0.5
0.0
0
1
2
3
4
5
log10(IC50)

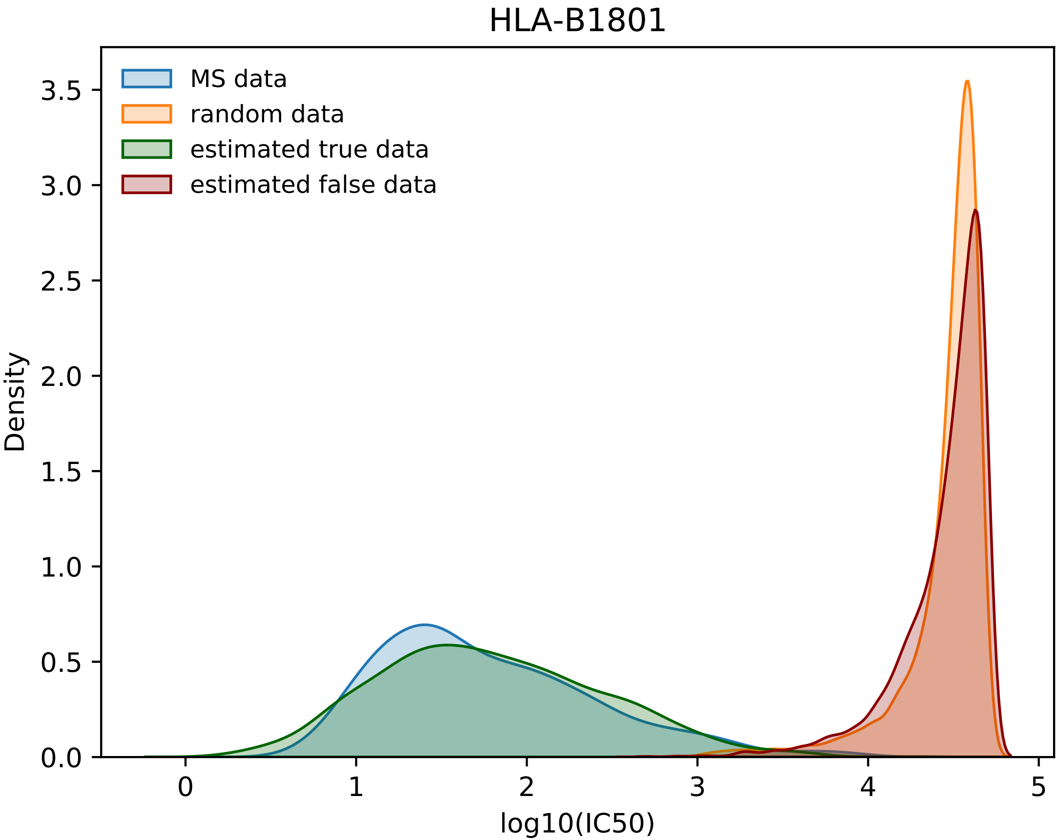
HLA-B1801
MS data
random data
estimated true data
estimated false data
Density
log10(IC50)
0.0
0.5
1.0
1.5
2.0
2.5
3.0
3.5
0
1
2
3
4
5

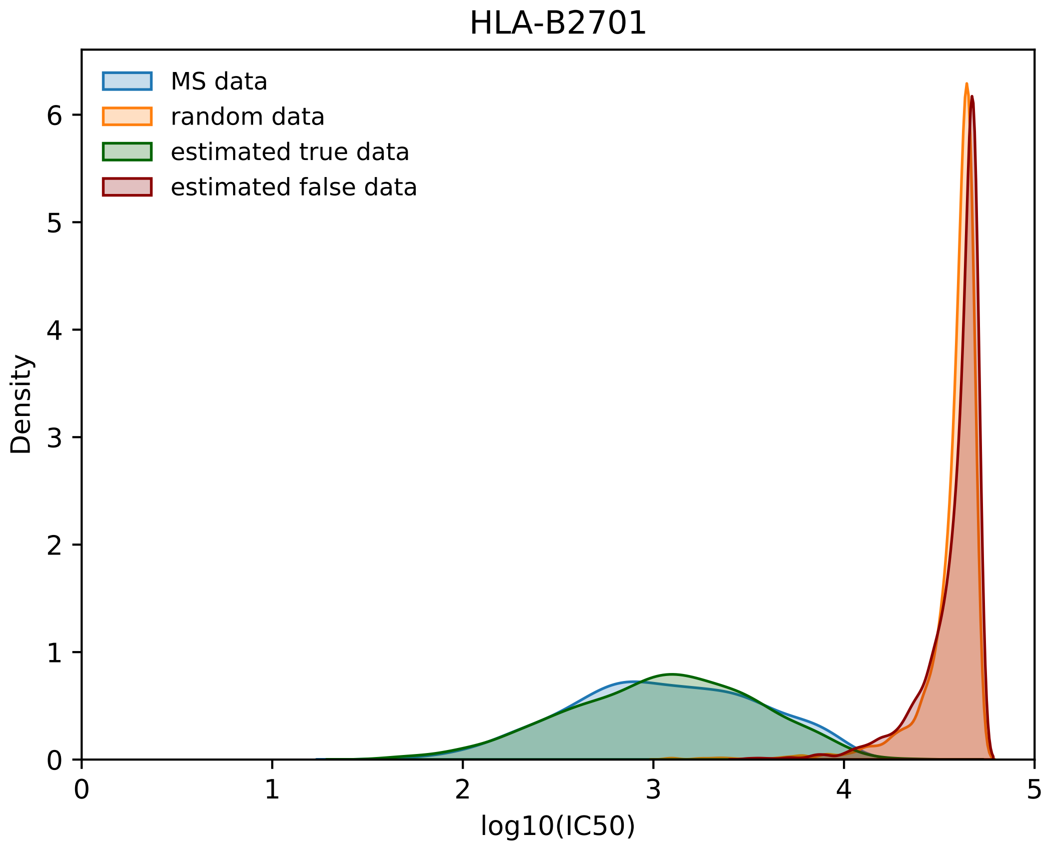
HLA-B2701
MS data
random data
estimated true data
estimated false data
Density
log10(IC50)
0
1
2
3
4
5
6

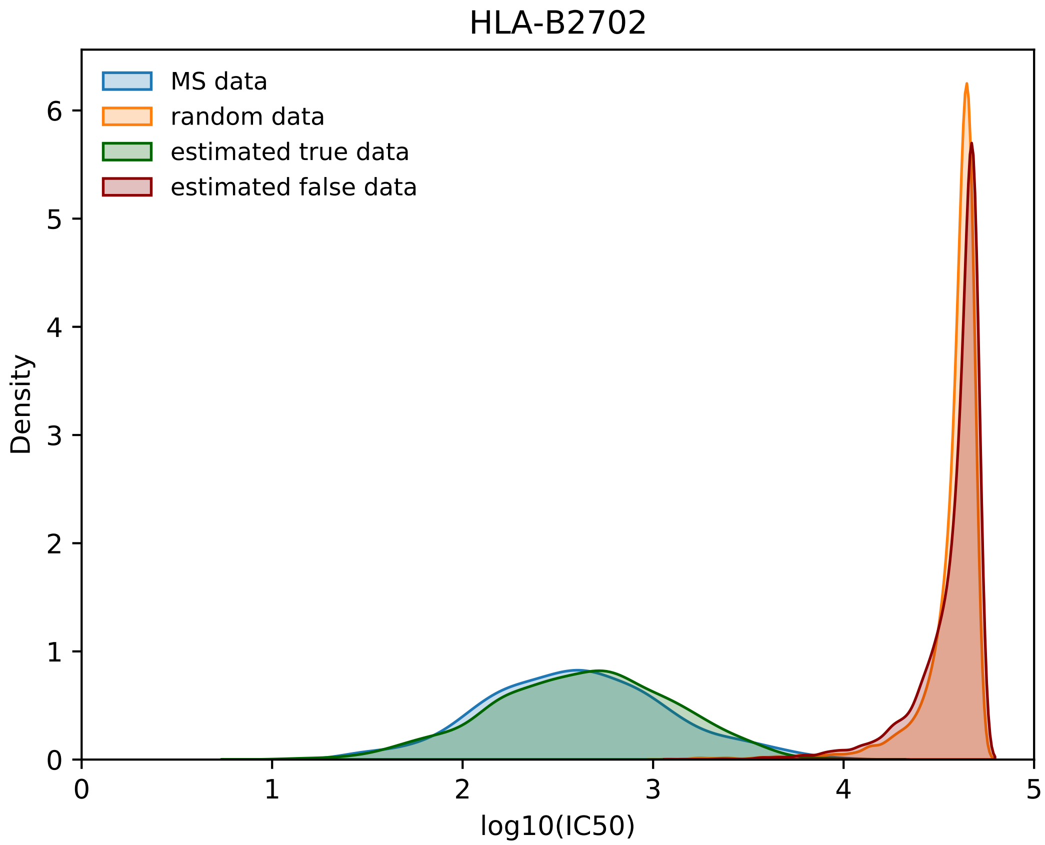
HLA-B2702
MS data
random data
estimated true data
estimated false data
Density
log10(IC50)
0
1
2
3
4
5
6

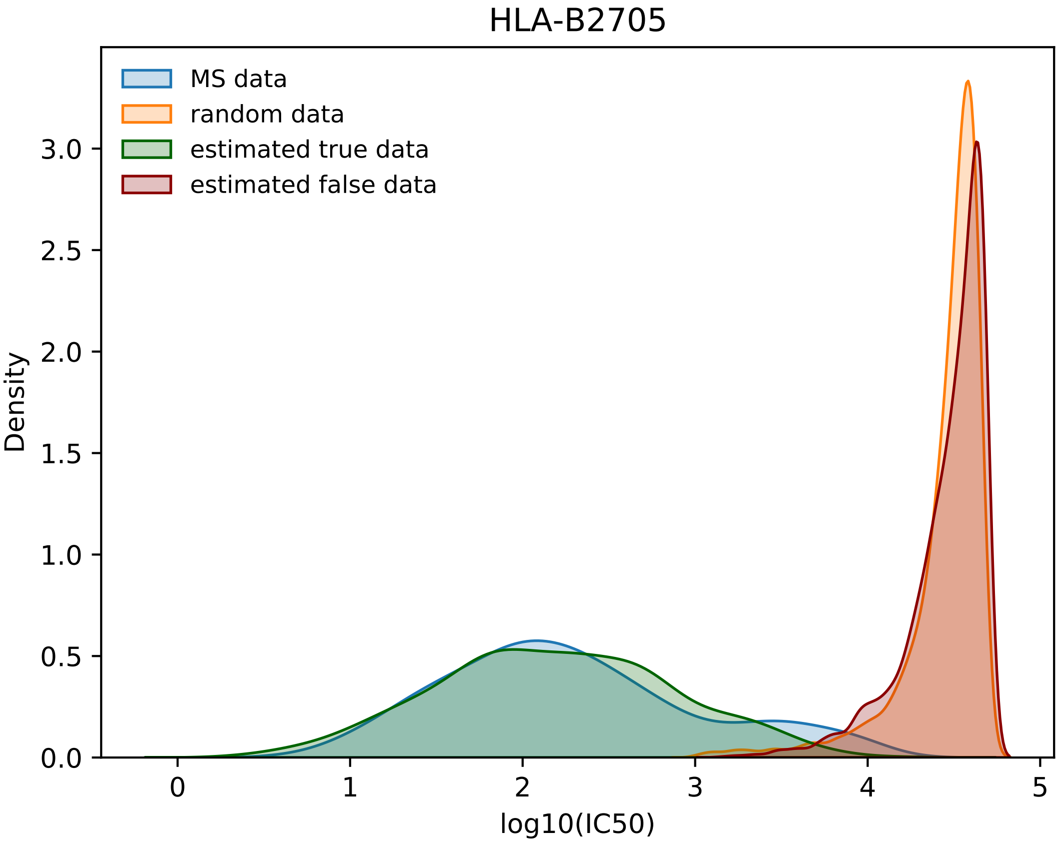
HLA-B2705
MS data
random data
estimated true data
estimated false data
Density
log10(IC50)
0.0
0.5
1.0
1.5
2.0
2.5
3.0
0
1
2
3
4
5

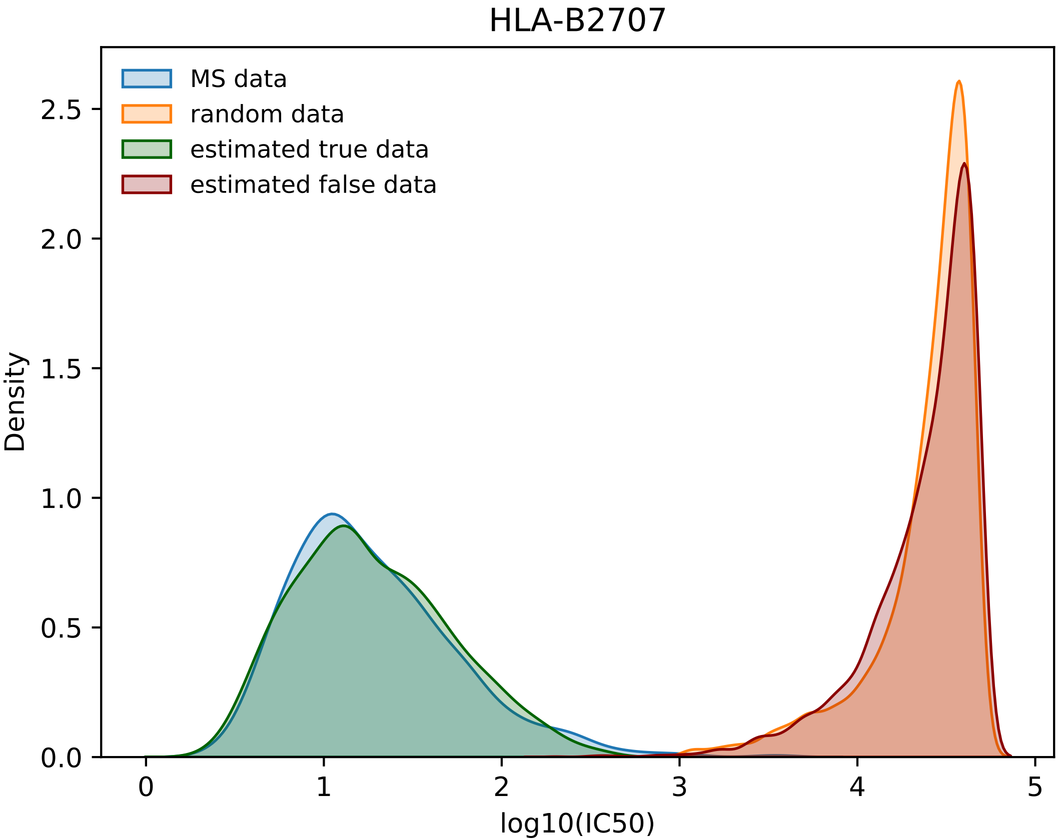

HLA-B2707
MS data
random data
estimated true data
estimated false data
Density
0.0
0.5
1.0
1.5
2.0
2.5
0
1
2
3
4
5
log10(IC50)

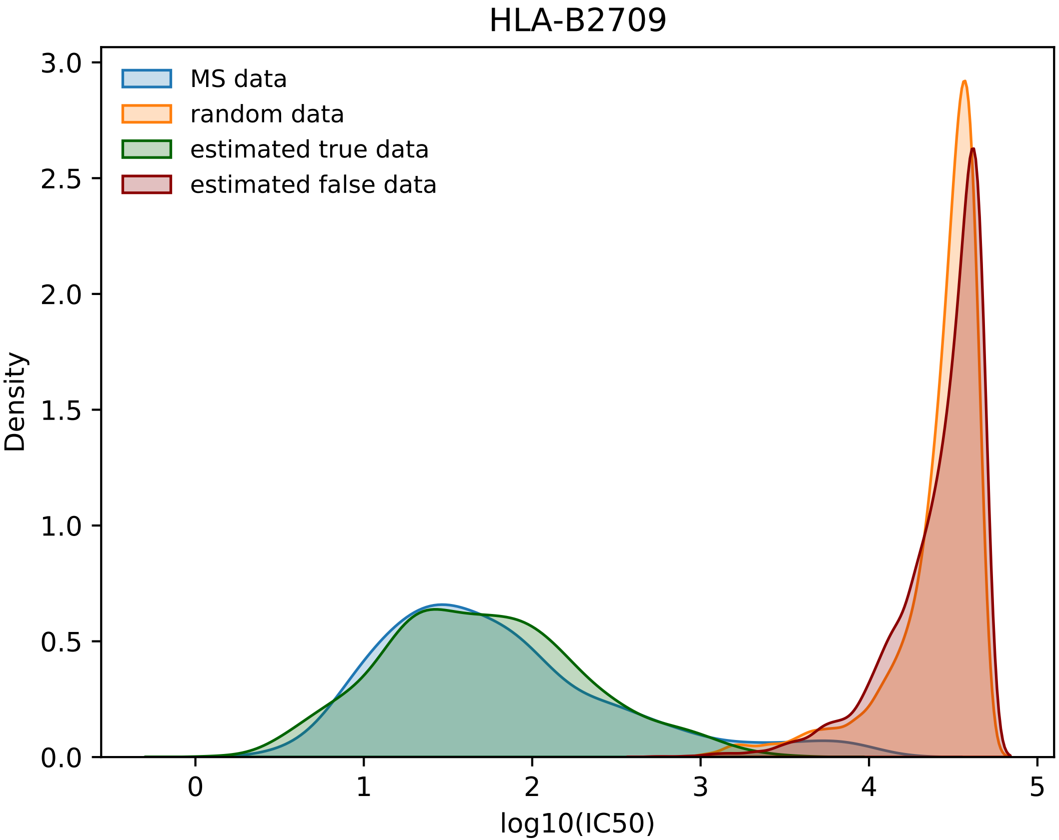
HLA-B2709
MS data
random data
estimated true data
estimated false data
Density
log10(IC50)
0.0
0.5
1.0
1.5
2.0
2.5
3.0
0
1
2
3
4
5

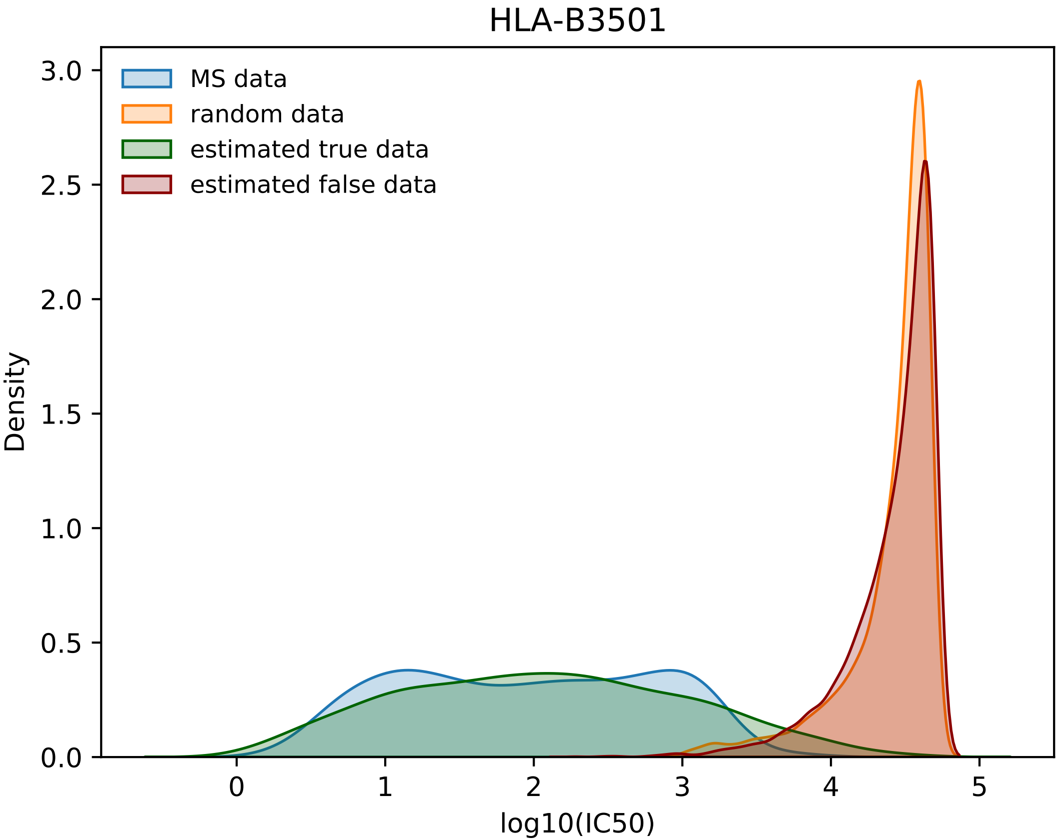

HLA-B3501
MS data
random data
estimated true data
estimated false data
Density
log10(IC50)
3.0
2.5
2.0
1.5
1.0
0.5
0.0
0
1
2
3
4
5

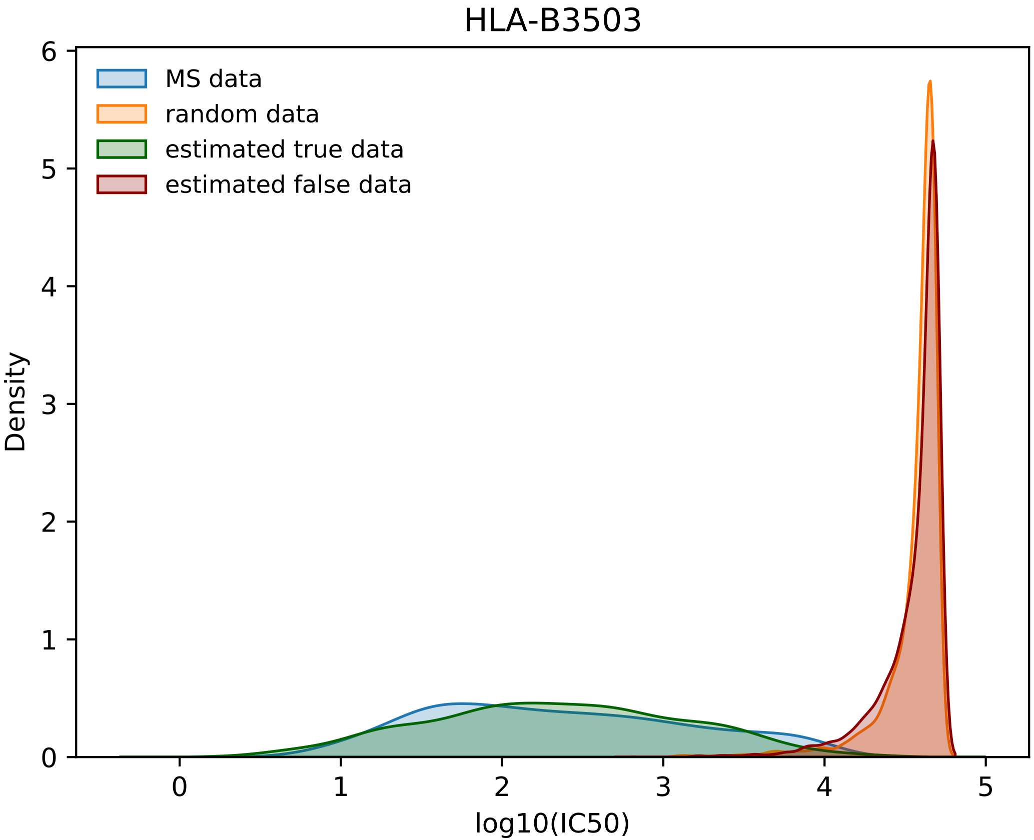
HLA-B3503
MS data
random data
estimated true data
estimated false data
Density
log10(IC50)
0
1
2
3
4
5
6

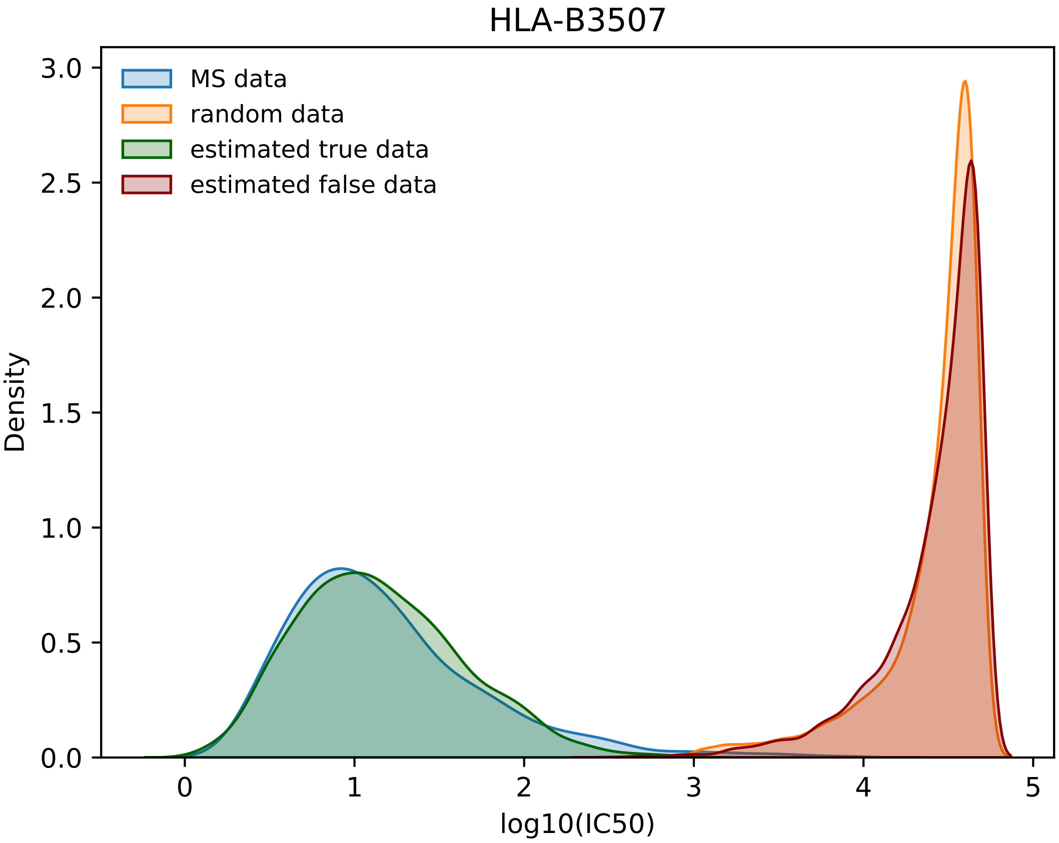
HLA-B3507
MS data
random data
estimated true data
estimated false data
Density
log10(IC50)
0.0
0.5
1.0
1.5
2.0
2.5
3.0
0
1
2
3
4
5

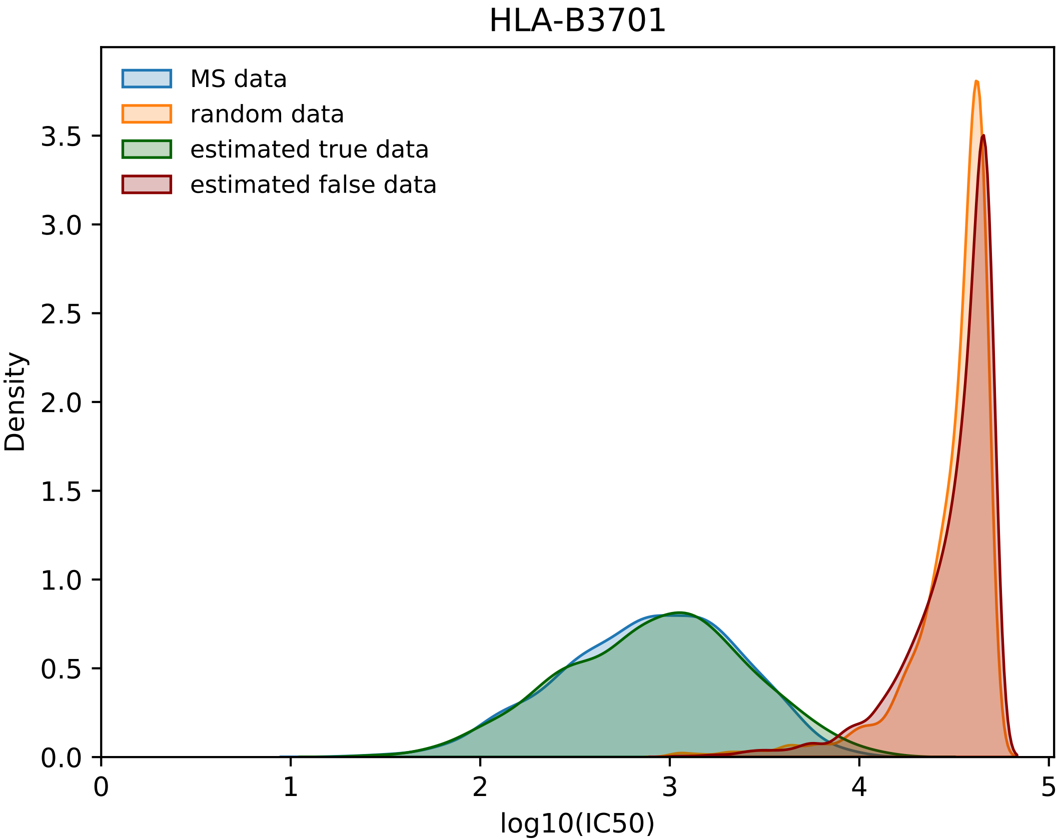
HLA-B3701
MS data
random data
estimated true data
estimated false data
Density
log10(IC50)
0.0
0.5
1.0
1.5
2.0
2.5
3.0
3.5
0
1
2
3
4
5

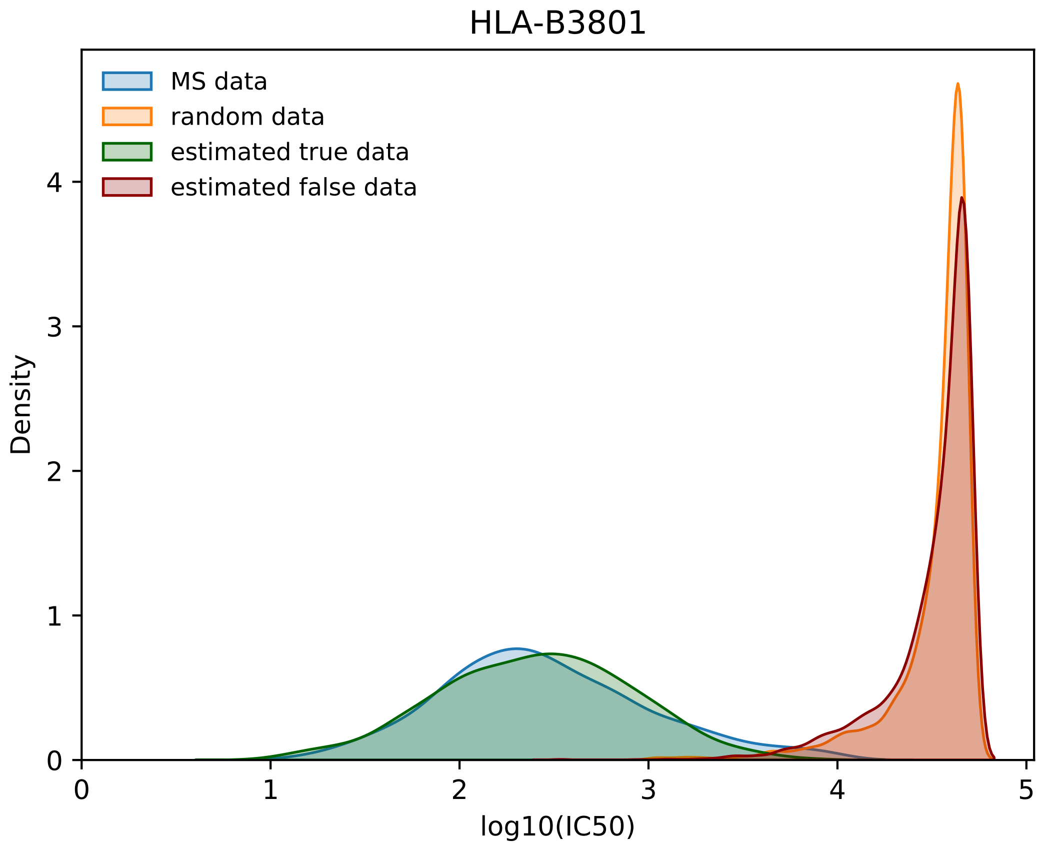
HLA-B3801
MS data
random data
estimated true data
estimated false data
Density
log10(IC50)
0
1
2
3
4
5

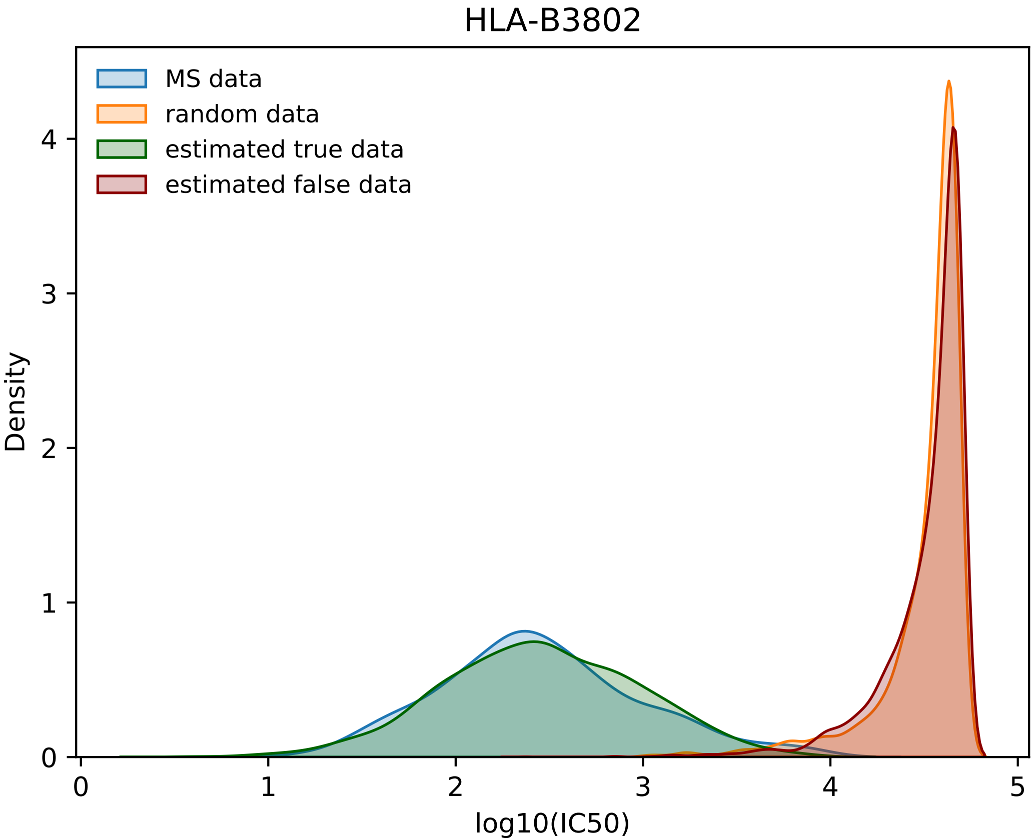
HLA-B3802
MS data
random data
estimated true data
estimated false data
Density
log10(IC50)
0
1
2
3
4
5

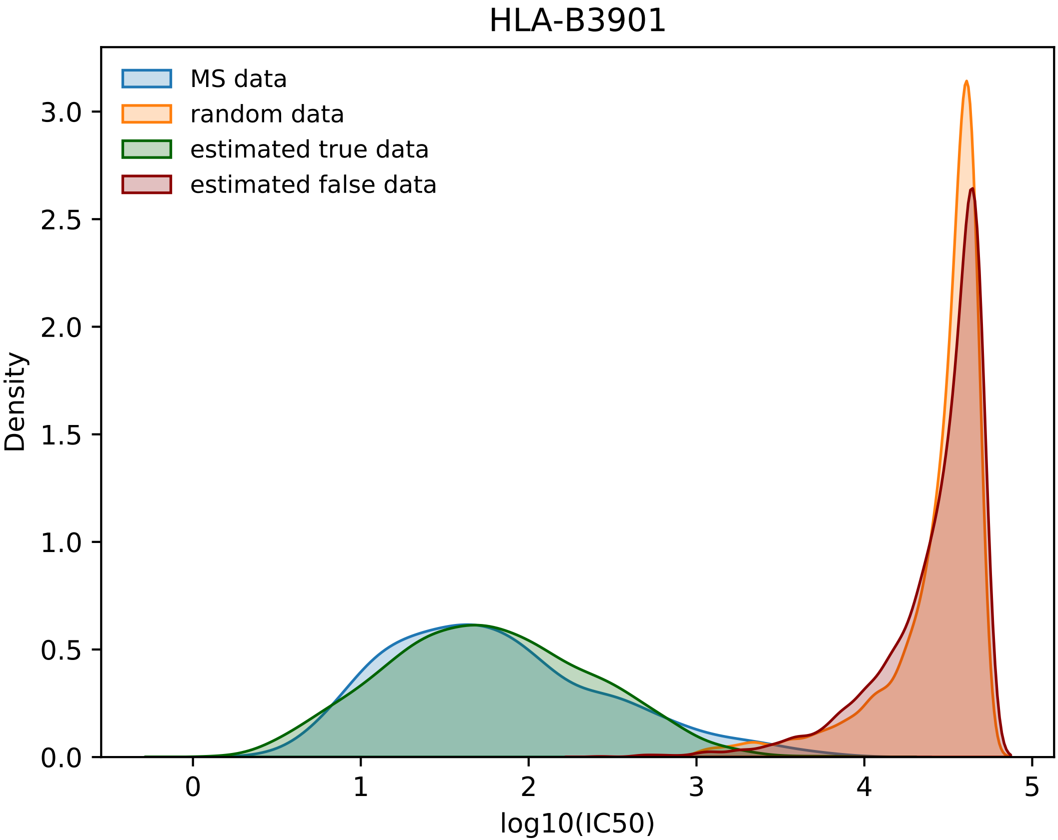

HLA-B3901
MS data
random data
estimated true data
estimated false data
Density
log10(IC50)
0.0
0.5
1.0
1.5
2.0
2.5
3.0
0
1
2
3
4
5

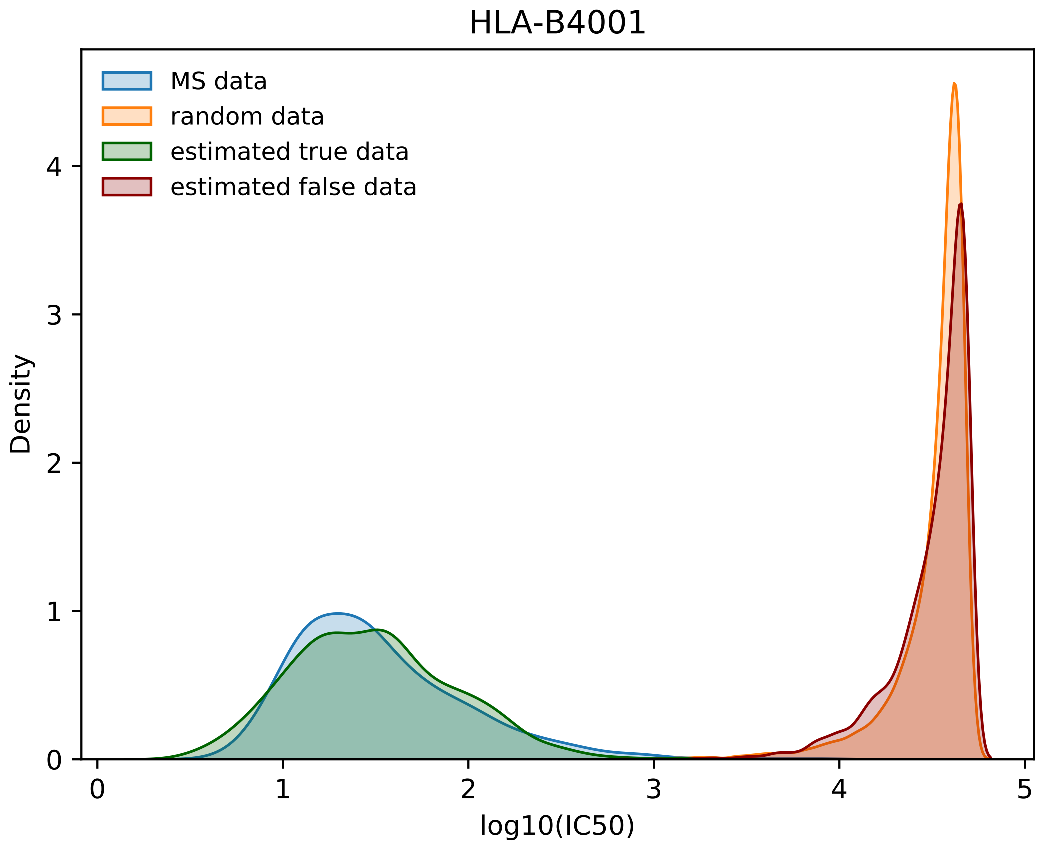
HLA-B4001
MS data
random data
estimated true data
estimated false data
Density
log10(IC50)
0
1
2
3
4
5

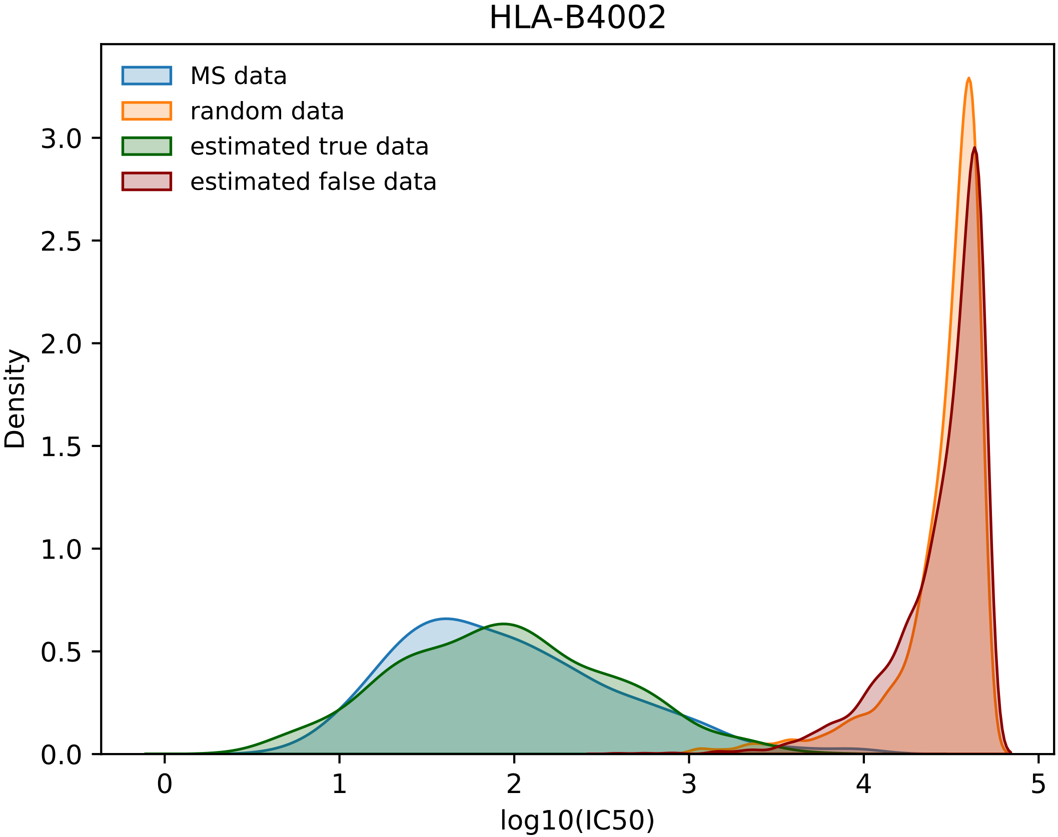
HLA-B4002
MS data
random data
estimated true data
estimated false data
Density
log10(IC50)
0.0
0.5
1.0
1.5
2.0
2.5
3.0
0
1
2
3
4
5

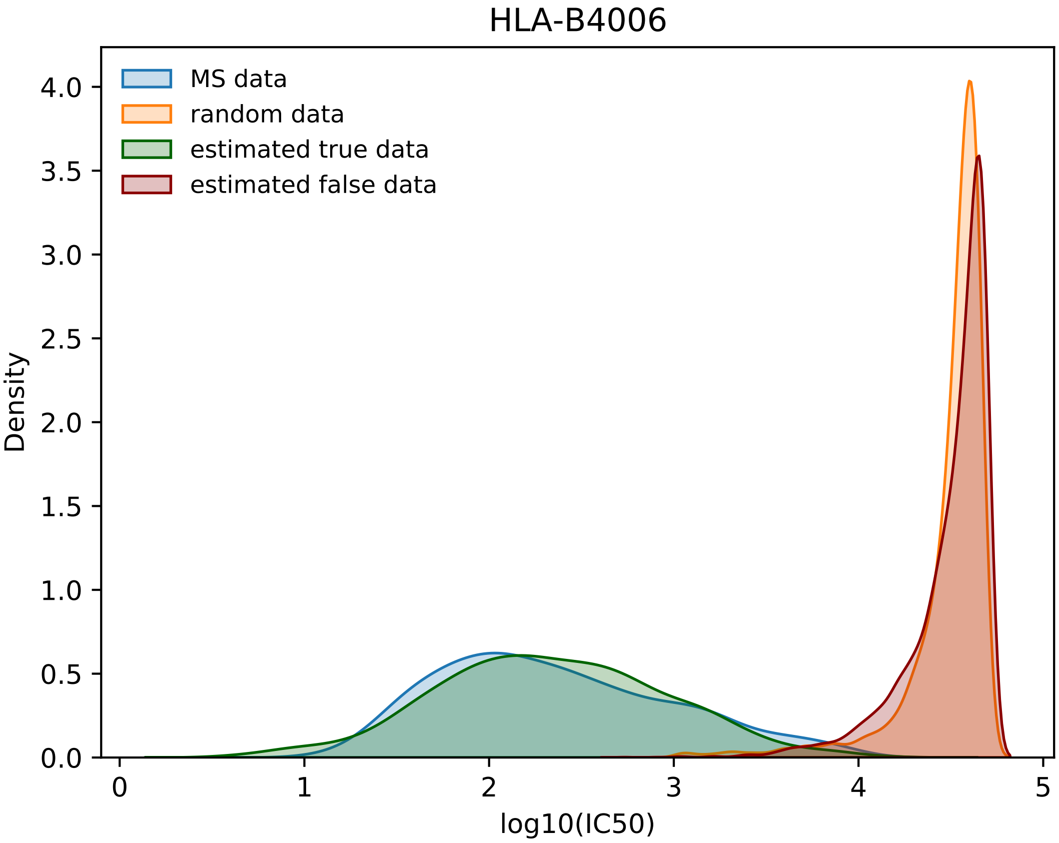
HLA-B4006
MS data
random data
estimated true data
estimated false data
Density
log10(IC50)
0.0
0.5
1.0
1.5
2.0
2.5
3.0
3.5
4.0
0
1
2
3
4
5

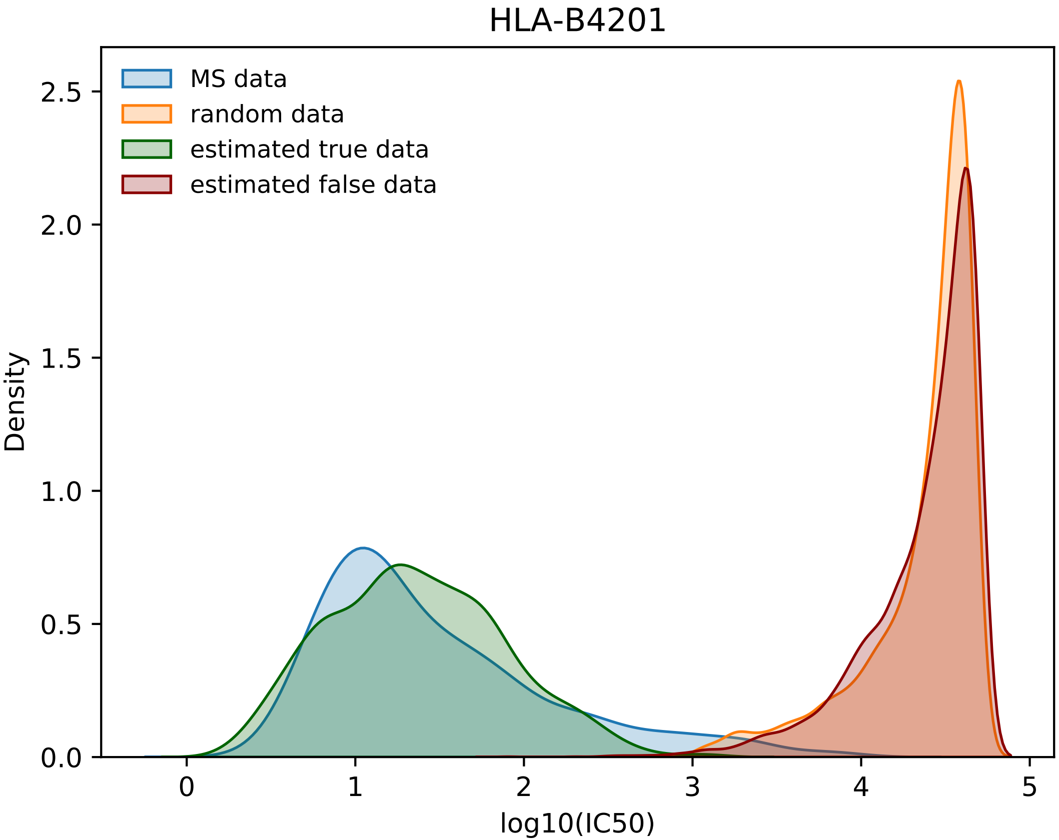
HLA-B4201
MS data
random data
estimated true data
estimated false data
Density
0.0
0.5
1.0
1.5
2.0
2.5
0
1
2
3
4
5
log10(IC50)

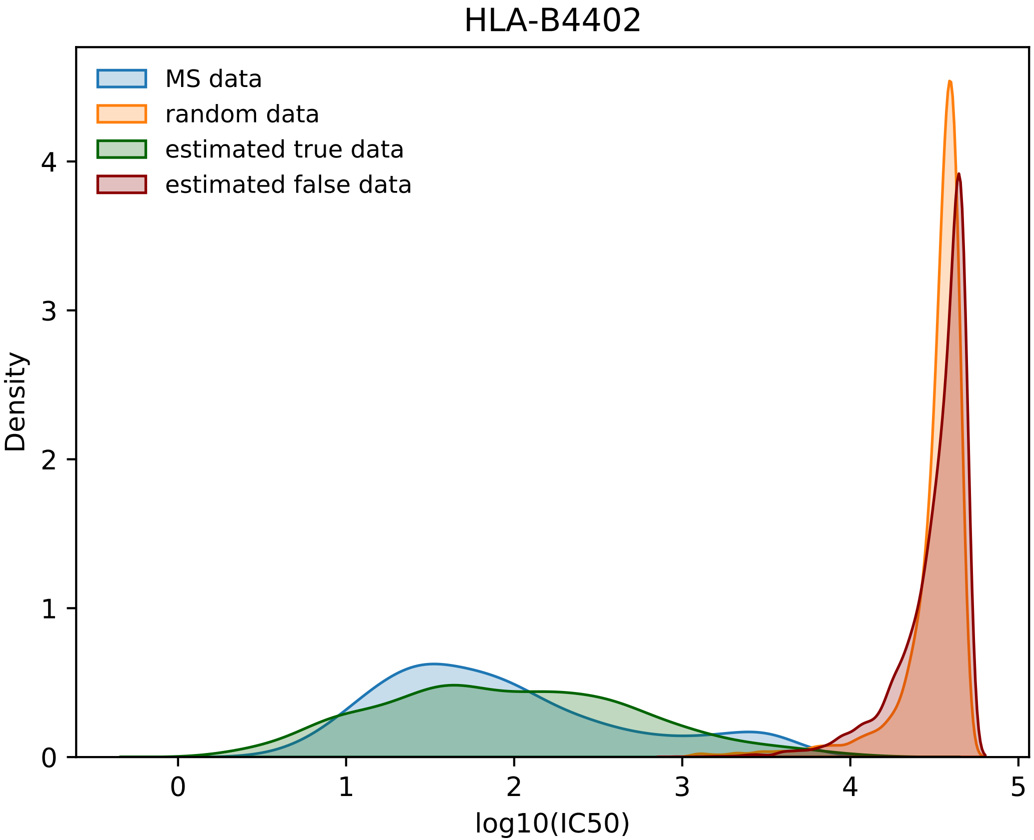
HLA-B4402
MS data
random data
estimated true data
estimated false data
Density
log10(IC50)
0
1
2
3
4
5

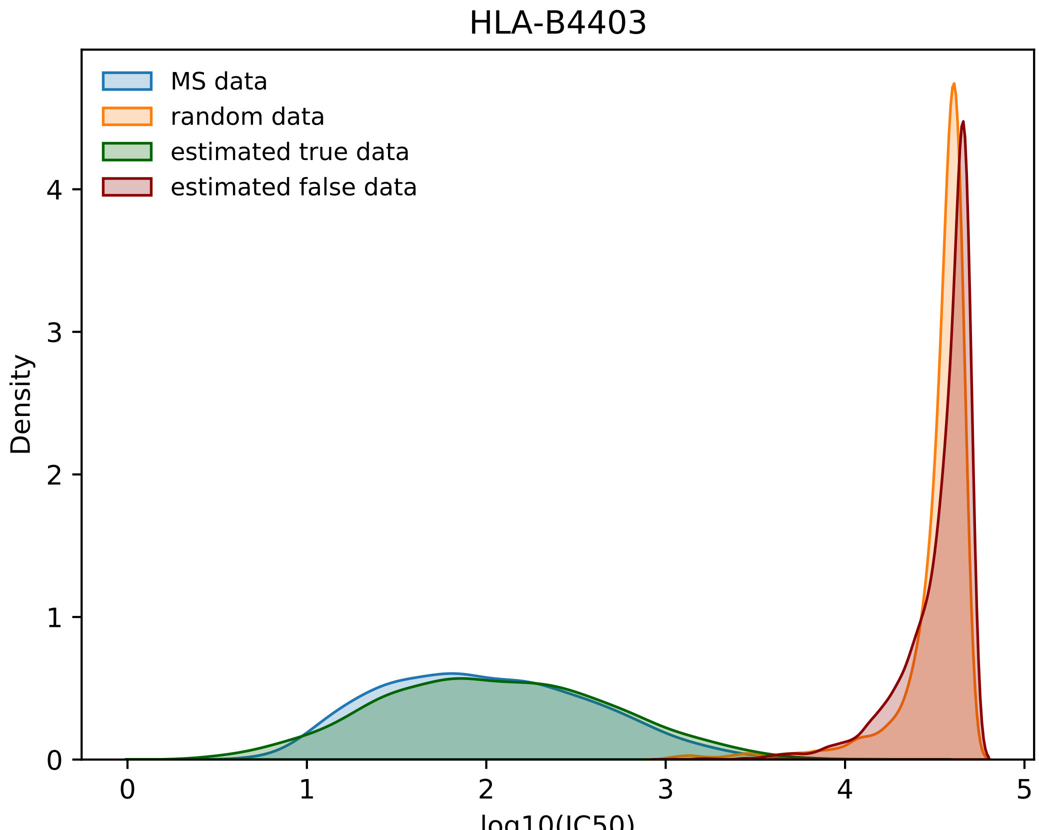
HLA-B4403
MS data
random data
estimated true data
estimated false data
Density
log10(IC50)
0
1
2
3
4
5

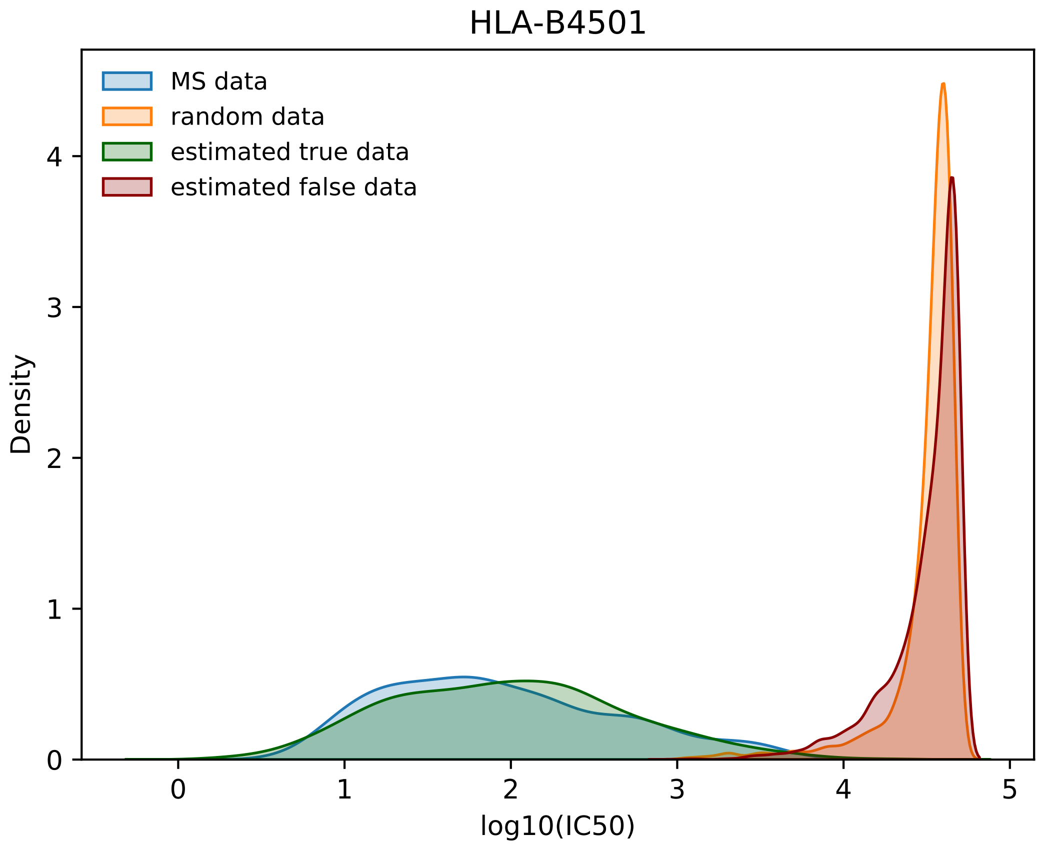
HLA-B4501
MS data
random data
estimated true data
estimated false data
Density
log10(IC50)
0
1
2
3
4
5

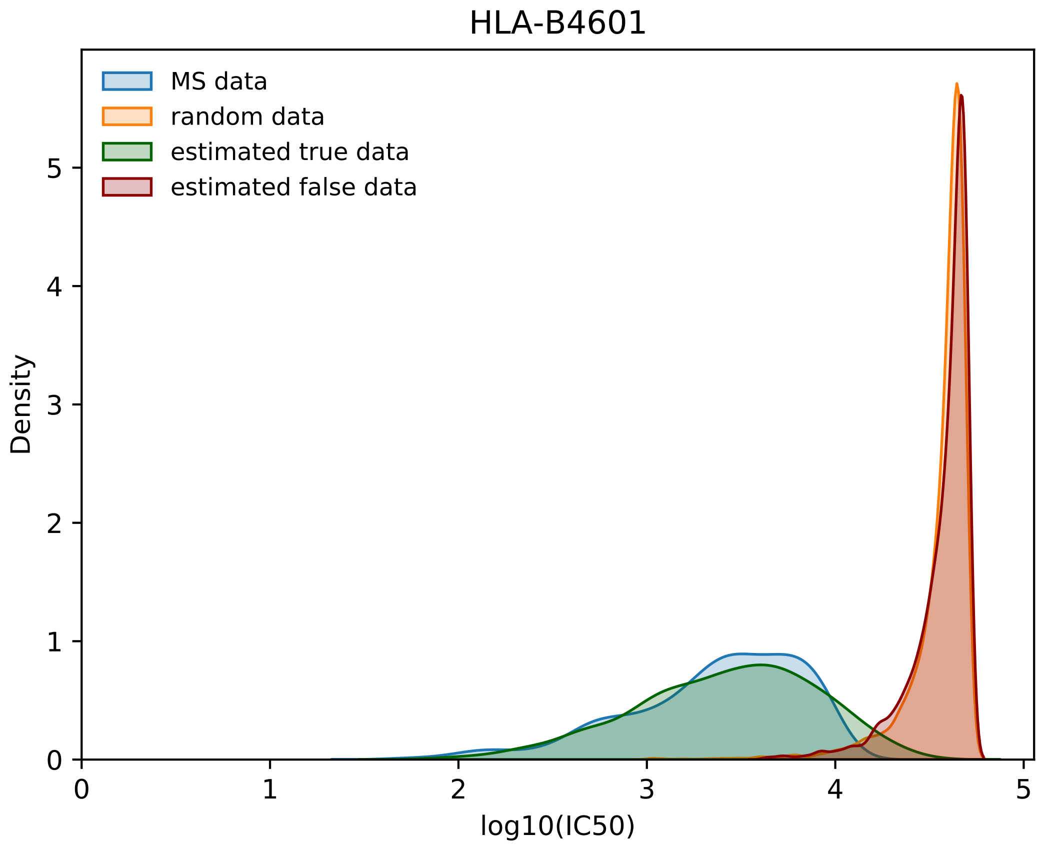
HLA-B4601
MS data
random data
estimated true data
estimated false data
Density
log10(IC50)
0
1
2
3
4
5
0
1
2
3
4
5

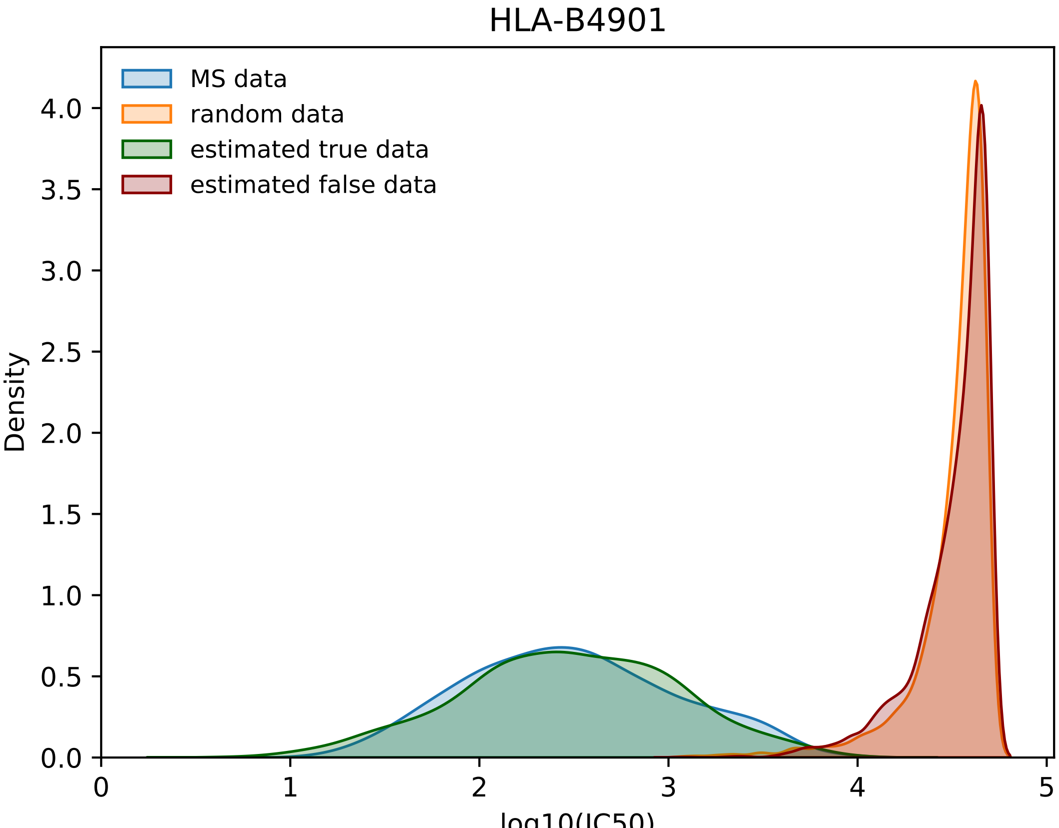

HLA-B4901
MS data
random data
estimated true data
estimated false data
Density
log10(IC50)
0.0
0.5
1.0
1.5
2.0
2.5
3.0
3.5
4.0
0
1
2
3
4
5

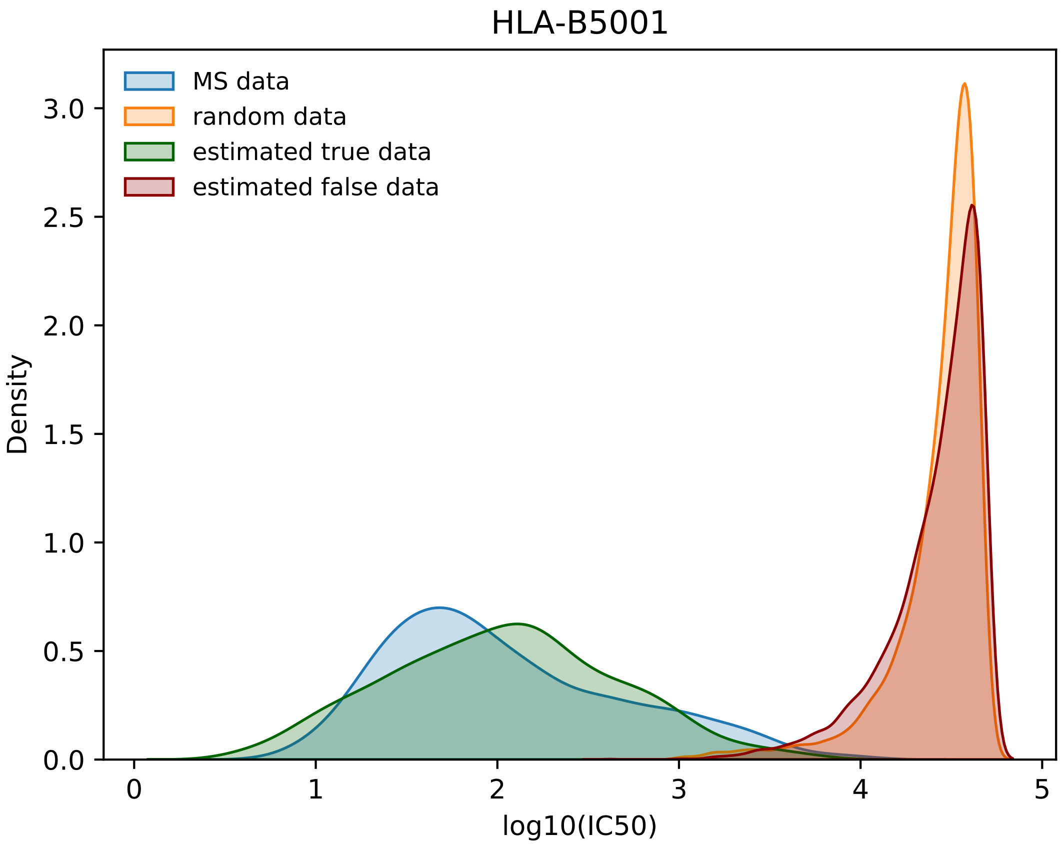
HLA-B5001
MS data
random data
estimated true data
estimated false data
Density
log10(IC50)
0.0
0.5
1.0
1.5
2.0
2.5
3.0
0
1
2
3
4
5

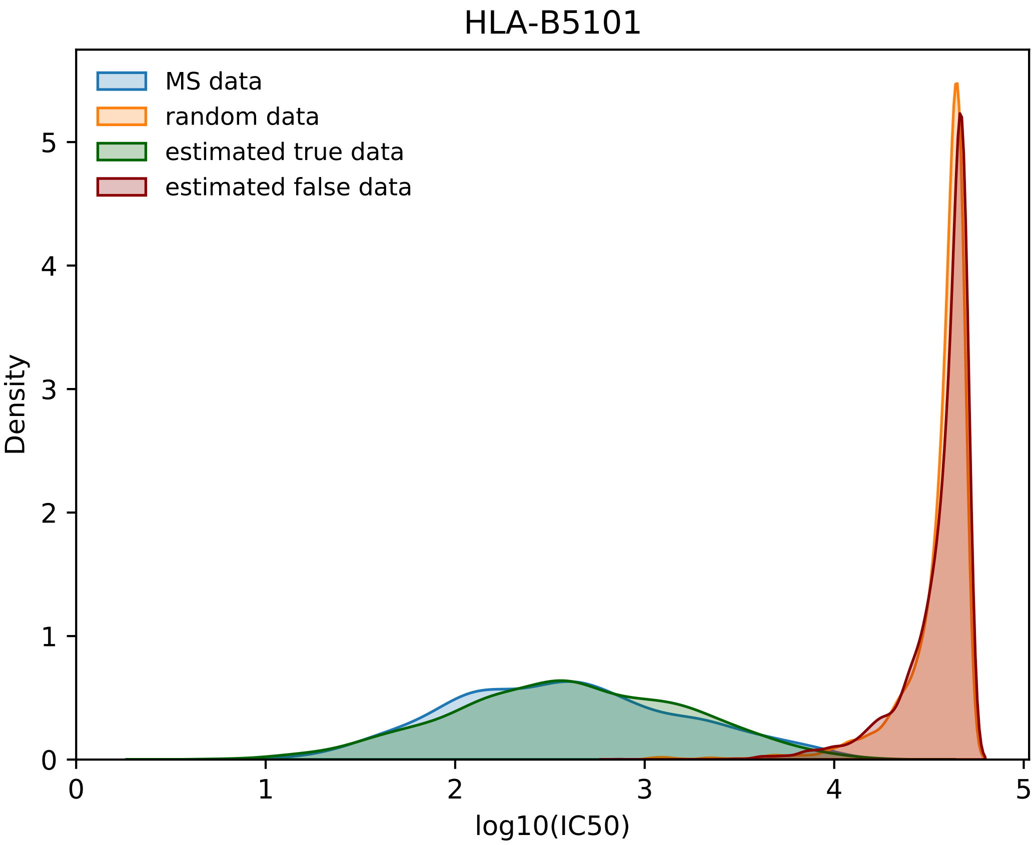
HLA-B5101
MS data
random data
estimated true data
estimated false data
Density
log10(IC50)
0
1
2
3
4
5
0
1
2
3
4
5

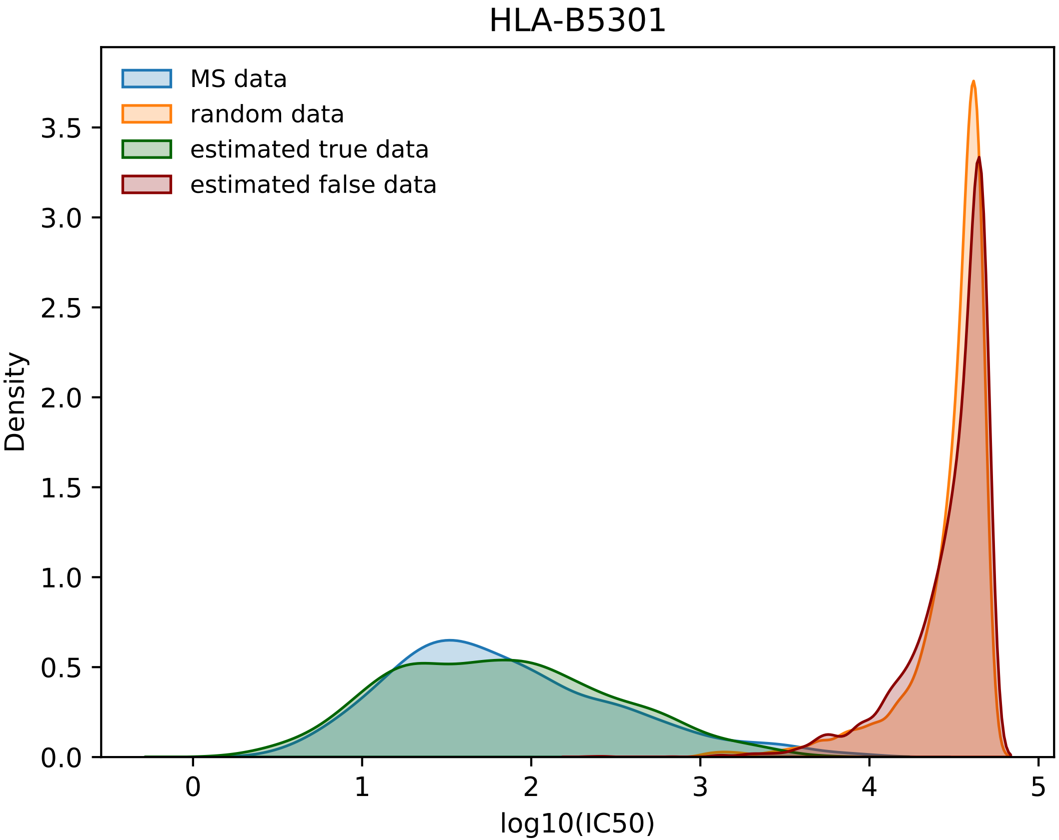
HLA-B5301
MS data
random data
estimated true data
estimated false data
Density
0.0
0.5
1.0
1.5
2.0
2.5
3.0
3.5
0
1
2
3
4
5
log10(IC50)

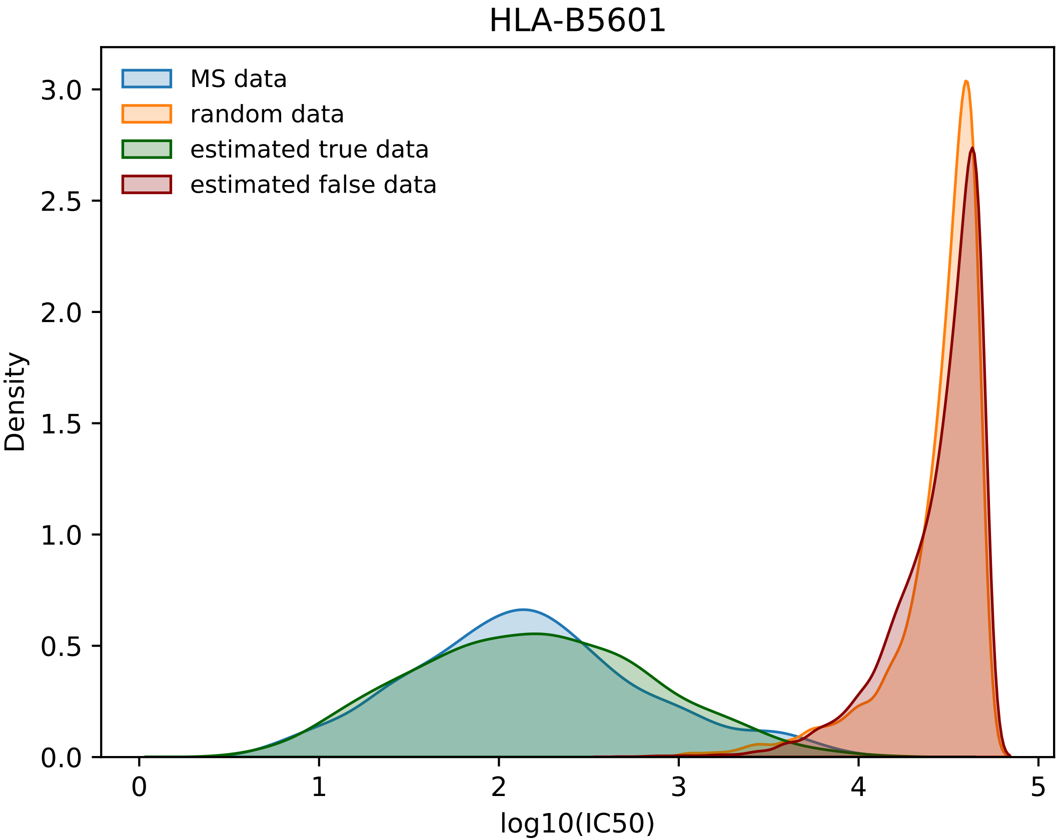

HLA-B5601
MS data
random data
estimated true data
estimated false data
Density
log10(IC50)
0.0
0.5
1.0
1.5
2.0
2.5
3.0
0
1
2
3
4
5

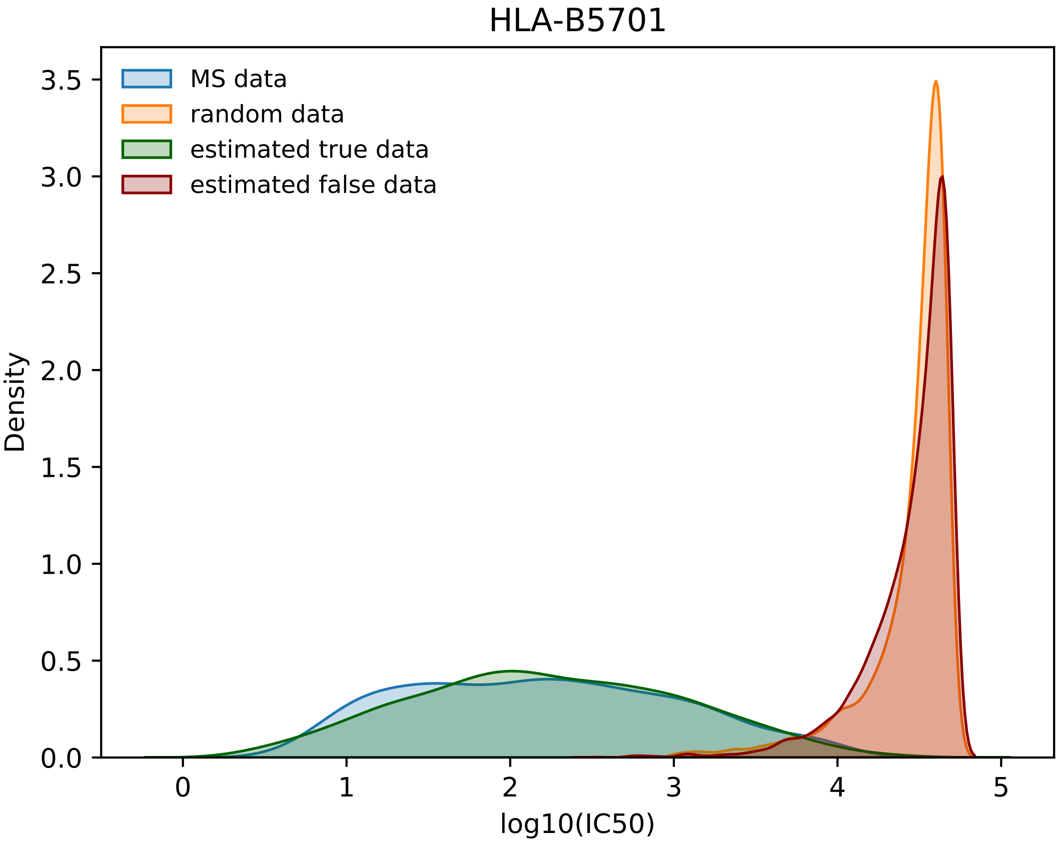

HLA-B5701
MS data
random data
estimated true data
estimated false data
Density
0.0
0.5
1.0
1.5
2.0
2.5
3.0
3.5
0
1
2
3
4
5
log10(IC50)

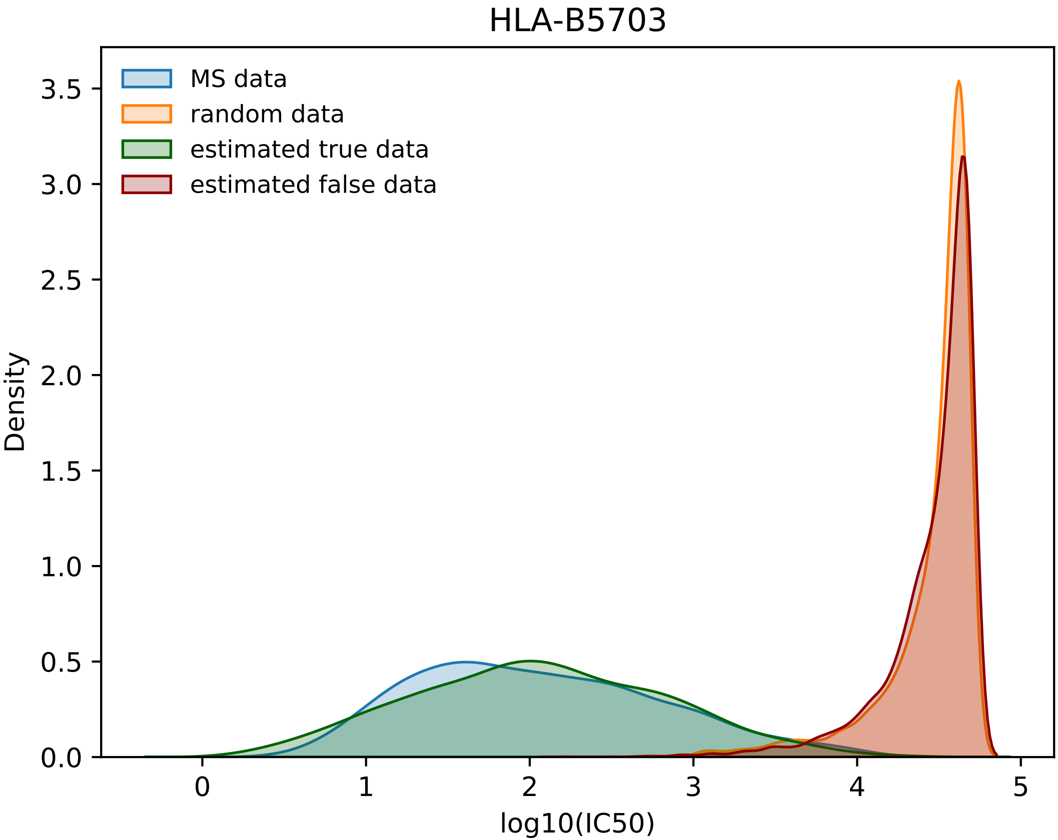
HLA-B5703
MS data
random data
estimated true data
estimated false data
Density
log10(IC50)
0.0
0.5
1.0
1.5
2.0
2.5
3.0
3.5
0
1
2
3
4
5

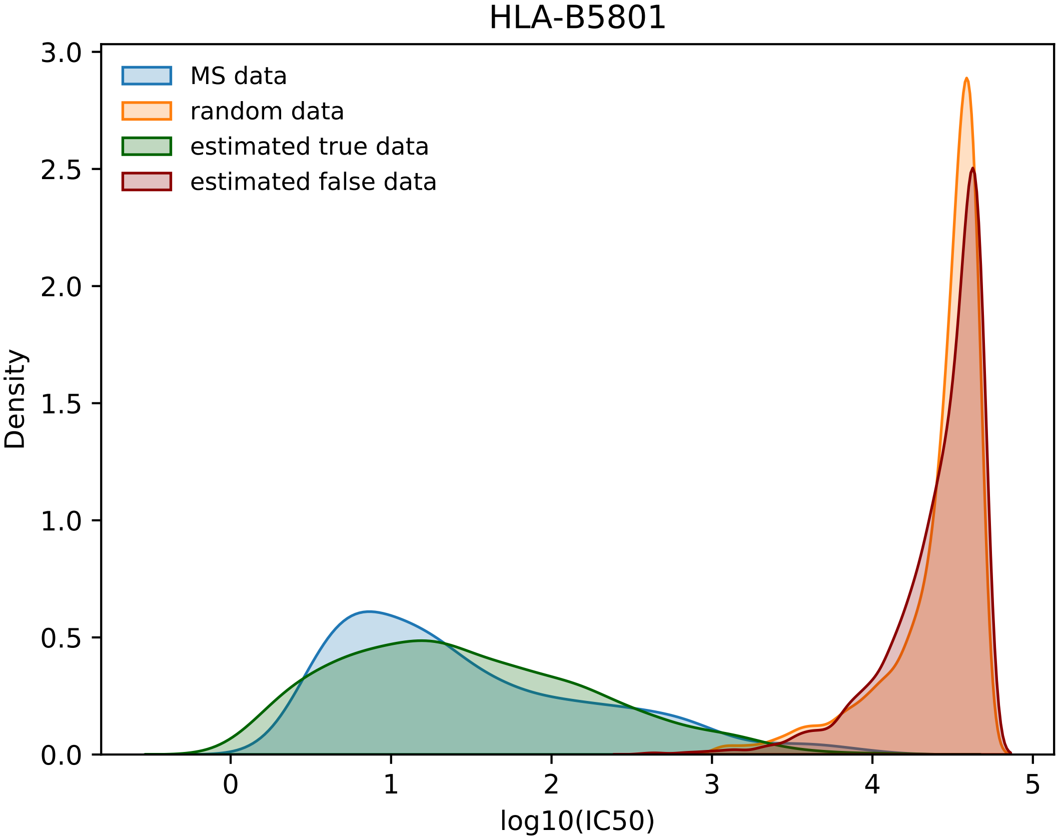
HLA-B5801
MS data
random data
estimated true data
estimated false data
Density
log10(IC50)
0.0
0.5
1.0
1.5
2.0
2.5
3.0
0
1
2
3
4
5

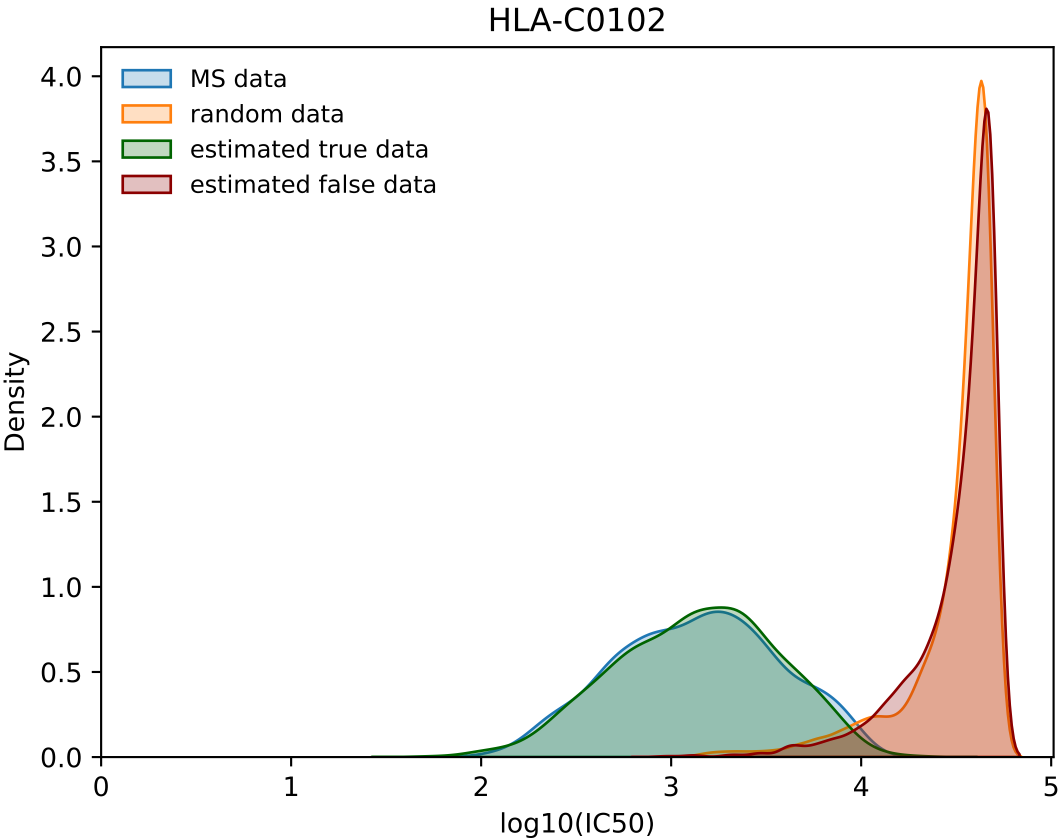
HLA-C0102
MS data
random data
estimated true data
estimated false data
Density
log10(IC50)
0.0
0.5
1.0
1.5
2.0
2.5
3.0
3.5
4.0
0
1
2
3
4
5

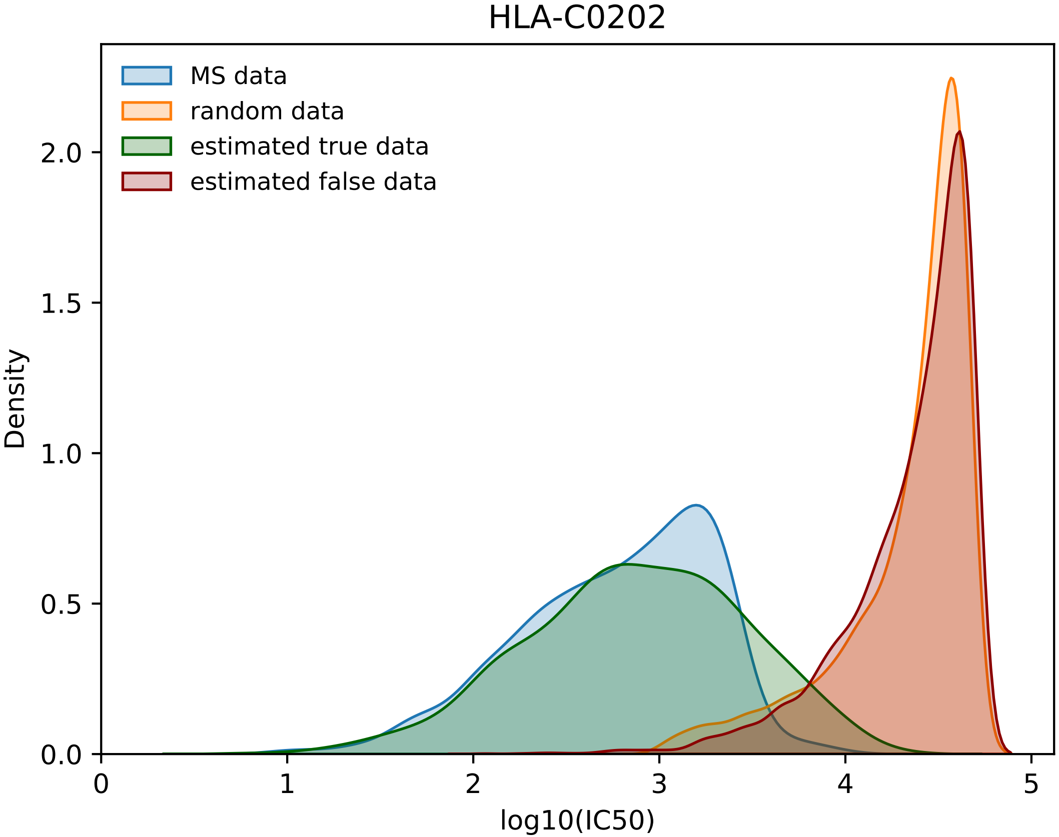
HLA-C0202
MS data
random data
estimated true data
estimated false data
Density
log10(IC50)
0.0
0.5
1.0
1.5
2.0
0
1
2
3
4
5

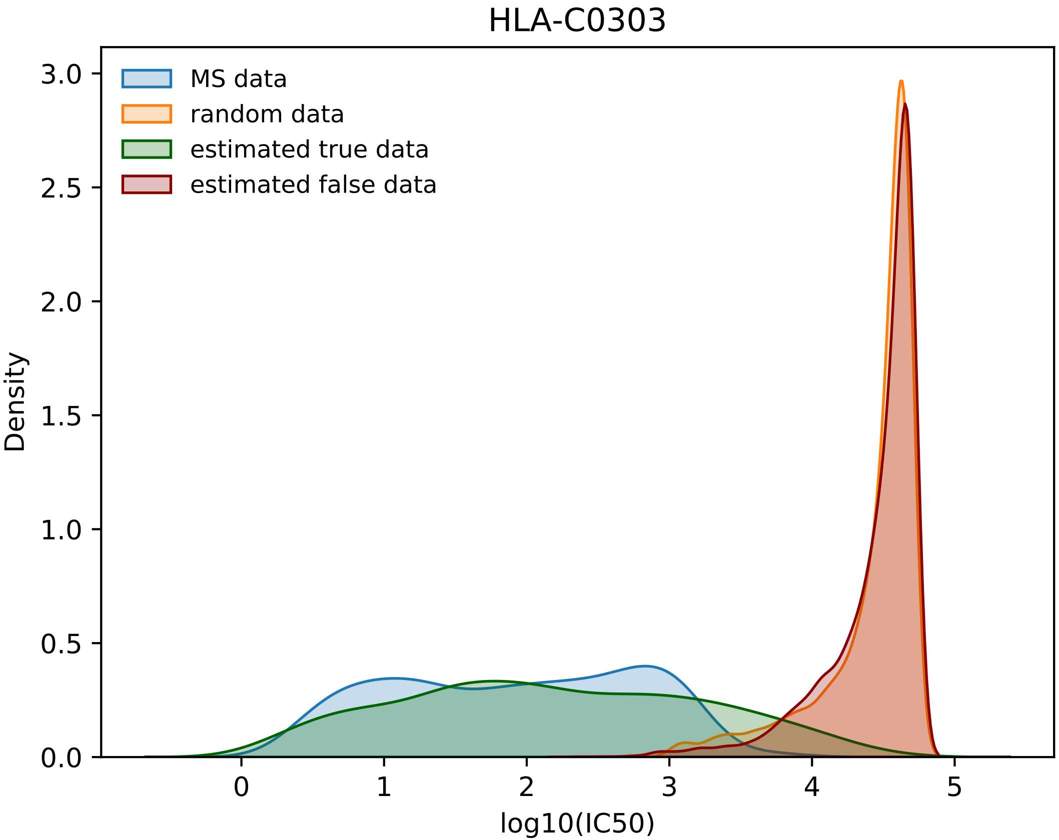

HLA-C0303
MS data
random data
estimated true data
estimated false data
Density
3.0
2.5
2.0
1.5
1.0
0.5
0.0
0
1
2
3
4
5
log10(IC50)

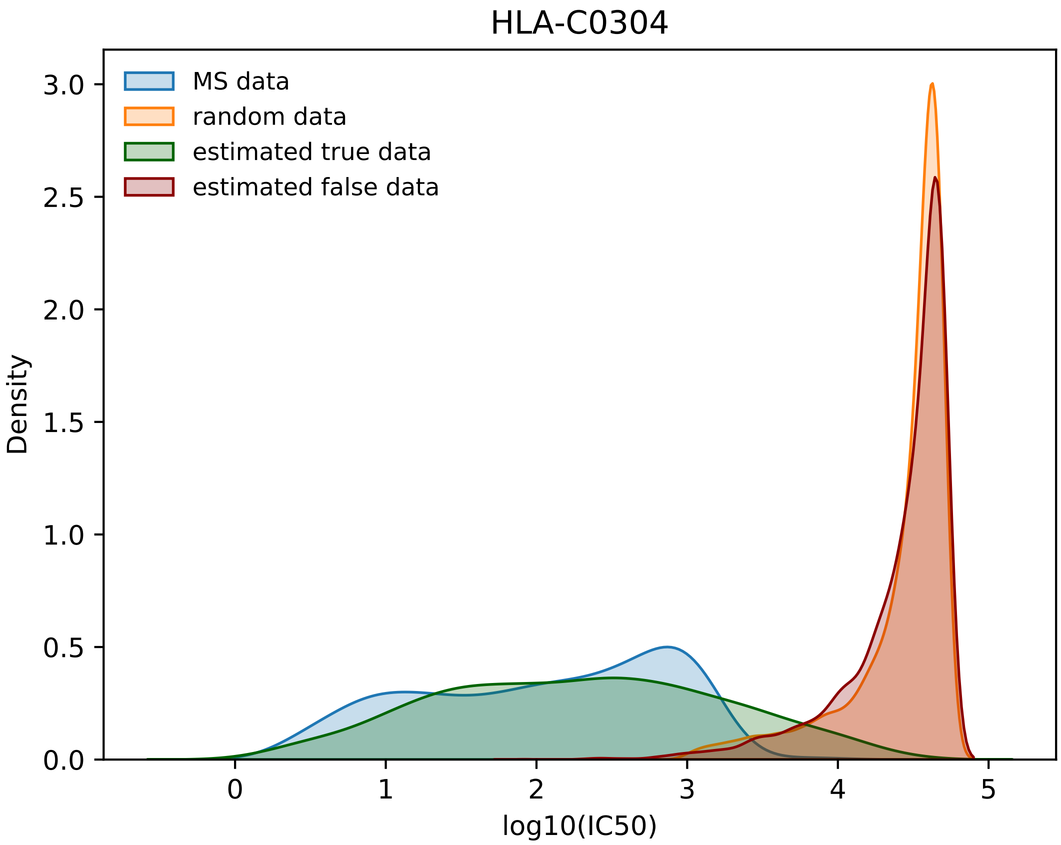
HLA-C0304
MS data
random data
estimated true data
estimated false data
Density
log10(IC50)
3.0
2.5
2.0
1.5
1.0
0.5
0.0
0
1
2
3
4
5

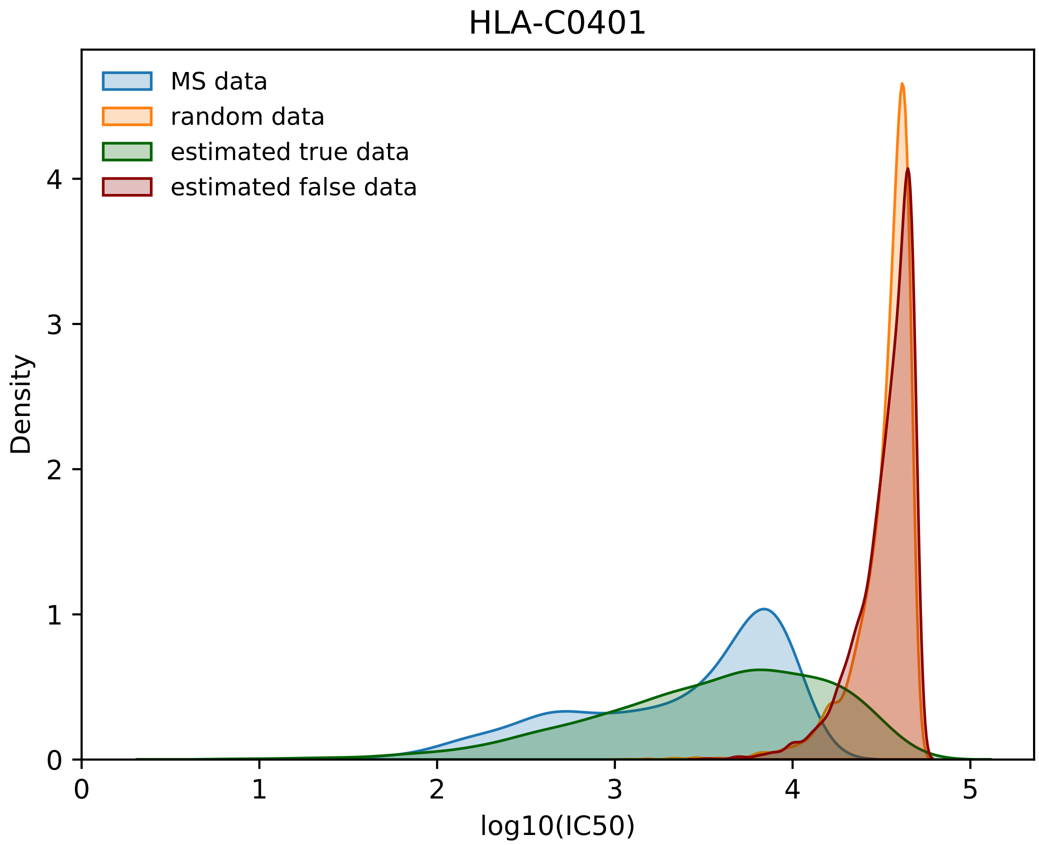

HLA-C0401
MS data
random data
estimated true data
estimated false data
Density
log10(IC50)
0
1
2
3
4
5

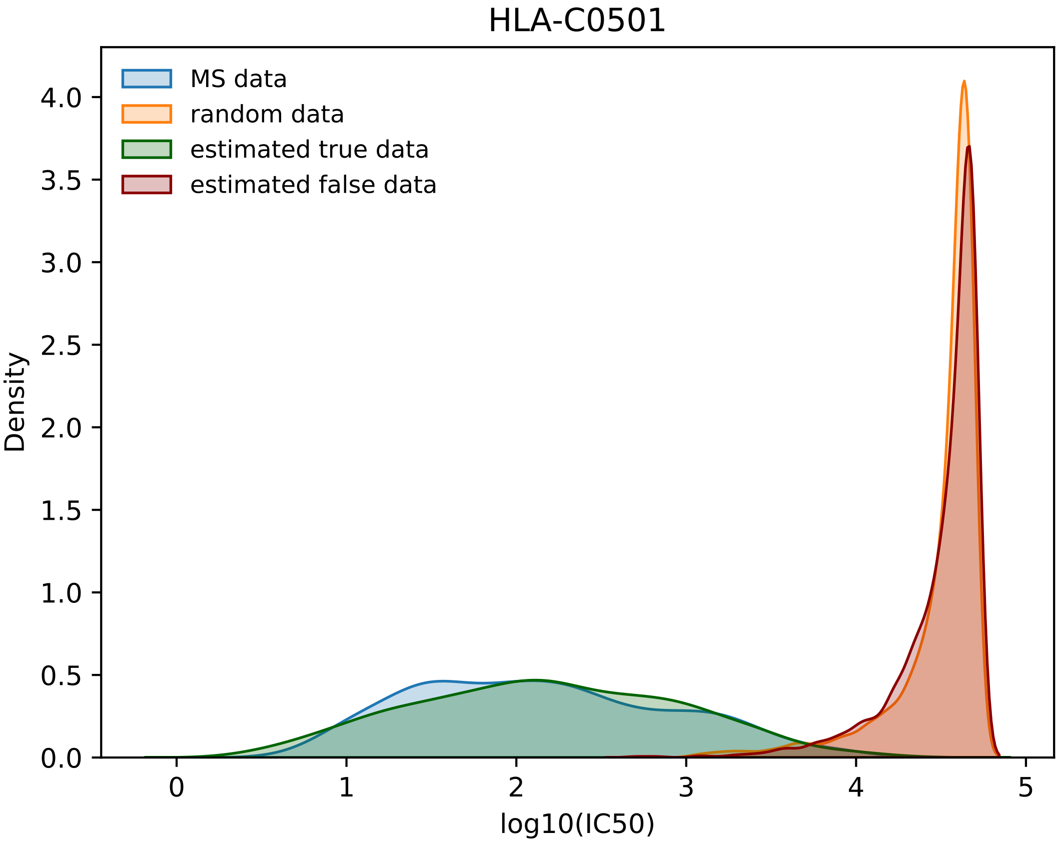
HLA-C0501
MS data
random data
estimated true data
estimated false data
Density
log10(IC50)
0.0
0.5
1.0
1.5
2.0
2.5
3.0
3.5
4.0
0
1
2
3
4
5

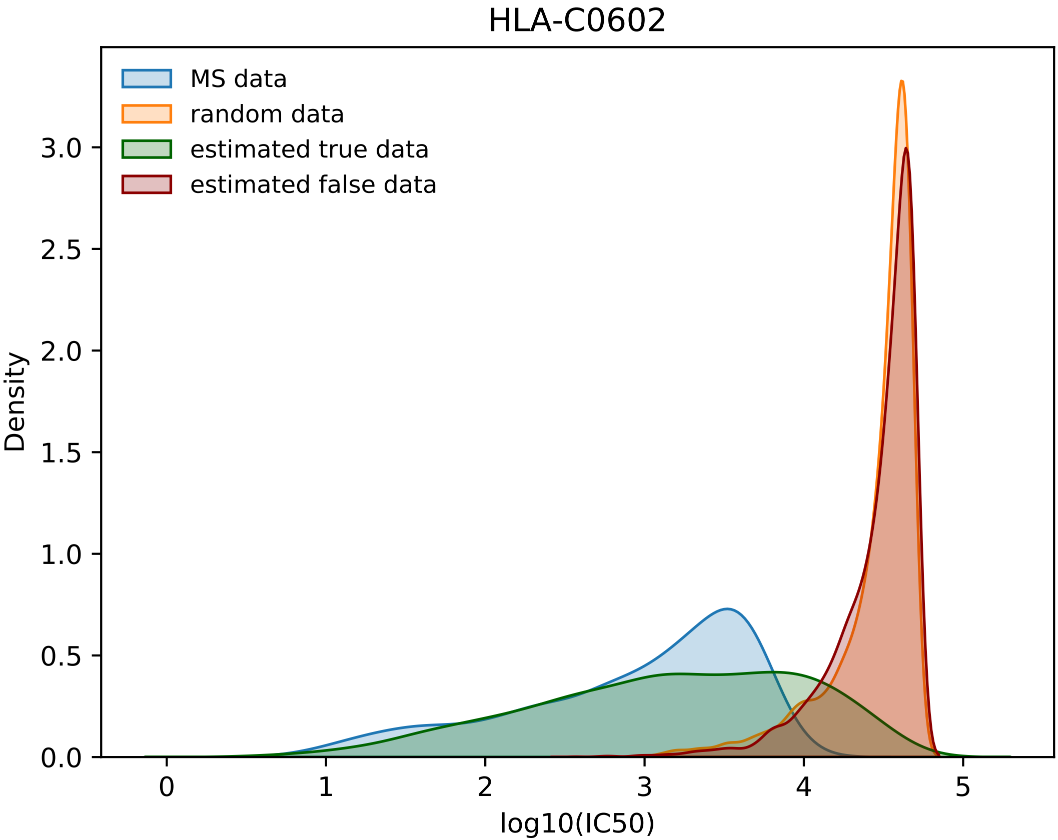

HLA-C0602
MS data
random data
estimated true data
estimated false data
Density
log10(IC50)
0.0
0.5
1.0
1.5
2.0
2.5
3.0
0
1
2
3
4
5

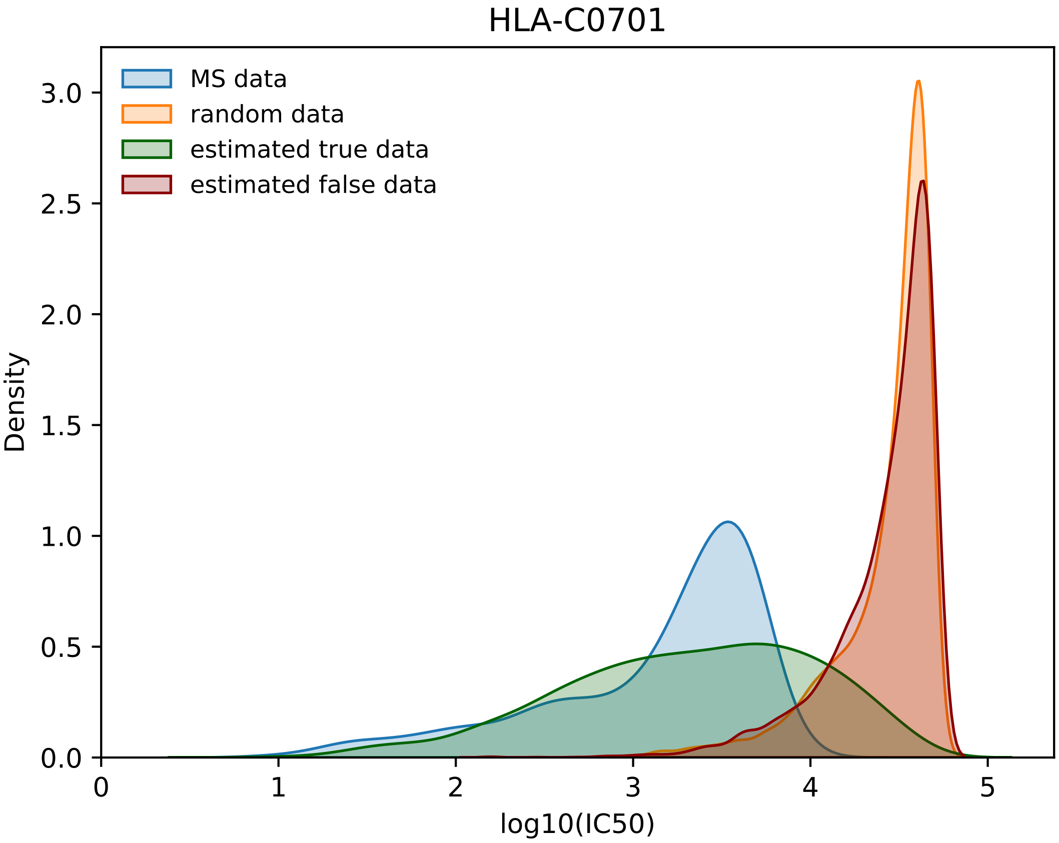
HLA-C0701
MS data
random data
estimated true data
estimated false data
Density
log10(IC50)
0.0
0.5
1.0
1.5
2.0
2.5
3.0
0
1
2
3
4
5

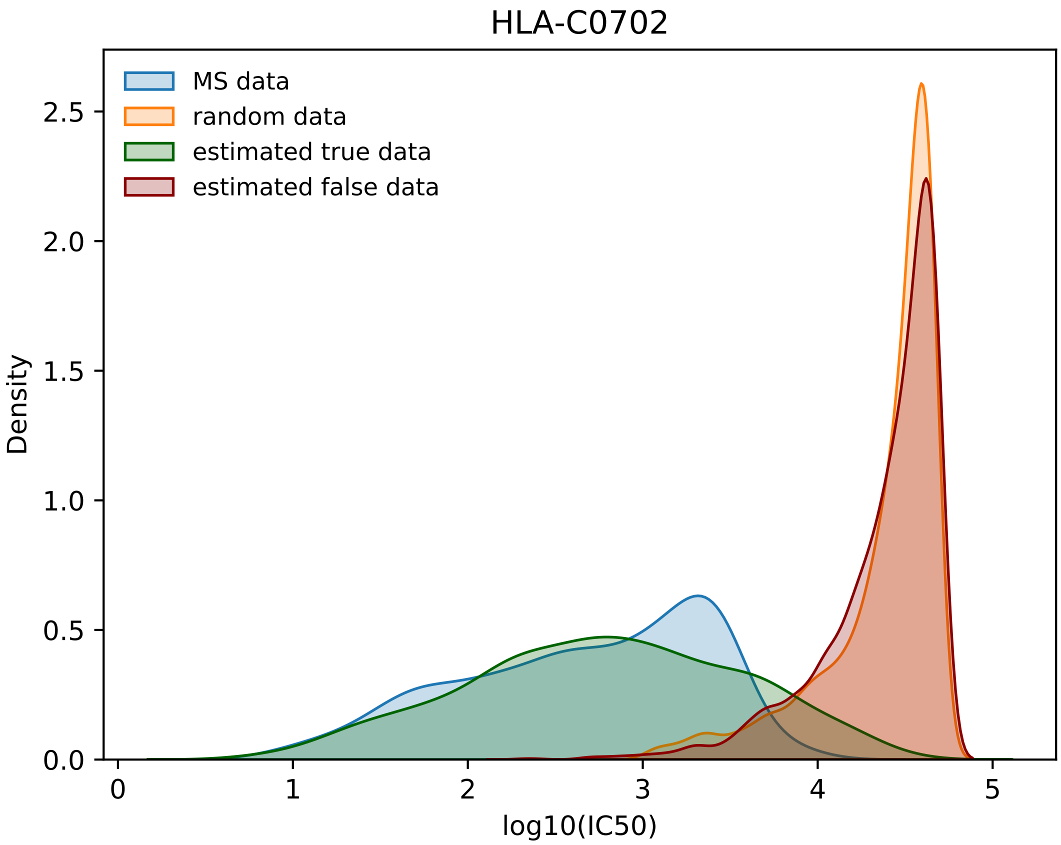
HLA-C0702
MS data
random data
estimated true data
estimated false data
Density
0.0
0.5
1.0
1.5
2.0
2.5
0
1
2
3
4
5
log10(IC50)

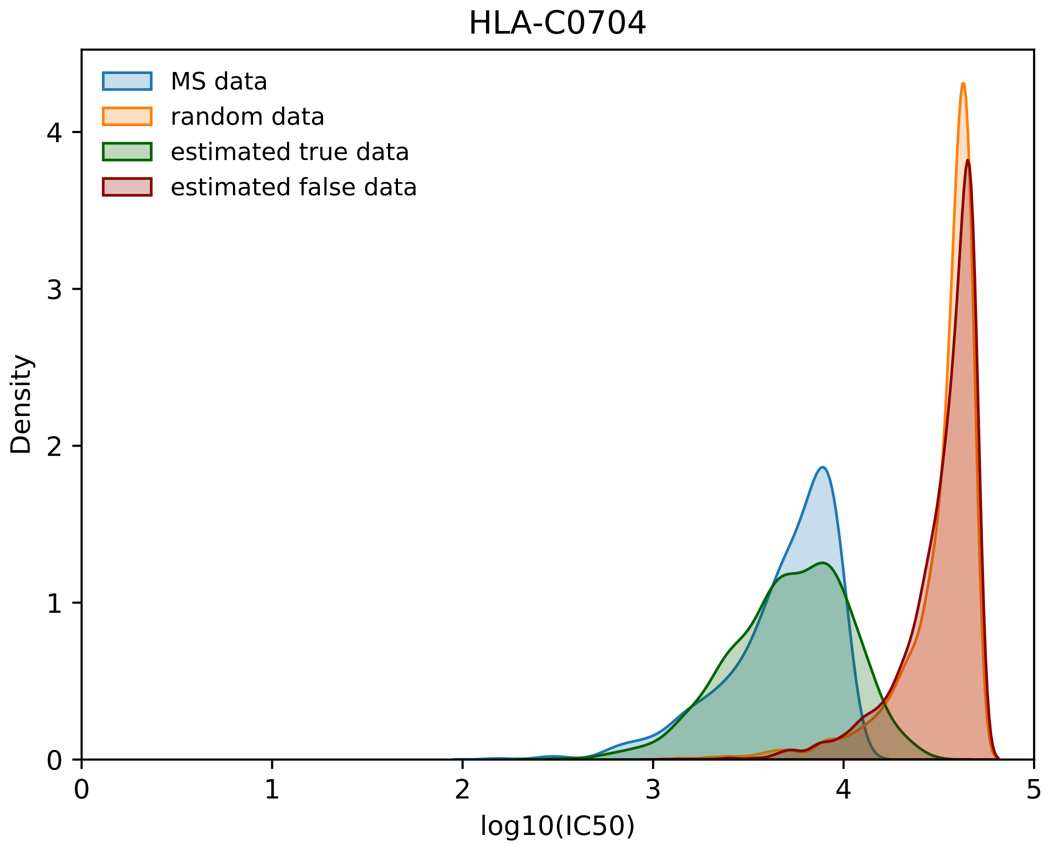

HLA-C0704
MS data
random data
estimated true data
estimated false data
Density
log10(IC50)
0
1
2
3
4
5

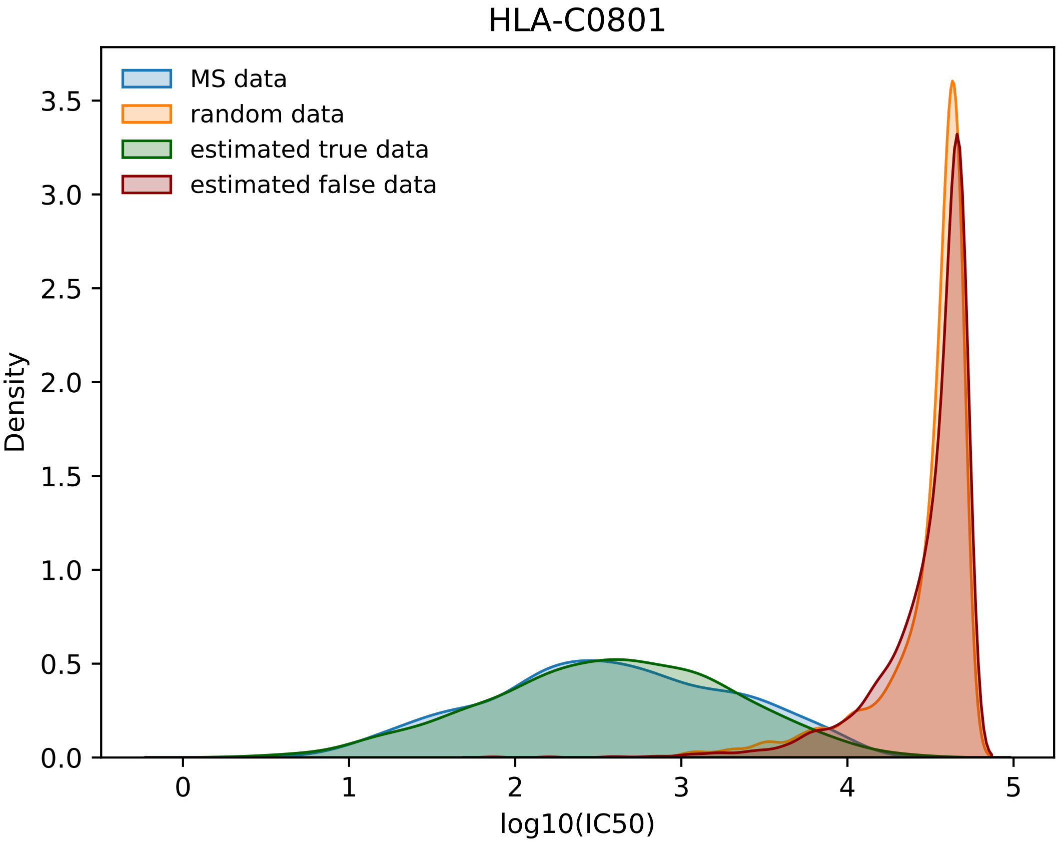
HLA-C0801
MS data
random data
estimated true data
estimated false data
Density
log10(IC50)
0.0
0.5
1.0
1.5
2.0
2.5
3.0
3.5
0
1
2
3
4
5

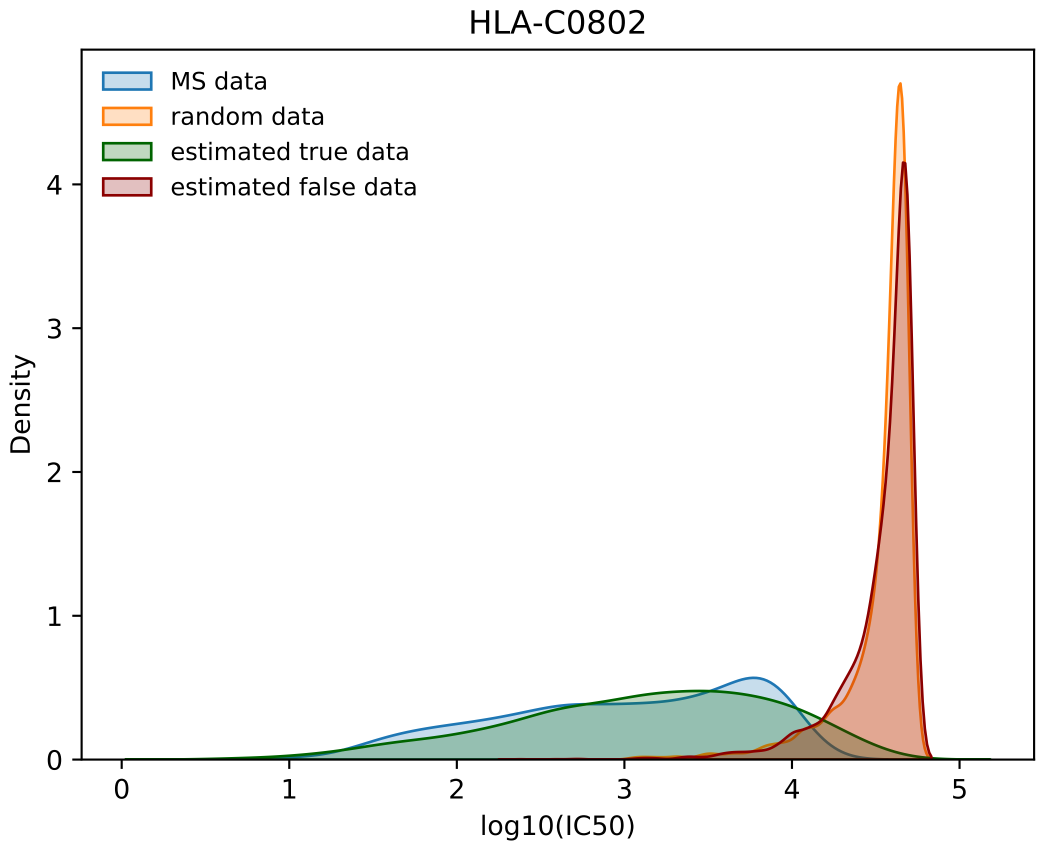

HLA-C0802
MS data
random data
estimated true data
estimated false data
Density
log10(IC50)
0
1
2
3
4
5

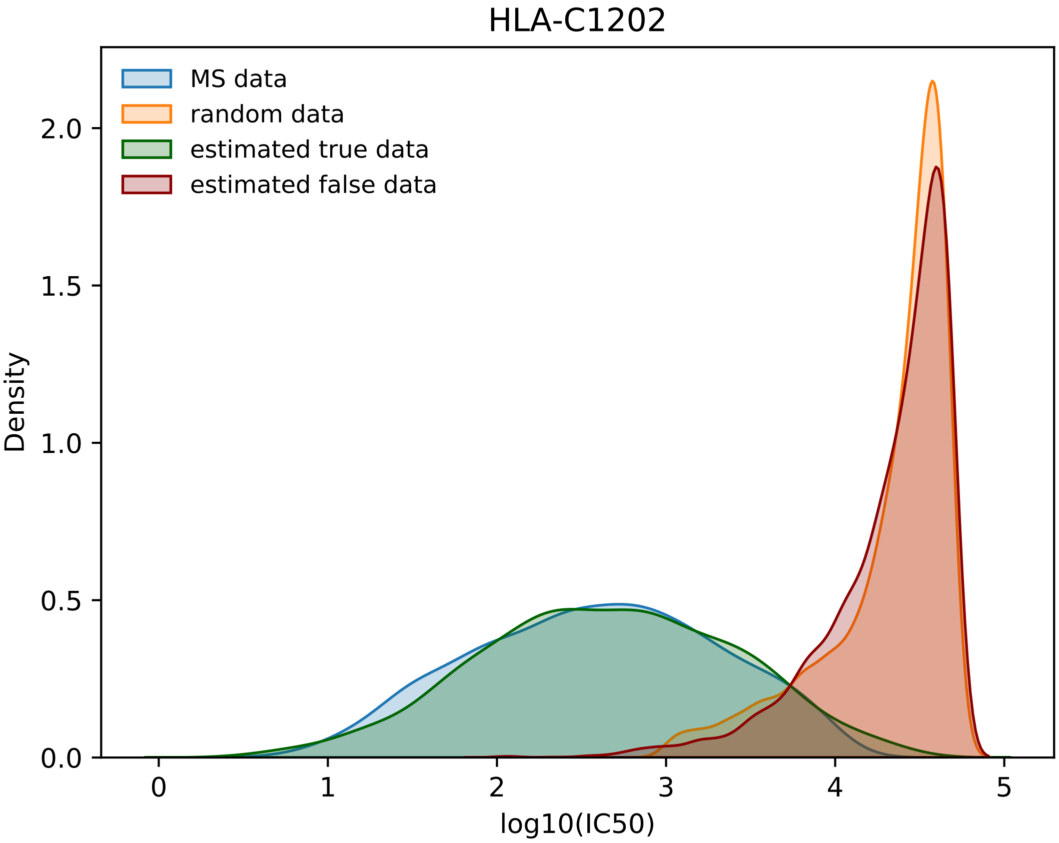

HLA-C1202
MS data
random data
estimated true data
estimated false data
Density
log10(IC50)
0.0
0.5
1.0
1.5
2.0
0
1
2
3
4
5

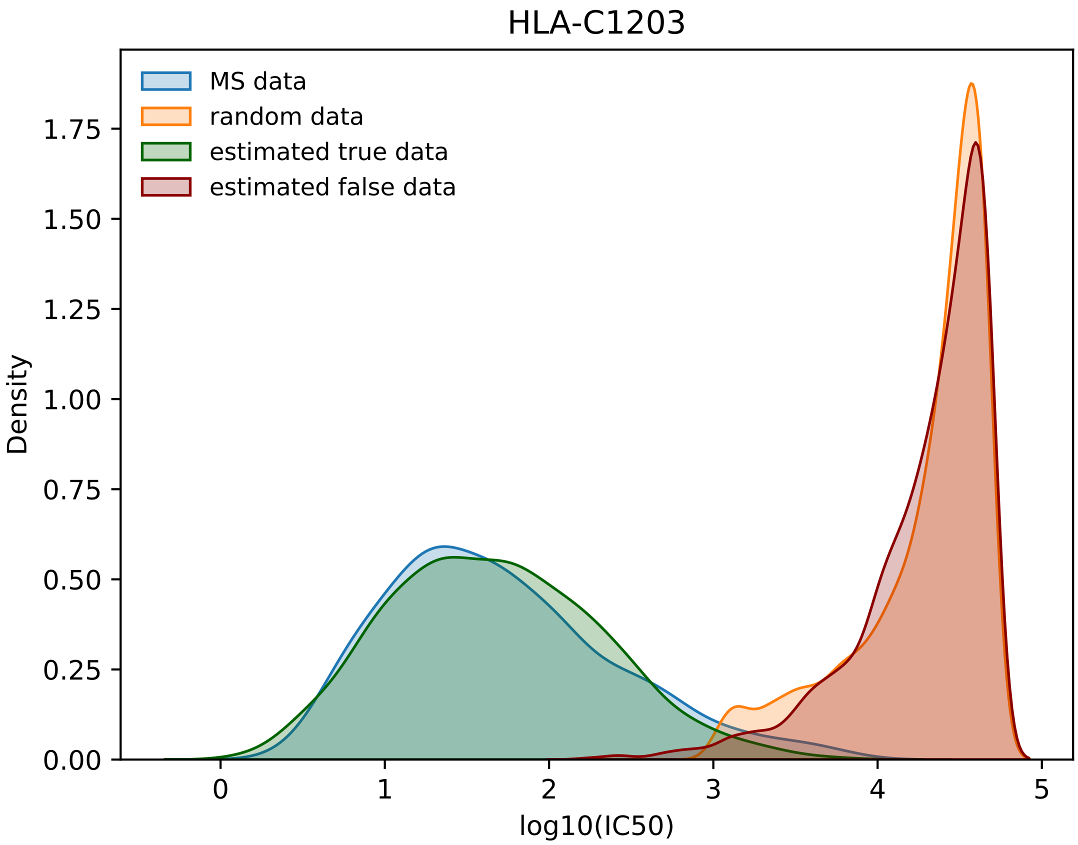

HLA-C1203
MS data
random data
estimated true data
estimated false data
Density
log10(IC50)
0.00
0.25
0.50
0.75
1.00
1.25
1.50
1.75
0
1
2
3
4
5

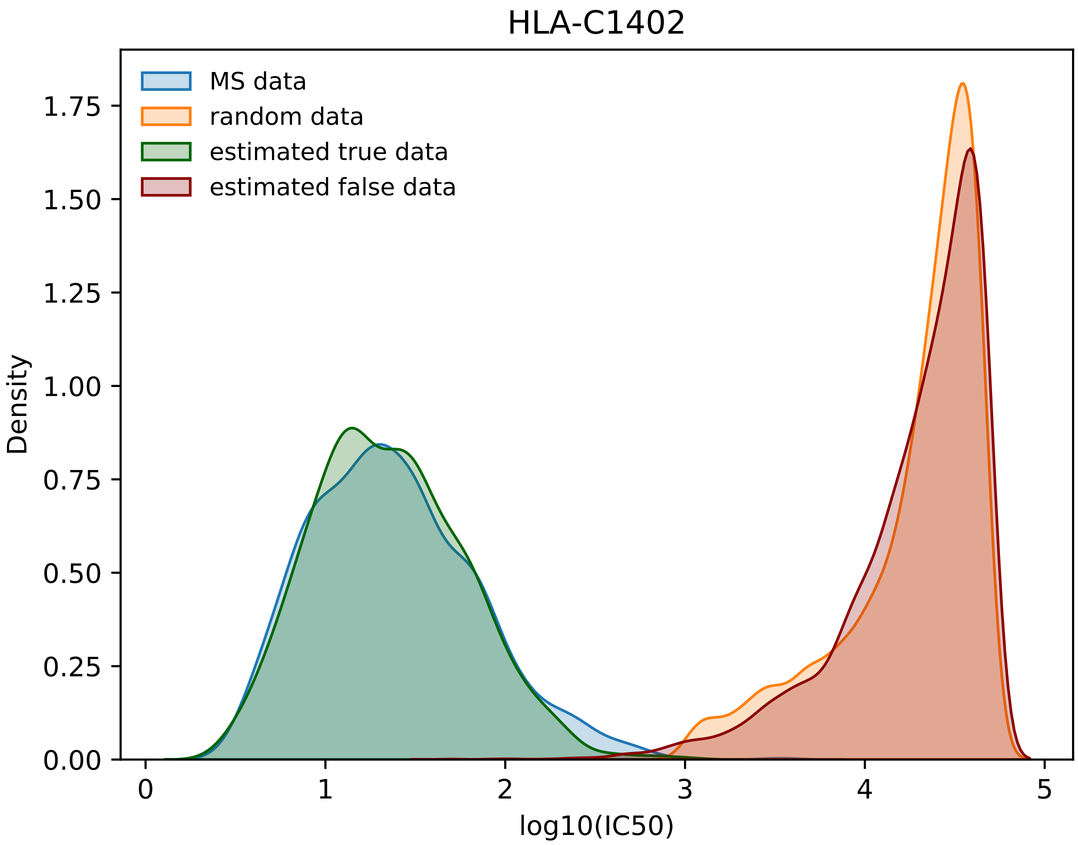

HLA-C1402
MS data
random data
estimated true data
estimated false data
Density
0.00
0.25
0.50
0.75
1.00
1.25
1.50
1.75
0
1
2
3
4
5
log10(IC50)

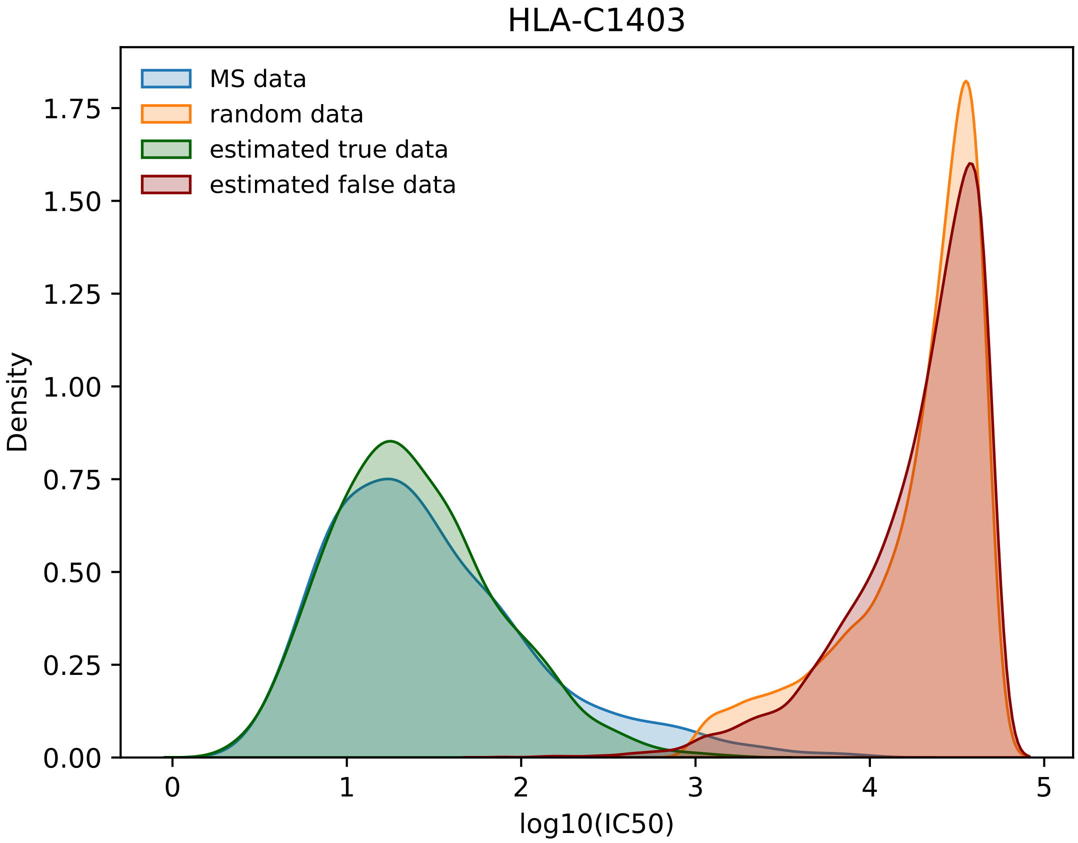

HLA-C1403
MS data
random data
estimated true data
estimated false data
Density
0.00
0.25
0.50
0.75
1.00
1.25
1.50
1.75
0
1
2
3
4
5
log10(IC50)

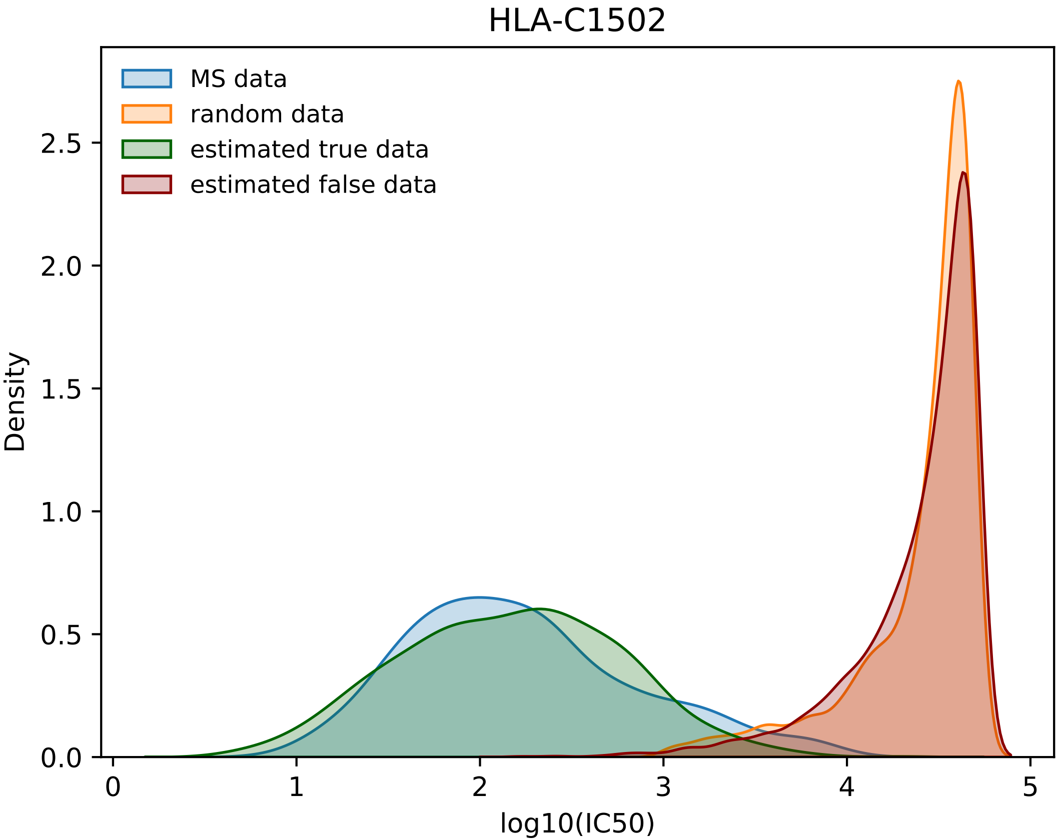

HLA-C1502
MS data
random data
estimated true data
estimated false data
Density
0.0
0.5
1.0
1.5
2.0
2.5
0
1
2
3
4
5
log10(IC50)

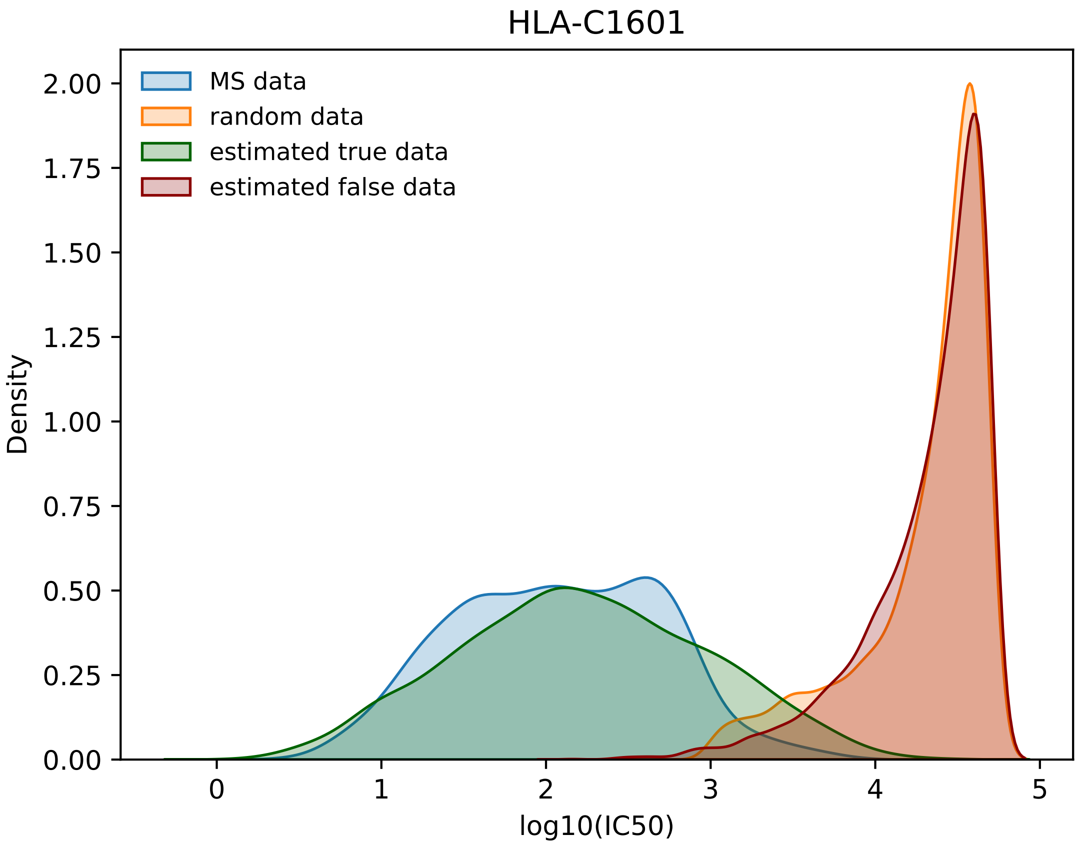
HLA-C1601
MS data
random data
estimated true data
estimated false data
Density
log10(IC50)
0.00
0.25
0.50
0.75
1.00
1.25
1.50
1.75
2.00
0
1
2
3
4
5

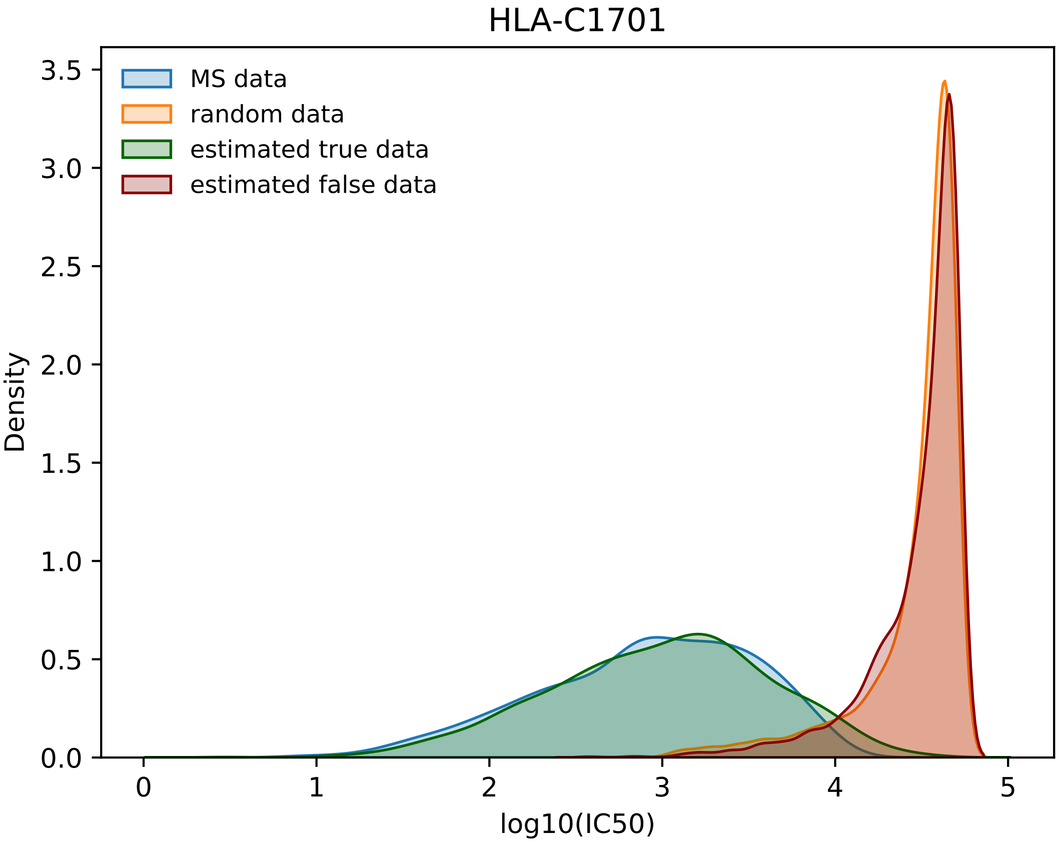
HLA-C1701
MS data
random data
estimated true data
estimated false data
Density
log10(IC50)
0.0
0.5
1.0
1.5
2.0
2.5
3.0
3.5
0
1
2
3
4
5
