# Supplementary material for: MHCVision: estimation of global and local false discovery rate for MHC class I peptide binding prediction
Source: Bioinformatics. 2021 Jul 1;37(21):3830–8. doi: 10.1093/bioinformatics/btab479 (PMC8570816; doi:10.1093/bioinformatics/btab479)
Supplement: btab479_Supplementary_Data [file btab479_supplementary_data.zip › supplementary_data3.pdf]

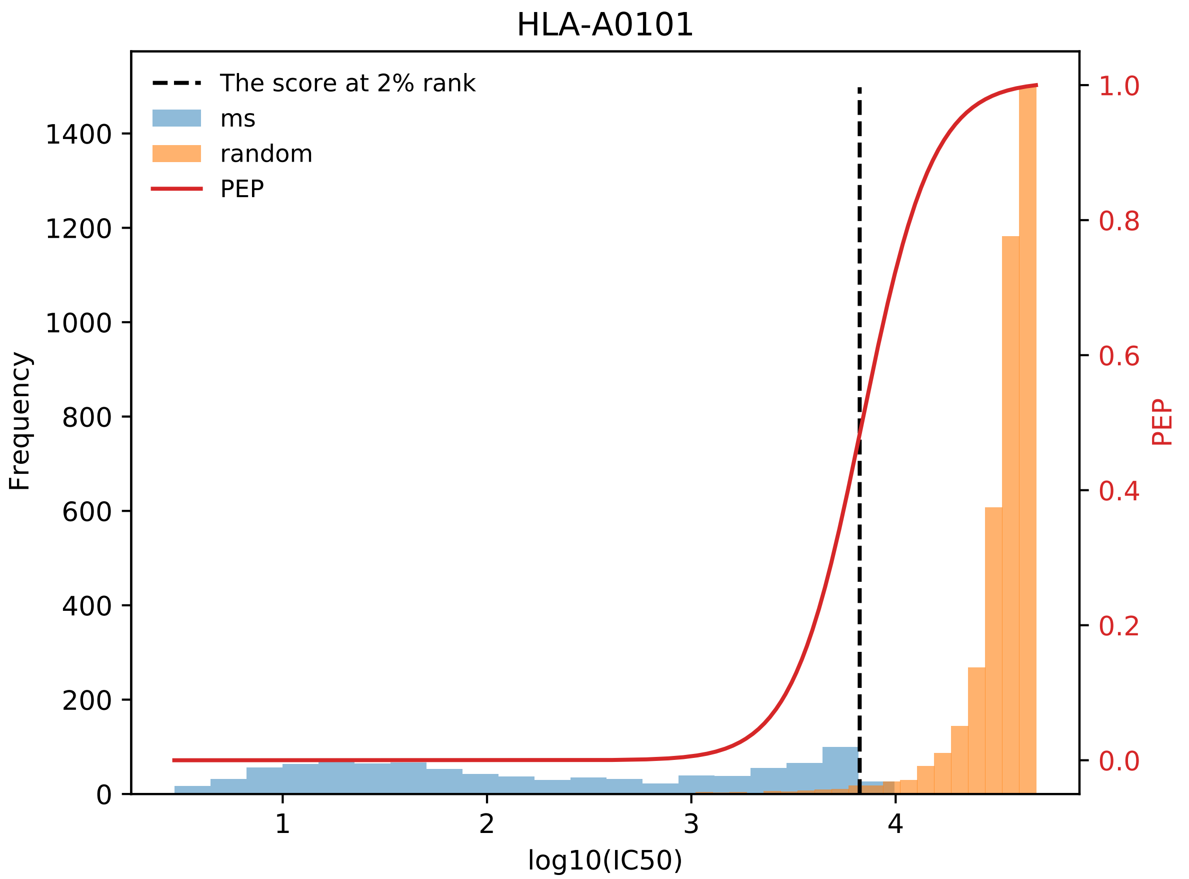
HLA-A0101
The score at 2% rank
ms
random
PEP
Frequency
0
200
400
600
800
1000
1200
1400
1
2
3
4
log10(IC50)
0.0
0.2
0.4
0.6
0.8
1.0
PEP

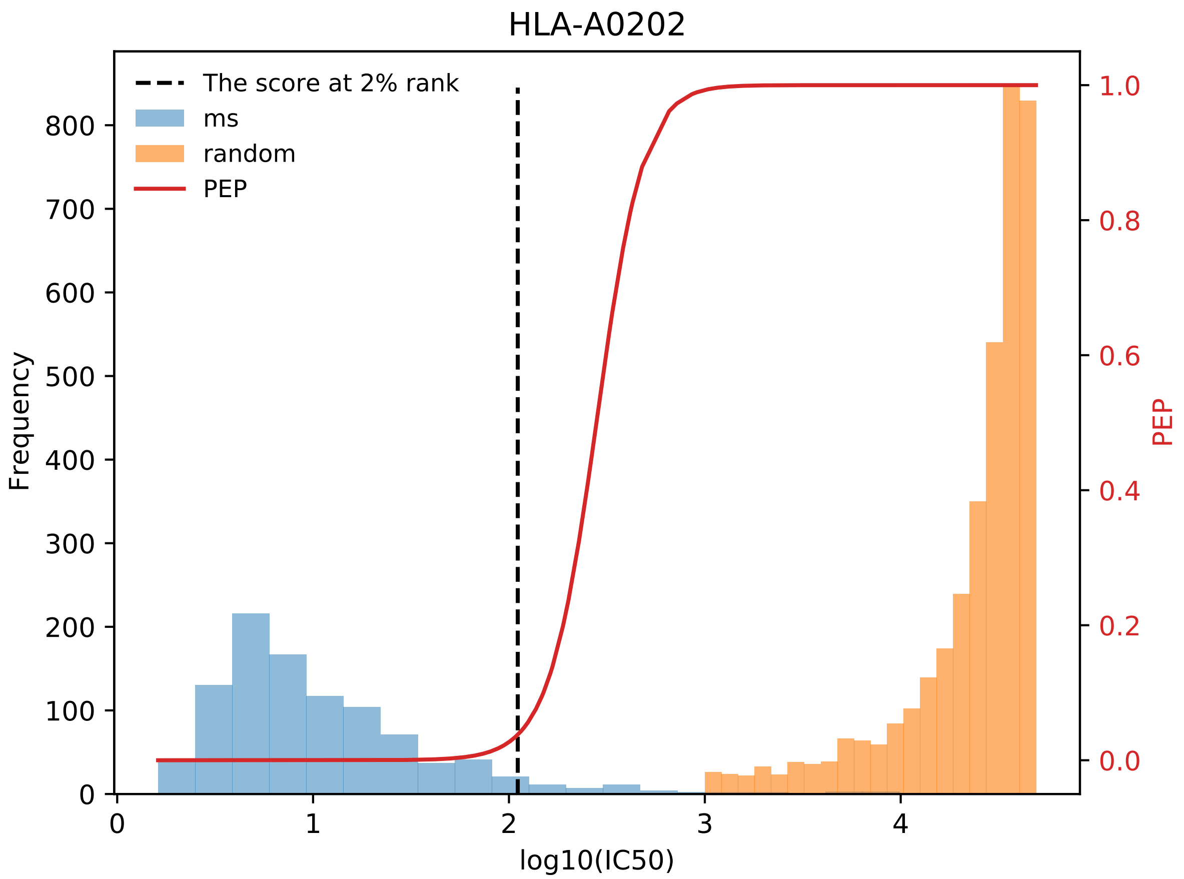

HLA-A0202
The score at 2% rank
ms
random
PEP
Frequency
0
100
200
300
400
500
600
700
800
0
1
2
3
4
log10(IC50)
0.0
0.2
0.4
0.6
0.8
1.0
PEP

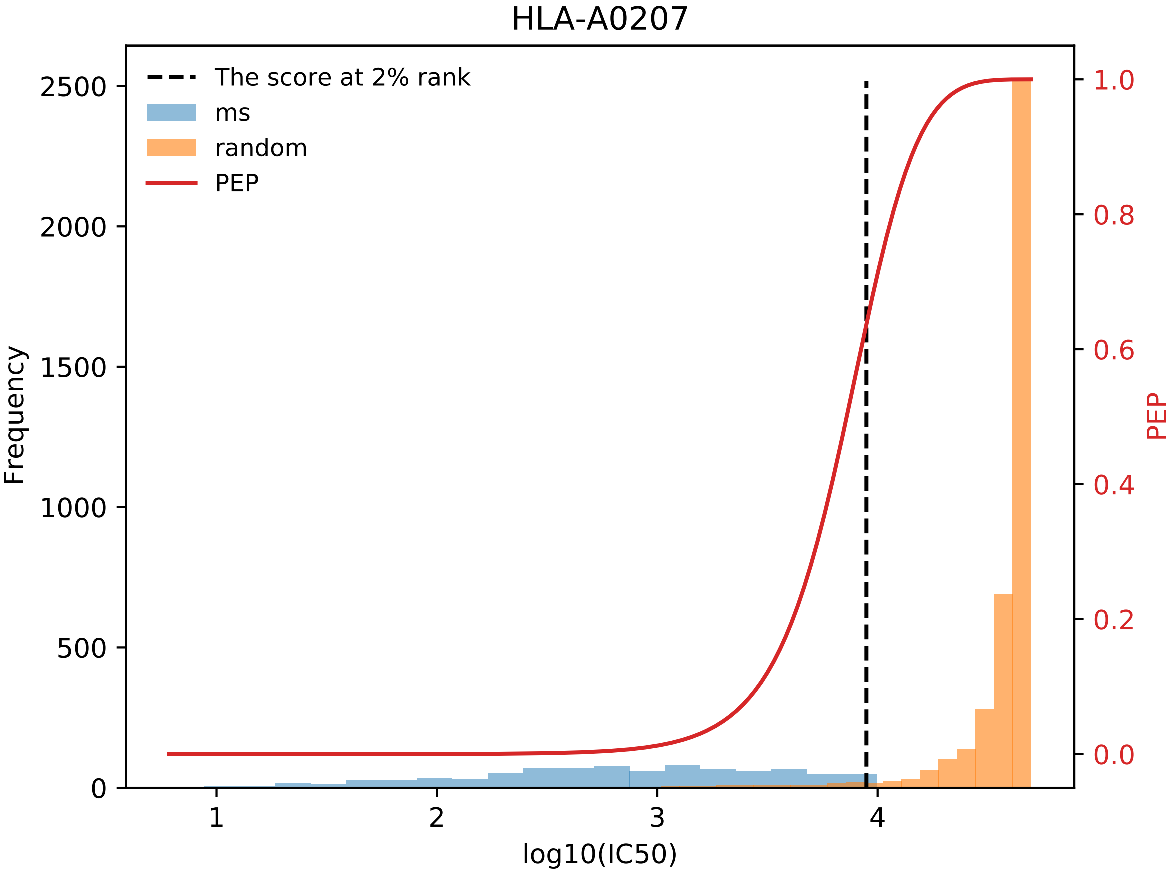
HLA-A0207
The score at 2% rank
ms
random
PEP
Frequency
2500
2000
1500
1000
500
0
1
2
3
4
log10(IC50)
1.0
0.8
0.6
0.4
0.2
0.0
PEP

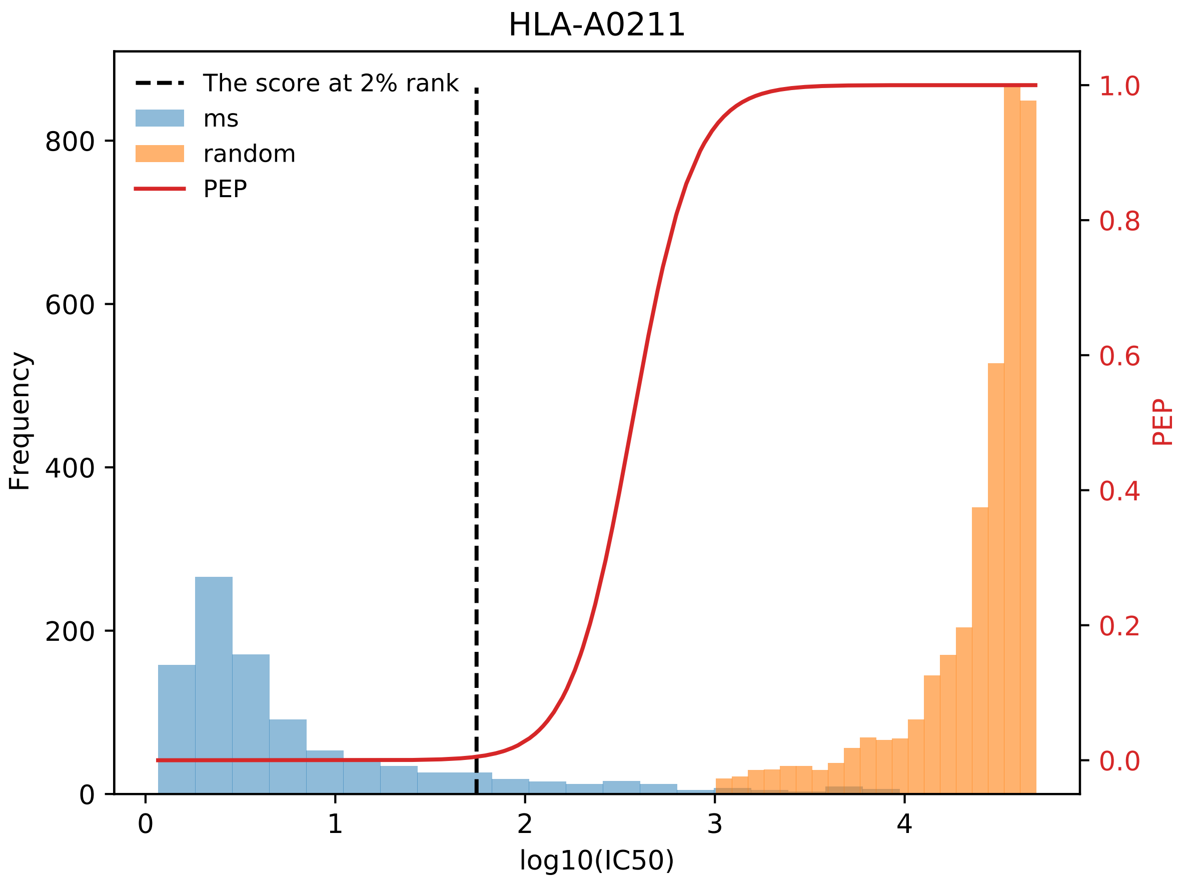

HLA-A0211
The score at 2% rank
ms
random
PEP
Frequency
0
200
400
600
800
0
1
2
3
4
log10(IC50)
0.0
0.2
0.4
0.6
0.8
1.0
PEP

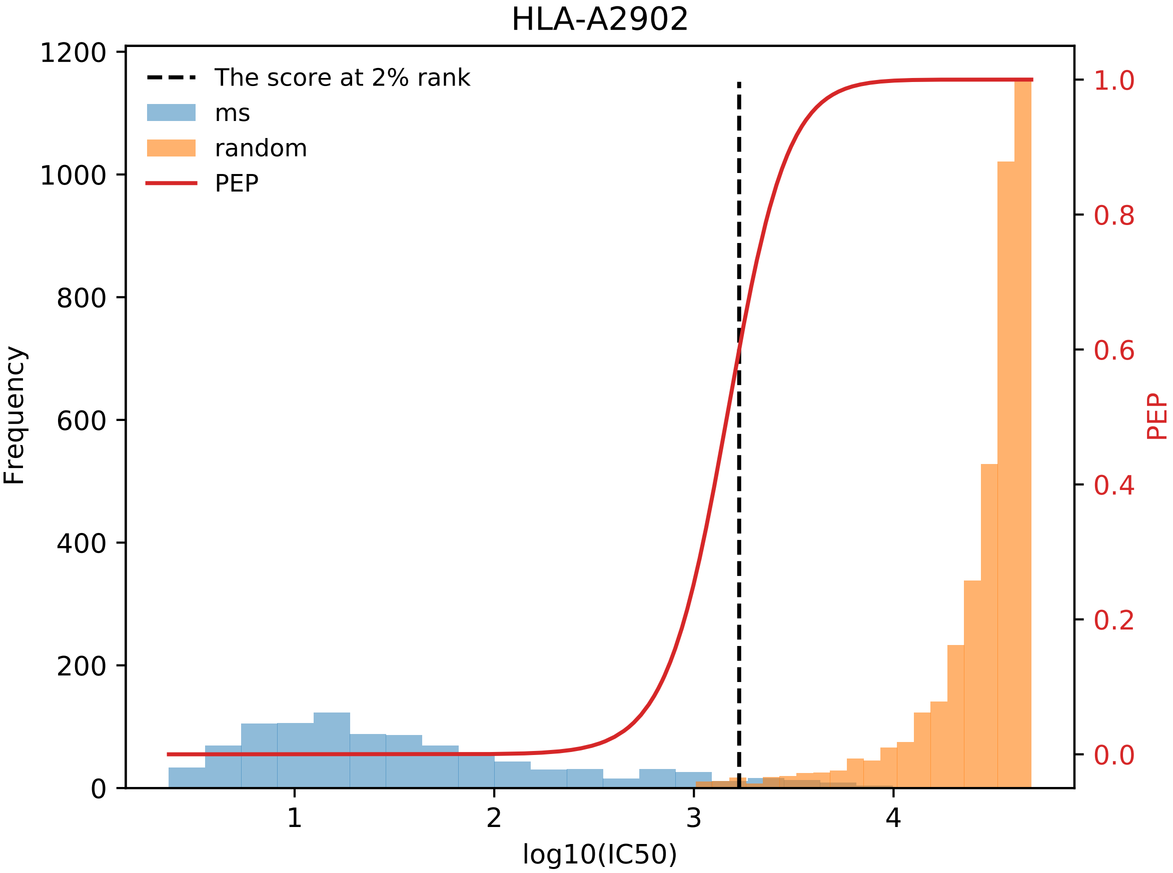
HLA-A2902
The score at 2% rank
ms
random
PEP
Frequency
log10(IC50)
0
200
400
600
800
1000
1200
1
2
3
4
0.0
0.2
0.4
0.6
0.8
1.0

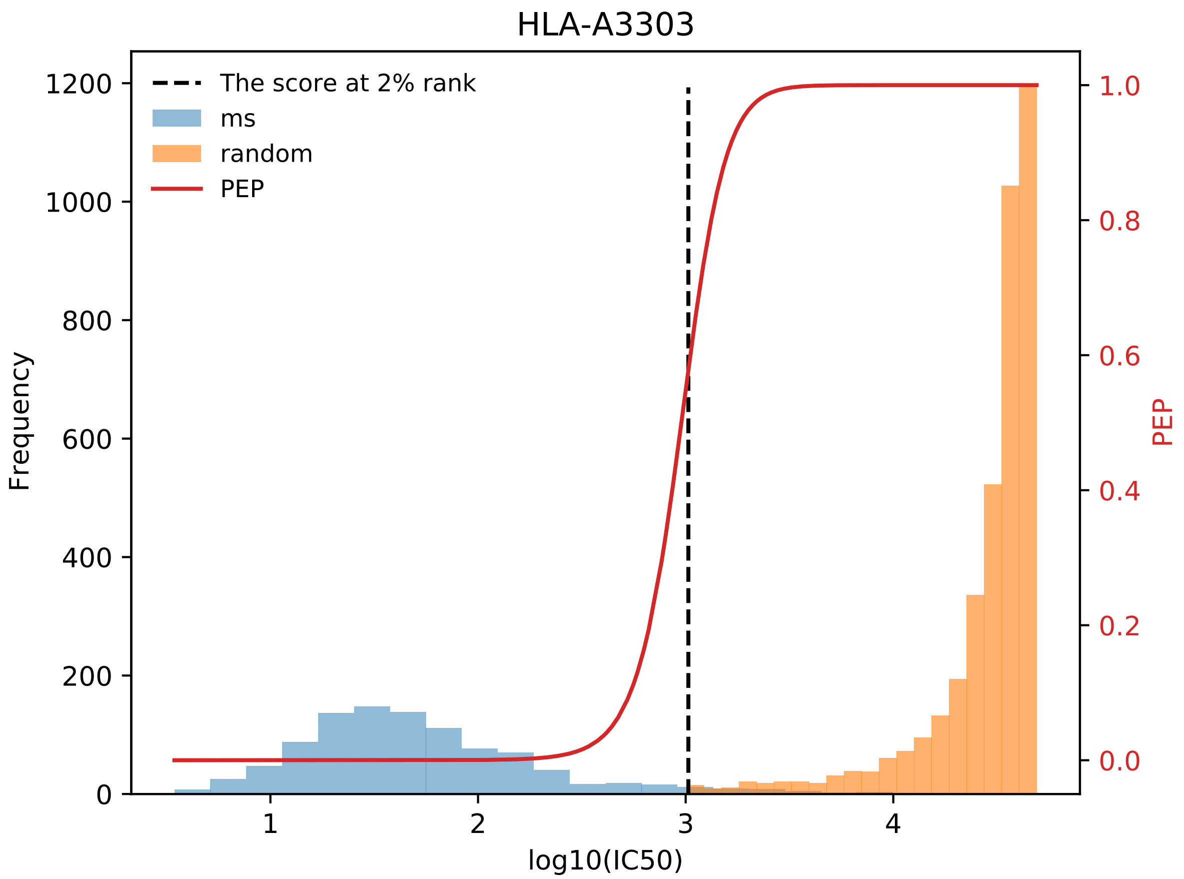
HLA-A3303
The score at 2% rank
ms
random
PEP
Frequency
0
200
400
600
800
1000
1200
1
2
3
4
log10(IC50)
0.0
0.2
0.4
0.6
0.8
1.0
PEP

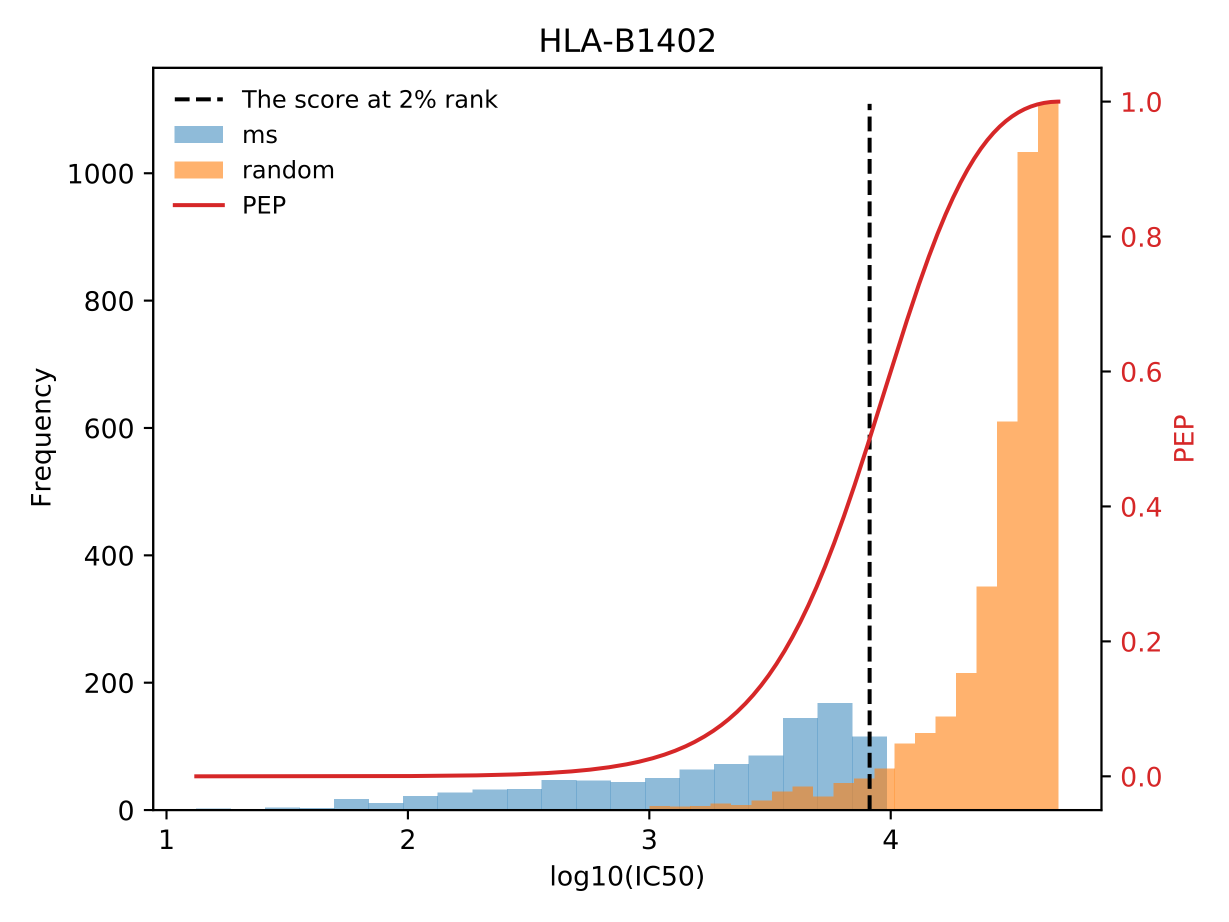

HLA-B1402
The score at 2% rank
ms
random
PEP
Frequency
0
200
400
600
800
1000
1
2
3
4
log10(IC50)
PEP
0.0
0.2
0.4
0.6
0.8
1.0

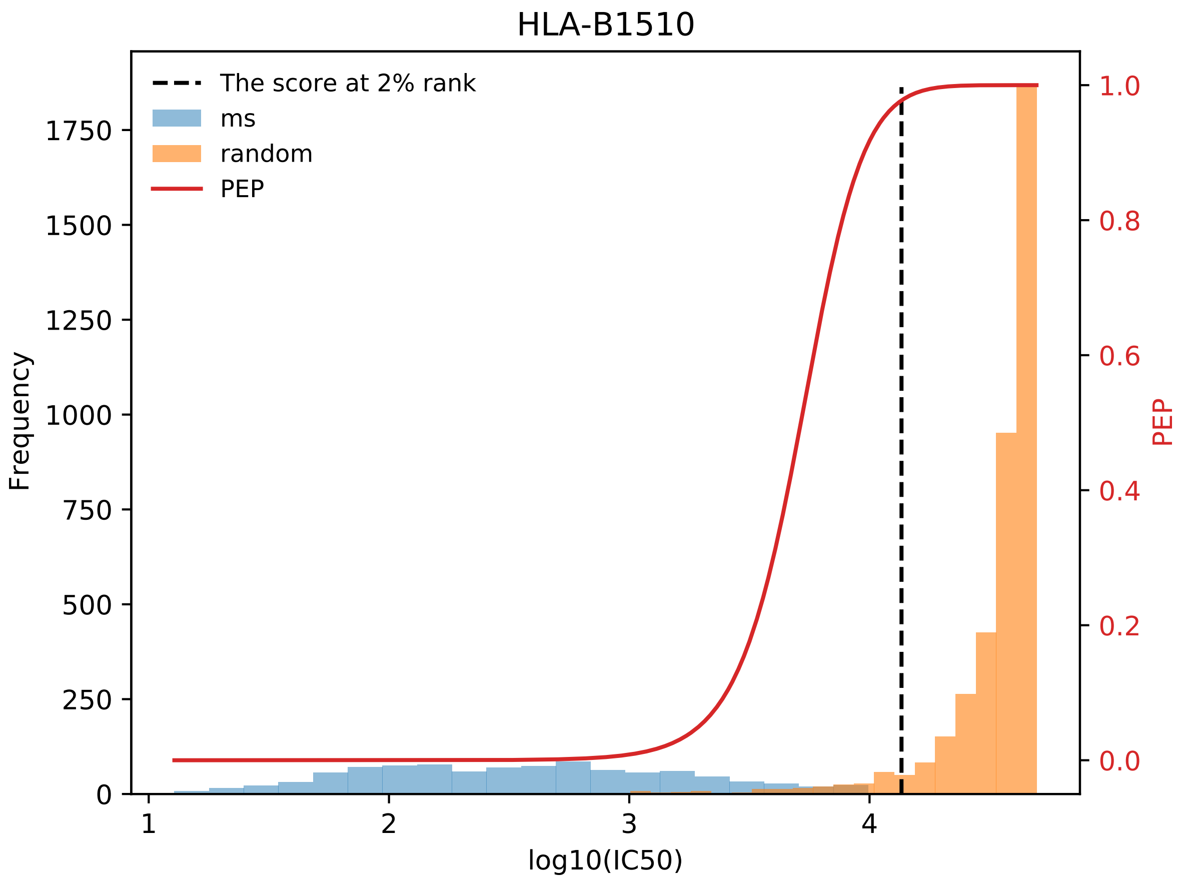
HLA-B1510
The score at 2% rank
ms
random
PEP
Frequency
0
250
500
750
1000
1250
1500
1750
1
2
3
4
log10(IC50)
0.0
0.2
0.4
0.6
0.8
1.0
PEP

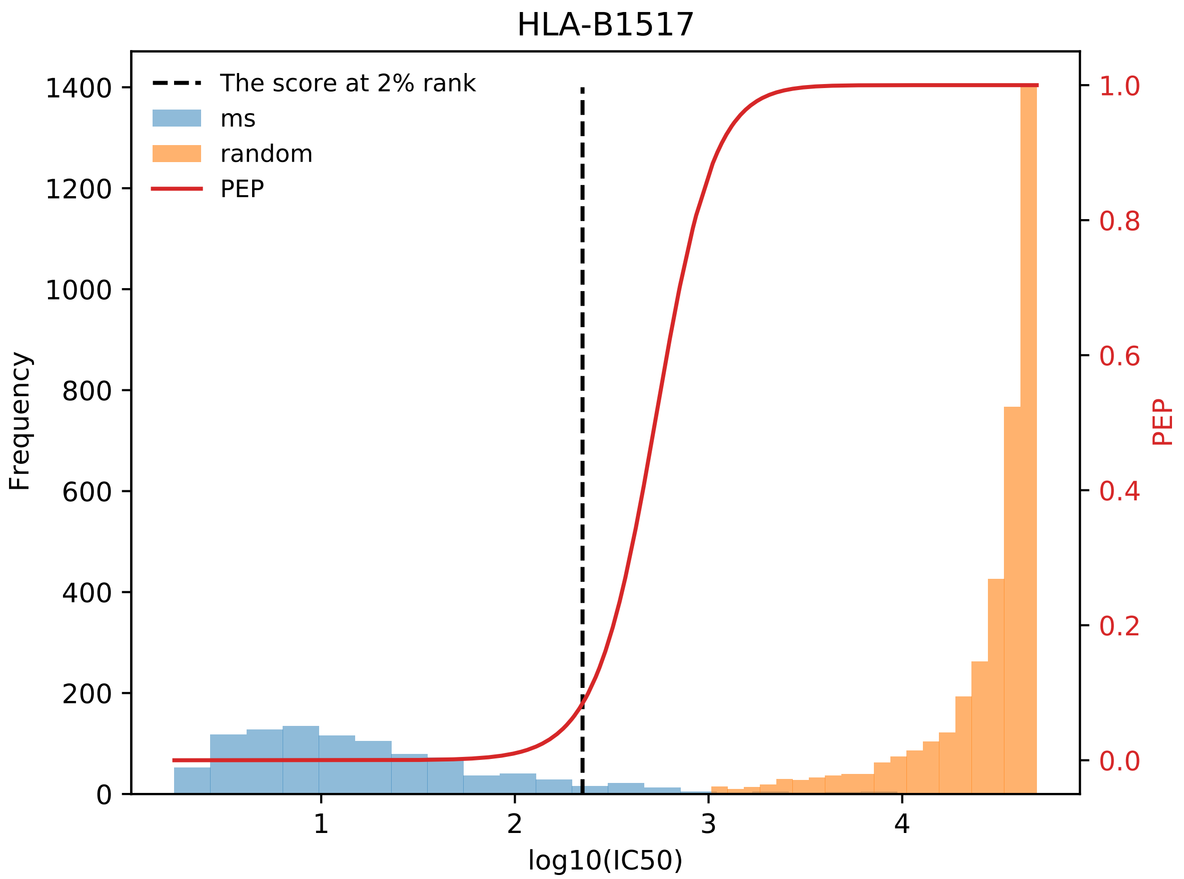
HLA-B1517
The score at 2% rank
ms
random
PEP
Frequency
0
200
400
600
800
1000
1200
1400
1
2
3
4
log10(IC50)
0.0
0.2
0.4
0.6
0.8
1.0
PEP

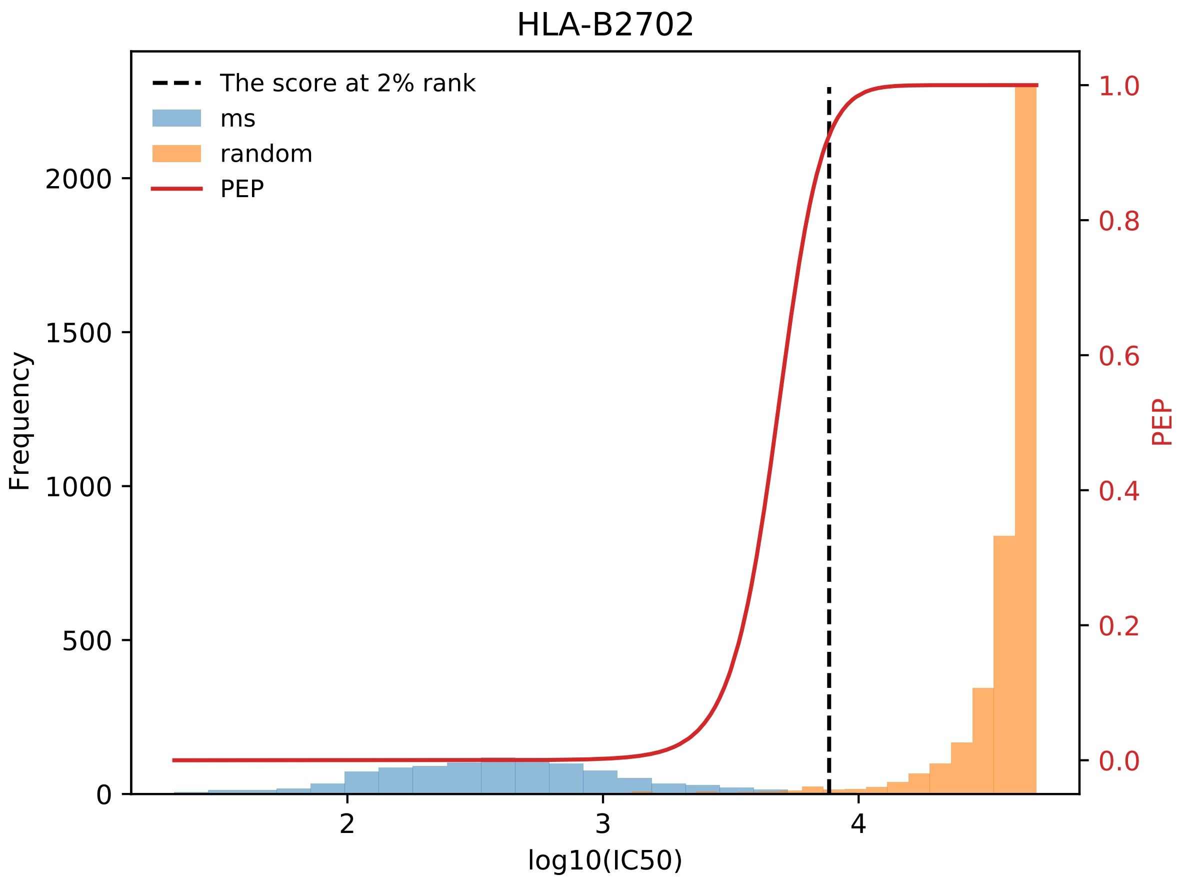

HLA-B2702
The score at 2% rank
ms
random
PEP
0
500
1000
1500
2000
Frequency
2
3
4
log10(IC50)
0.0
0.2
0.4
0.6
0.8
1.0
PEP

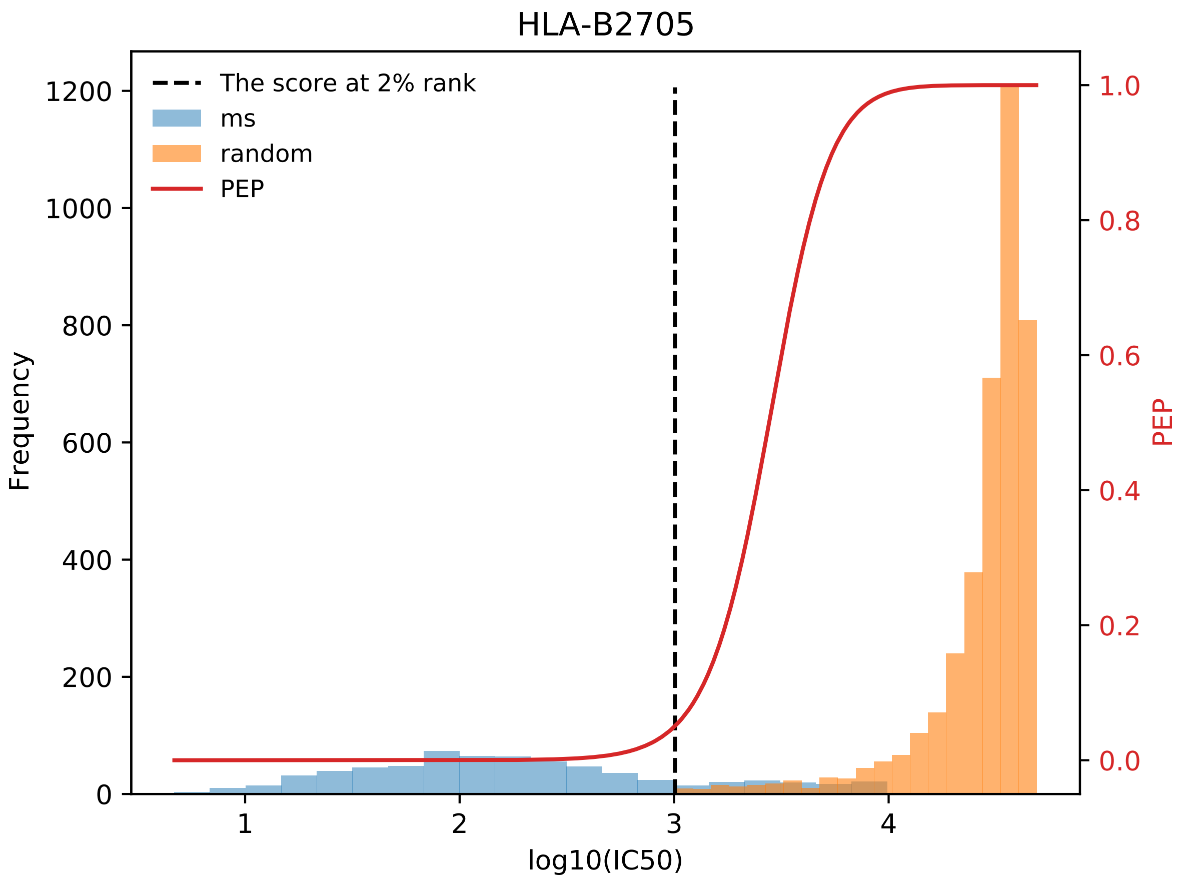

HLA-B2705
The score at 2% rank
ms
random
PEP
Frequency
0
200
400
600
800
1000
1200
1
2
3
4
log10(IC50)
0.0
0.2
0.4
0.6
0.8
1.0
PEP

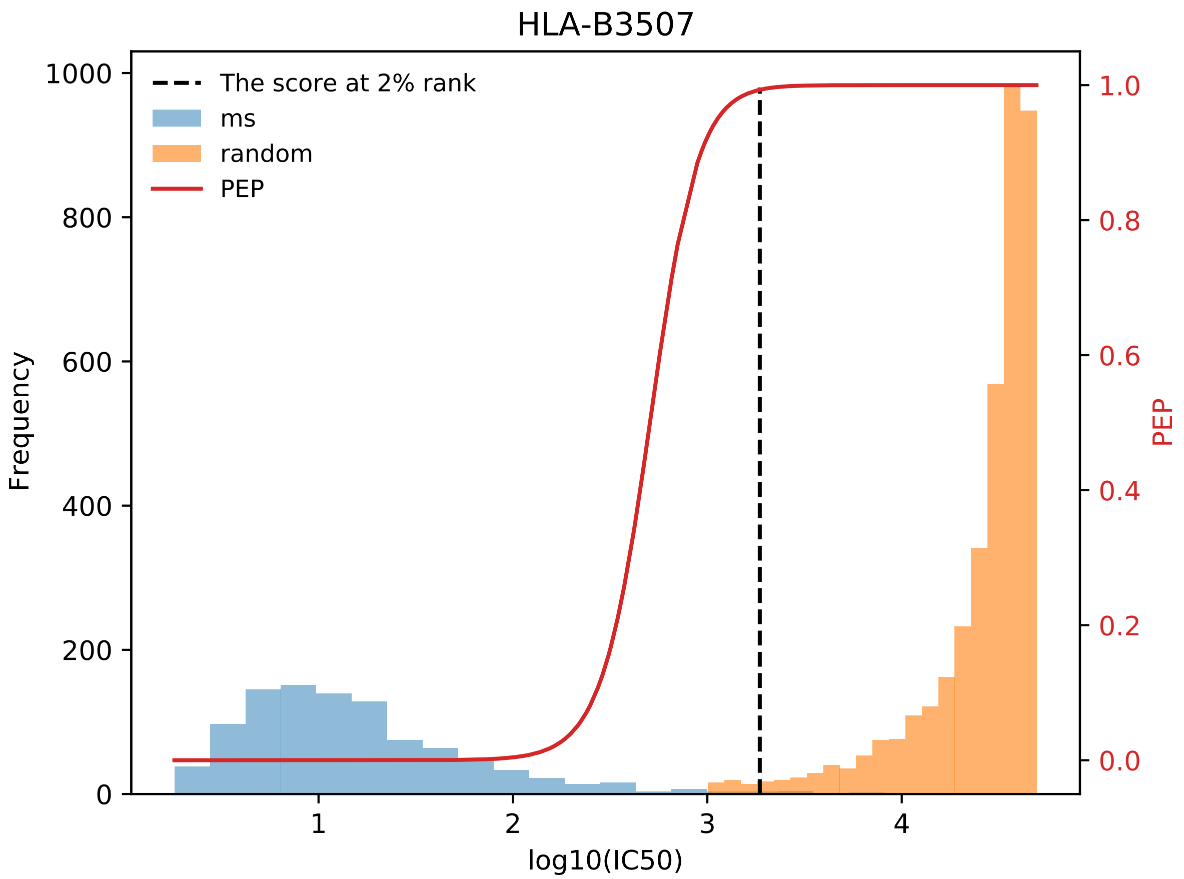
HLA-B3507
The score at 2% rank
ms
random
PEP
Frequency
0
200
400
600
800
1000
1
2
3
4
log10(IC50)
0.0
0.2
0.4
0.6
0.8
1.0
PEP

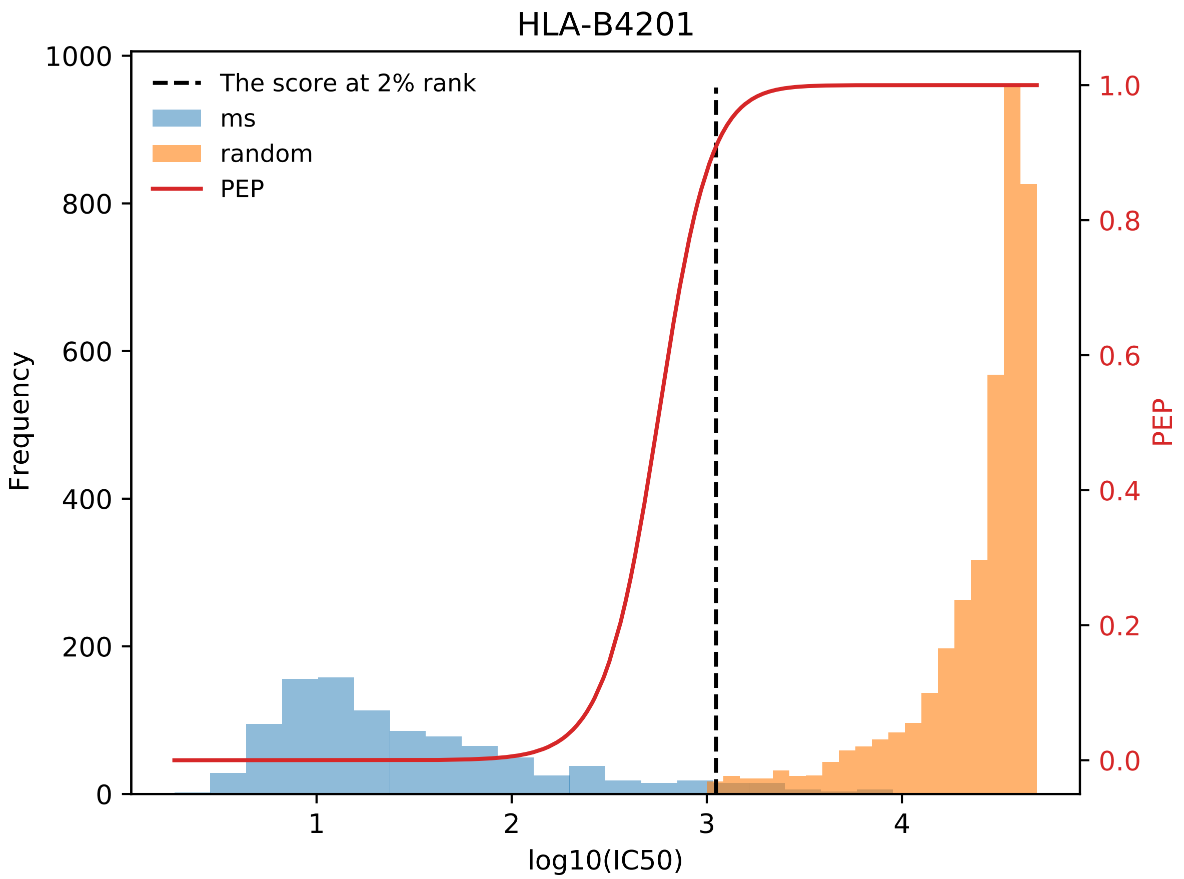

HLA-B4201
The score at 2% rank
ms
random
PEP
Frequency
0
200
400
600
800
1000
1
2
3
4
log10(IC50)
0.0
0.2
0.4
0.6
0.8
1.0
PEP

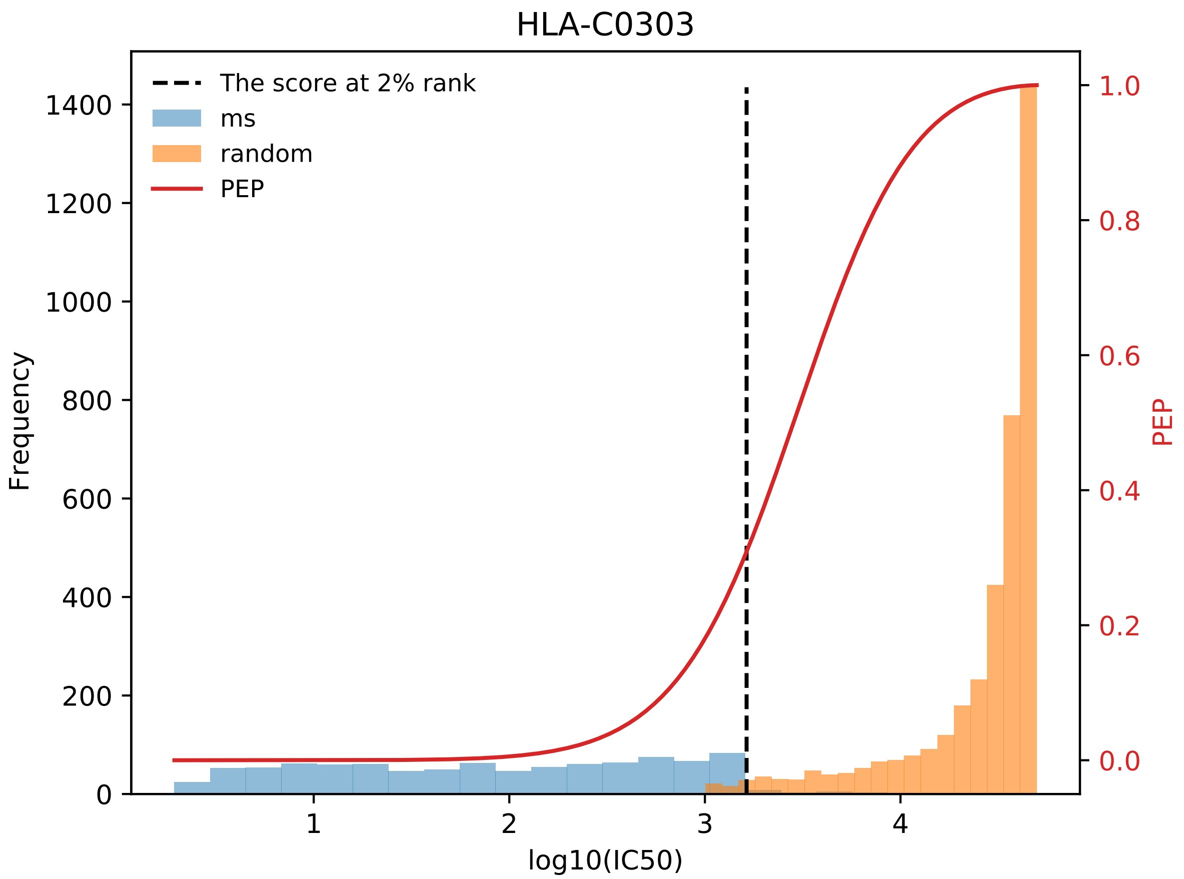
HLA-C0303
The score at 2% rank
ms
random
PEP
Frequency
PEP
log10(IC50)
0
200
400
600
800
1000
1200
1400
1
2
3
4
0.0
0.2
0.4
0.6
0.8
1.0

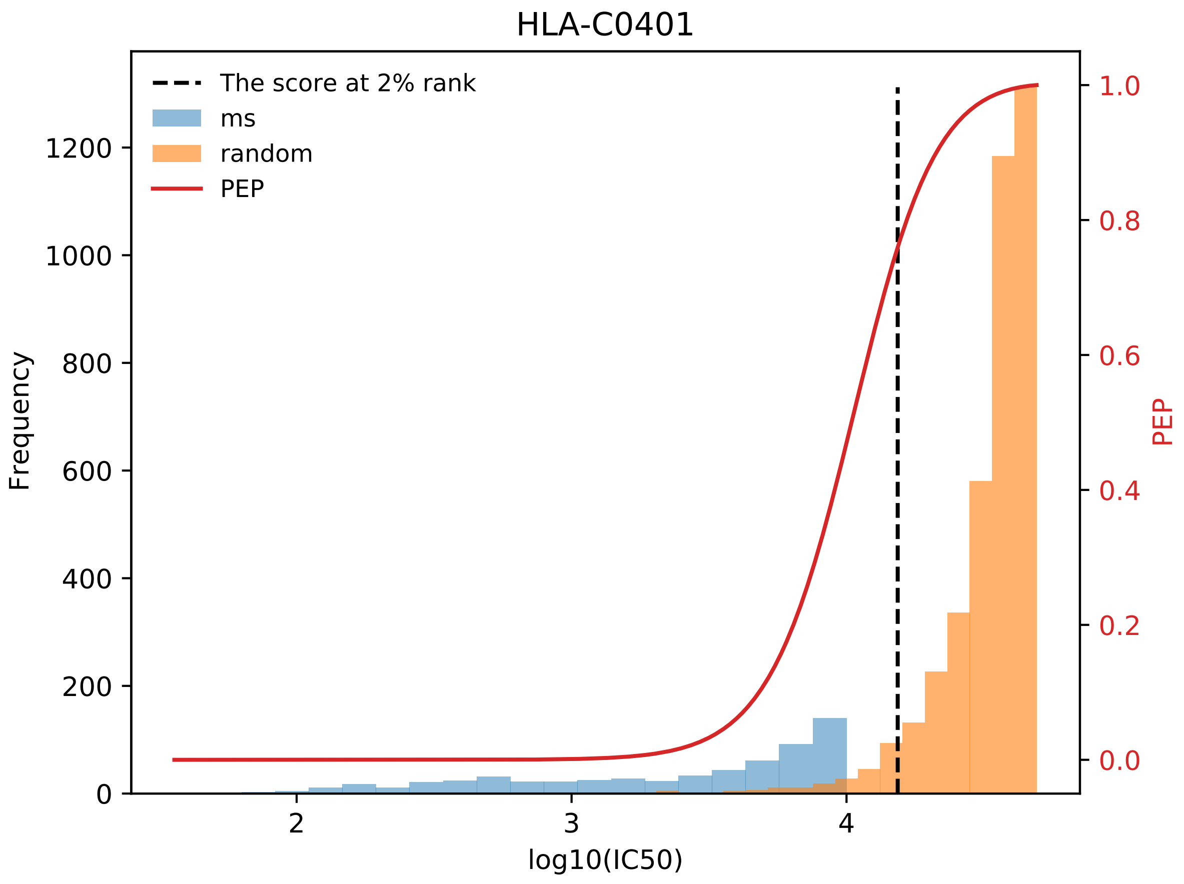
HLA-C0401
The score at 2% rank
ms
random
PEP
Frequency
0
200
400
600
800
1000
1200
2
3
4
log10(IC50)
PEP
0.0
0.2
0.4
0.6
0.8
1.0

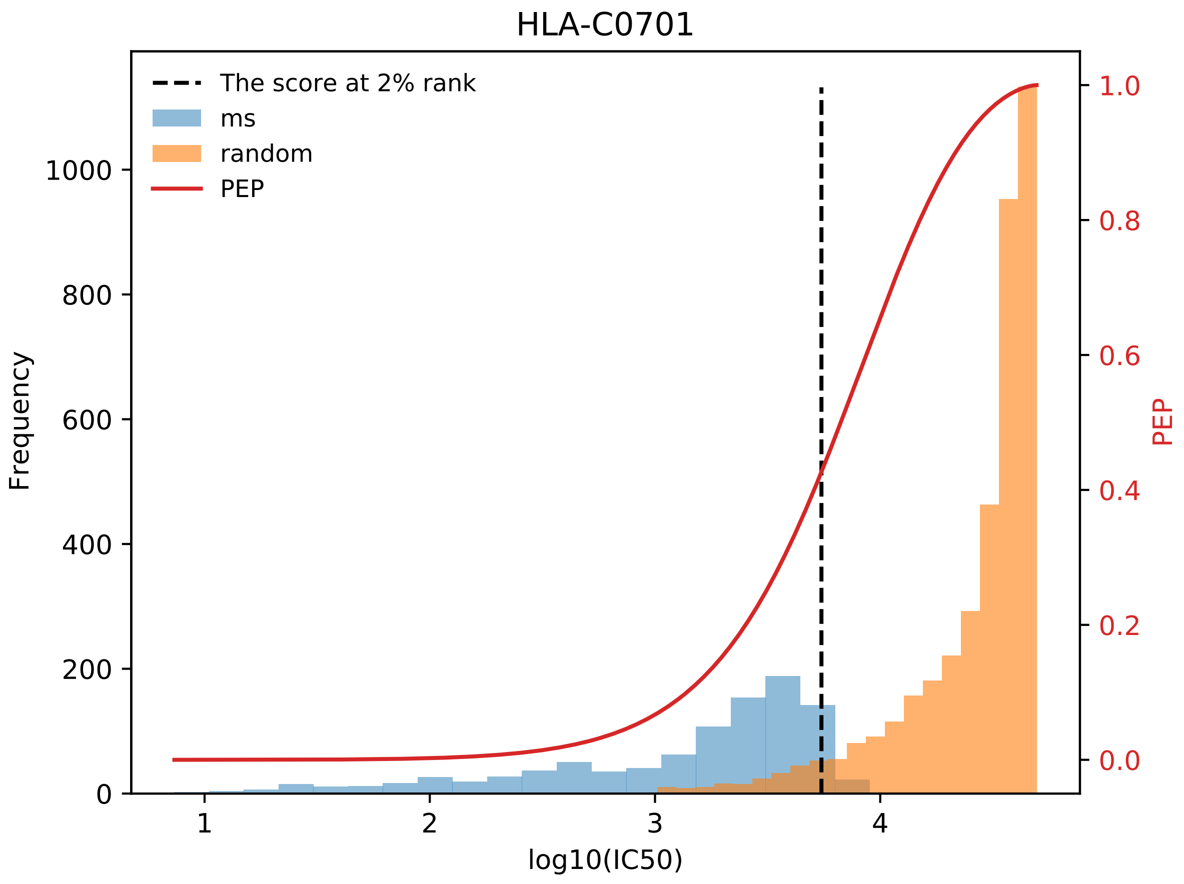

HLA-C0701
The score at 2% rank
ms
random
PEP
Frequency
0
200
400
600
800
1000
1
2
3
4
log10(IC50)
0.0
0.2
0.4
0.6
0.8
1.0
PEP

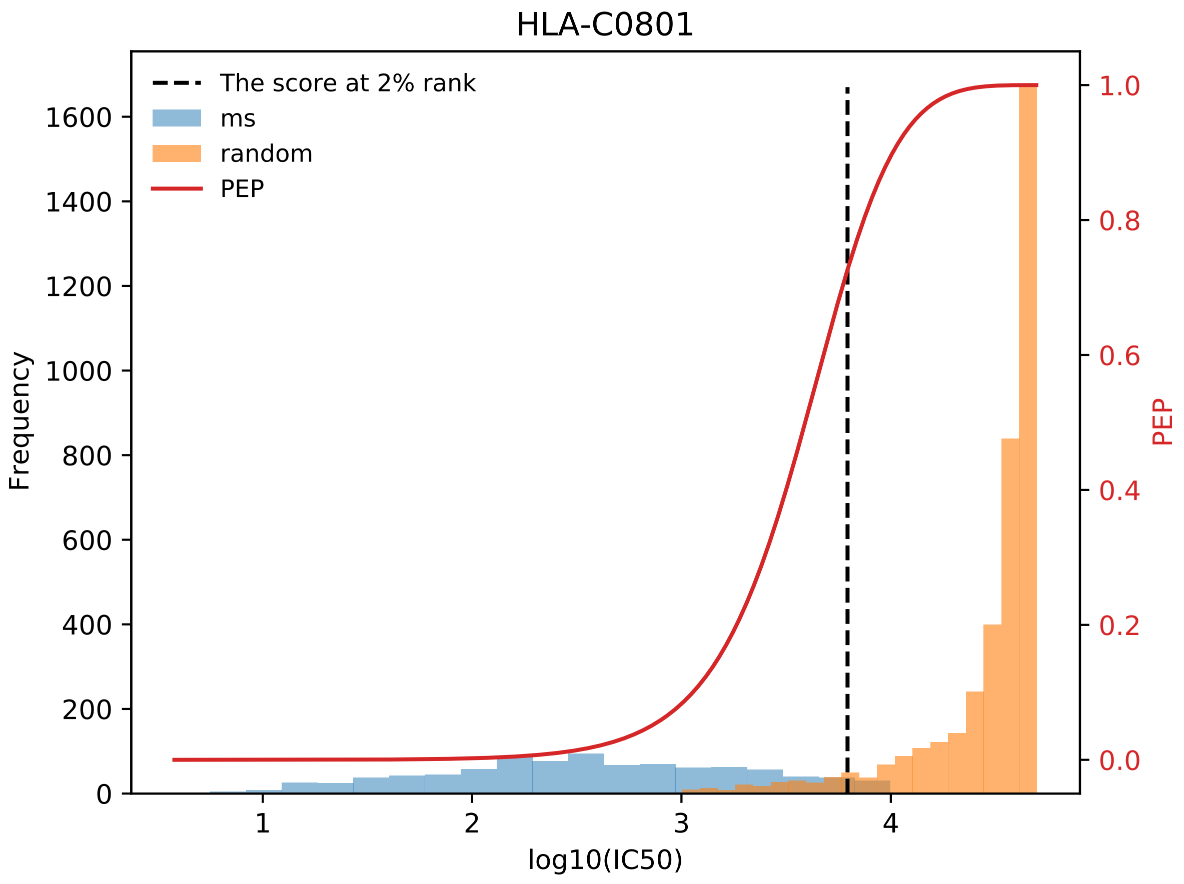

HLA-C0801
The score at 2% rank
ms
random
PEP
Frequency
0
200
400
600
800
1000
1200
1400
1600
1
2
3
4
log10(IC50)
0.0
0.2
0.4
0.6
0.8
1.0
PEP

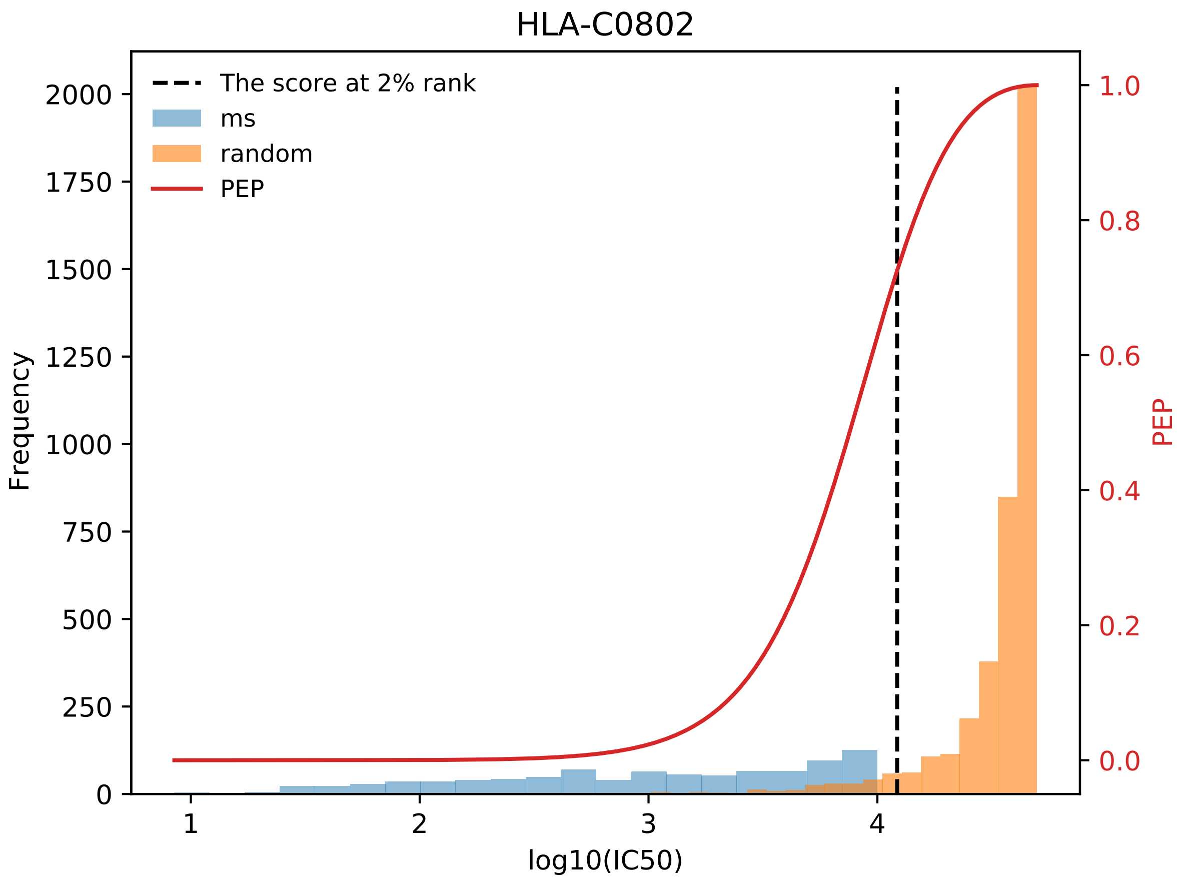

HLA-C0802
The score at 2% rank
ms
random
PEP
Frequency
2000
1750
1500
1250
1000
750
500
250
0
1
2
3
4
log10(IC50)
1.0
0.8
0.6
0.4
0.2
0.0
PEP

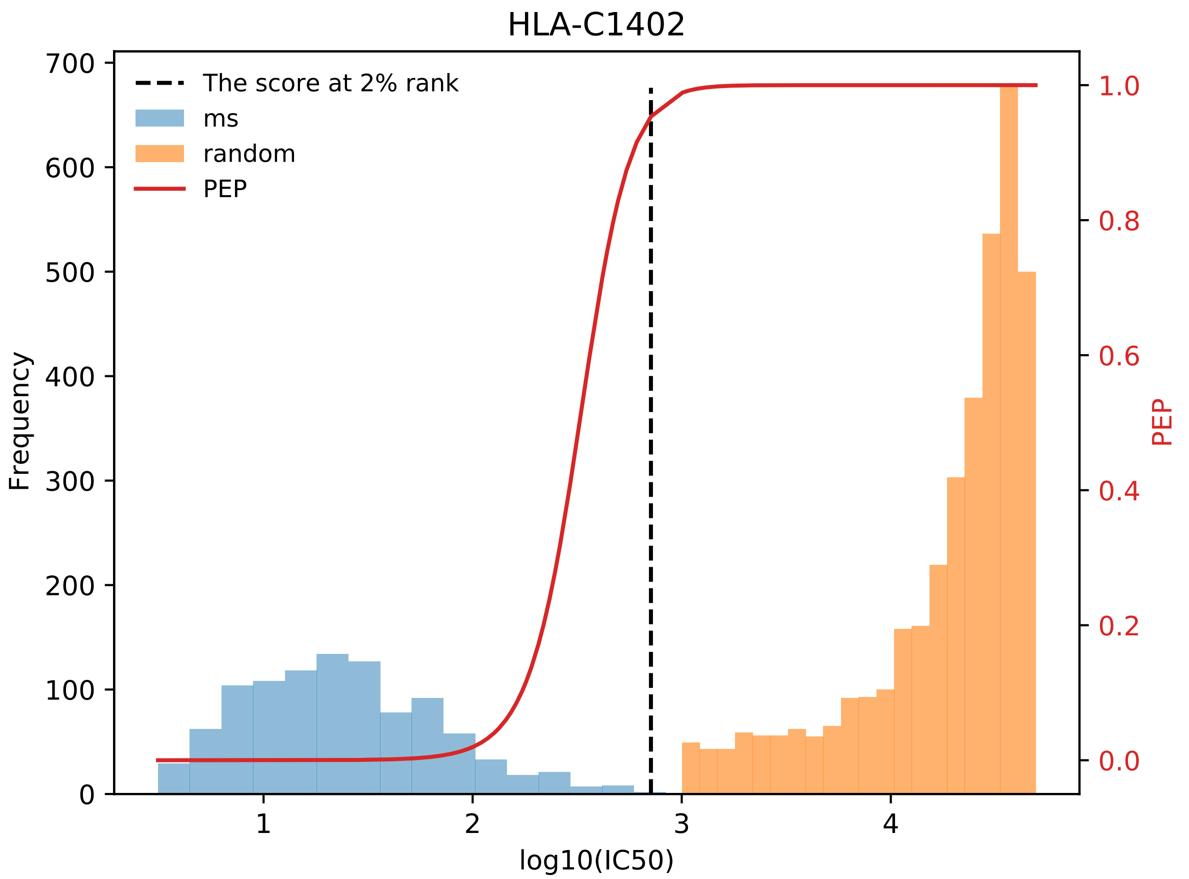

HLA-C1402
The score at 2% rank
ms
random
PEP
Frequency
0
100
200
300
400
500
600
700
1
2
3
4
log10(IC50)
0.0
0.2
0.4
0.6
0.8
1.0
PEP

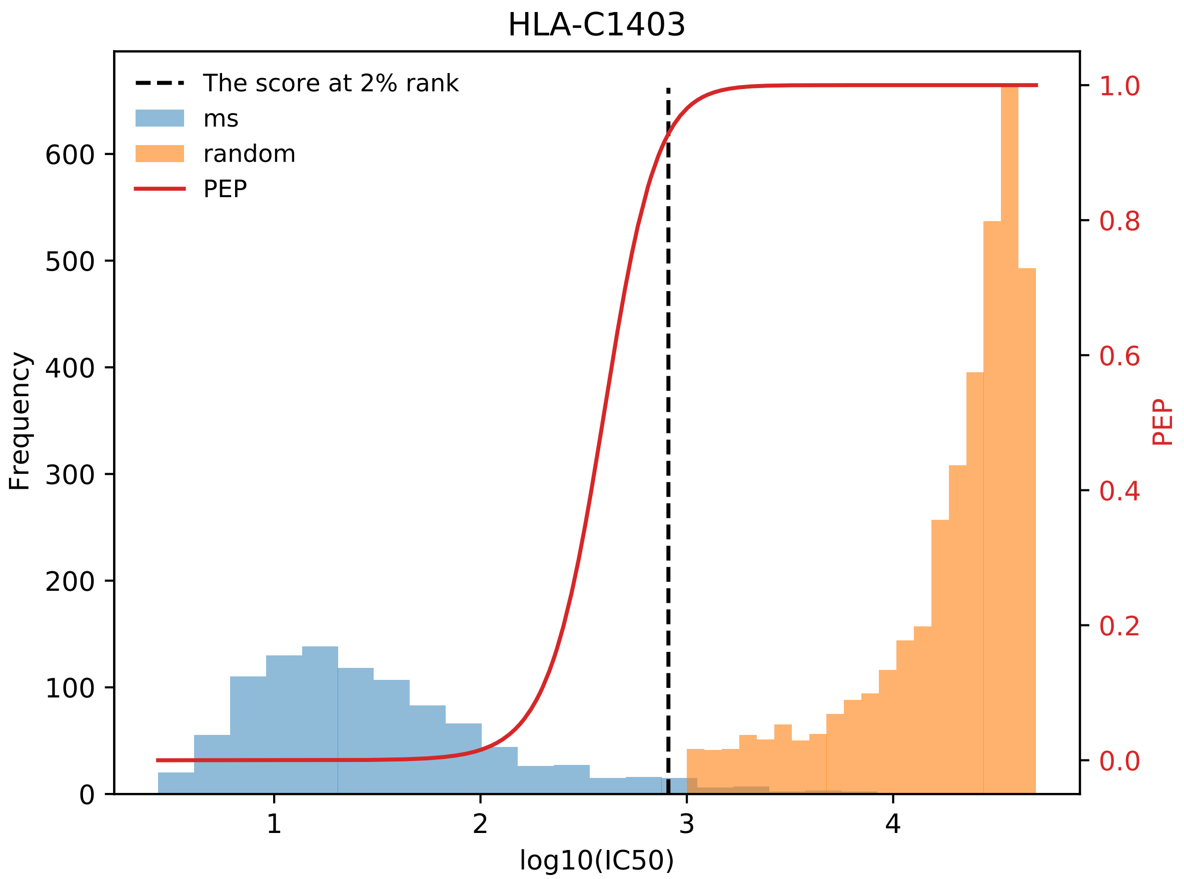

HLA-C1403
The score at 2% rank
ms
random
PEP
Frequency
0
100
200
300
400
500
600
1
2
3
4
log10(IC50)
0.0
0.2
0.4
0.6
0.8
1.0
PEP
